# Low-dimensional spike rate models derived from networks of adaptive integrate-and-fire neurons: Comparison and implementation

**DOI:** 10.1371/journal.pcbi.1005545

**Published:** 2017-06-23

**Authors:** Moritz Augustin, Josef Ladenbauer, Fabian Baumann, Klaus Obermayer

**Affiliations:** 1 Department of Software Engineering and Theoretical Computer Science, Technische Universität Berlin, Berlin, Germany; 2 Bernstein Center for Computational Neuroscience Berlin, Berlin, Germany; 3 Group for Neural Theory, Laboratoire de Neurosciences Cognitives, École Normale Supérieure, Paris, France; University of Rochester, UNITED STATES

## Abstract

The spiking activity of single neurons can be well described by a nonlinear integrate-and-fire model that includes somatic adaptation. When exposed to fluctuating inputs sparsely coupled populations of these model neurons exhibit stochastic collective dynamics that can be effectively characterized using the Fokker-Planck equation. This approach, however, leads to a model with an infinite-dimensional state space and non-standard boundary conditions. Here we derive from that description four simple models for the spike rate dynamics in terms of low-dimensional ordinary differential equations using two different reduction techniques: one uses the spectral decomposition of the Fokker-Planck operator, the other is based on a cascade of two linear filters and a nonlinearity, which are determined from the Fokker-Planck equation and semi-analytically approximated. We evaluate the reduced models for a wide range of biologically plausible input statistics and find that both approximation approaches lead to spike rate models that accurately reproduce the spiking behavior of the underlying adaptive integrate-and-fire population. Particularly the cascade-based models are overall most accurate and robust, especially in the sensitive region of rapidly changing input. For the mean-driven regime, when input fluctuations are not too strong and fast, however, the best performing model is based on the spectral decomposition. The low-dimensional models also well reproduce stable oscillatory spike rate dynamics that are generated either by recurrent synaptic excitation and neuronal adaptation or through delayed inhibitory synaptic feedback. The computational demands of the reduced models are very low but the implementation complexity differs between the different model variants. Therefore we have made available implementations that allow to numerically integrate the low-dimensional spike rate models as well as the Fokker-Planck partial differential equation in efficient ways for arbitrary model parametrizations as open source software. The derived spike rate descriptions retain a direct link to the properties of single neurons, allow for convenient mathematical analyses of network states, and are well suited for application in neural mass/mean-field based brain network models.

## Author summary

Characterizing the dynamics of biophysically modeled, large neuronal networks usually involves extensive numerical simulations. As an alternative to this expensive procedure we propose efficient models that describe the network activity in terms of a few ordinary differential equations. These systems are simple to solve and allow for convenient investigations of asynchronous, oscillatory or chaotic network states because linear stability analyses and powerful related methods are readily applicable. We build upon two research lines on which substantial efforts have been exerted in the last two decades: (i) the development of single neuron models of reduced complexity that can accurately reproduce a large repertoire of observed neuronal behavior, and (ii) different approaches to approximate the Fokker-Planck equation that represents the collective dynamics of large neuronal networks. We combine these advances and extend recent approximation methods of the latter kind to obtain spike rate models that surprisingly well reproduce the macroscopic dynamics of the underlying neuronal network. At the same time the microscopic properties are retained through the single neuron model parameters. To enable a fast adoption we have released an efficient Python implementation as open source software under a free license.

## Introduction

There is prominent evidence that information in the brain, about a particular stimulus for example, is contained in the collective neuronal spiking activity averaged over populations of neurons with similar properties (population spike rate code) [1, 2]. Although these populations can comprise a large number of neurons [3], they often exhibit low-dimensional collective spiking dynamics [4] that can be measured using neural mass signals such as the local field potential or electroencephalography.

The behavior of cortical networks at that level is often studied computationally by employing simulations of multiple (realistically large or subsampled) populations of synaptically coupled individual spiking model neurons. A popular choice of single cell description for this purpose are two-variable integrate-and-fire models [5, 6] which describe the evolution of the fast (somatic) membrane voltage and an adaptation variable that represents a slowly-decaying potassium current. These models are computationally efficient and can be successfully calibrated using electrophysiological recordings of real cortical neurons and standard stimulation protocols [5, 7–10] to accurately reproduce their subthreshold and spiking activity. The choice of such (simple) neuron models, however, does not imply reasonable (short enough) simulation durations for a recurrent network, especially when large numbers of neurons and synaptic connections between them are considered.

A fast and mathematically tractable alternative to simulations of large networks are population activity models in terms of low-dimensional ordinary differential equations (i.e., which consist of only a few variables) that typically describe the evolution of the spike rate. These reduced models can be rapidly solved and allow for convenient analyses of the dynamical network states using well-known methods that are simple to implement. A popular example are the Wilson-Cowan equations [11], which were also extended to account for (slow) neuronal adaptation [12] and short-term synaptic depression [13]. Models of this type have been successfully applied to qualitatively characterize the possible dynamical states of coupled neuronal populations using phase space analyses [11–13], yet a direct link to more biophysically described networks of (calibrated) spiking neurons in terms of model parameters is missing.

Recently, derived population activity models have been proposed that bridge the gap between single neuron properties and mesoscopic network dynamics. These models are described by integral equations [14, 15] or partial differential equations [16, 17]

Here we derive simple models in terms of low-dimensional ordinary differential equations (ODEs) for the spike rate dynamics of sparsely coupled adaptive nonlinear integrate-and-fire neurons that are exposed to noisy synaptic input. The derivations are based on a Fokker-Planck equation that describes the neuronal population activity in the mean-field limit of large networks. We develop reduced models using recent methodological advances on two different approaches: the first is based on a spectral decomposition of the Fokker-Planck operator under two different slowness assumptions [18–20]. In the second approach we consider a cascade of linear temporal filters and a nonlinear function which are determined from the Fokker-Planck equation and semi-analytically approximated, building upon [21]. Both approaches are extended for an adaptation current, a nonlinear spike generating current and recurrent coupling with distributed synaptic delays.

We evaluate the developed low-dimensional spike rate models quantitatively in terms of reproduction accuracy in a systematic manner over a wide range of biologically plausible parameter values. In addition, we provide numerical implementations for the different reduction methods as well as the Fokker-Planck equation under a free license as open source project.

For the derived models in this contribution we use the adaptive exponential integrate-and-fire (aEIF) model [5] to describe individual neurons, which is similar to the model proposed by Izhikevich [6] but includes biophysically meaningful parameters and a refined description of spike initiation. However, the presented derivations are equally applicable when using the Izhikevich model instead (requiring only a small number of simple substitutions in the code).

Through their parameters the derived models retain a direct, quantitative link to the underlying spiking model neurons, and they are described in a well-established, convenient form (ODEs) that can be rapidly solved and analyzed. Therefore, these models are well suited (i) for mathematical analyses of dynamical states at the population level, e.g., linear stability analyses of attractors, and (ii) for application in multi-population brain network models. Apart from a specific network setting, the derived models are also appropriate as a spike rate description of individual neurons under noisy input conditions.

The structure of this article contains mildly redundant model specifications allowing the readers who are not interested in the methodological foundation to directly read the self-contained Sect. Results.

## Results

### Model reduction

The quantity of our interest is the population-averaged number of spikes emitted by a large homogeneous network of *N* sparsely coupled aEIF model neurons per small time interval, i.e., the spike rate *r*_*N*_(*t*). The state of neuron *i* at time *t* is described by the membrane voltage *V*_*i*_(*t*) and adaptation current *w*_*i*_(*t*), which evolve piecewise continuously in response to overall synaptic current *I*_syn,*i*_ = *I*_ext,*i*_(*t*) + *I*_rec,*i*_(*t*). This input current consists of fluctuating network-external drive *I*_ext,*i*_ = *C*[*μ*_ext_(*t*) + *σ*_ext_(*t*)*ξ*_ext,*i*_(*t*)] with membrane capacitance *C*, time-varying moments *μ*_ext_, $\sigma_{\text{ext}}^{2}$ and unit Gaussian white noise process *ξ*_ext,*i*_ as well as recurrent input *I*_rec,i_. The latter causes delayed postsynaptic potentials (i.e., deflections of *V*_*i*_) of small amplitude *J* triggered by the spikes of *K* presynaptic neurons (see Sect. Methods for details).

Here we present two approaches of how the spike rate dynamics of the large, stochastic delay-differential equation system for the 2*N* states (*V*_*i*_, *w*_*i*_) can be described by simple models in terms of low-dimensional ODEs. Both approaches (i) take into account adaptation current dynamics that are sufficiently slow, allowing to replace the individual adaptation current *w*_*i*_ by its population-average 〈*w*〉, governed by

$$\frac{d\left\langle w\right\rangle }{dt}=\frac{a({\left\langle V\right\rangle }_{\infty }-E_{w})-\left\langle w\right\rangle }{\tau_{w}}+b\;r\left(t\right),$$ (1)

where *a*, *E*_*w*_, *b*, *τ*_*w*_ are the adaptation current model parameters (subthreshold conductance, reversal potential, spike-triggered increment, time constant, respectively), 〈*V*〉_∞_ is the steady-state membrane voltage averaged across the population (which can vary over time, see below), and *r* is the spike rate of the respective low-dimensional model. Furthermore, both approaches (ii) are based on the observation that the collective dynamics of a large, sparsely coupled (and noise driven) network of integrate-and-fire type neurons can be well described by a Fokker-Planck equation. In this *intermediate* Fokker-Planck (FP) model the overall synaptic input is approximated by a mean part with additive white Gaussian fluctuations, *I*_syn,i_/*C* ≈ *μ*_syn_(*t*, *r*_*d*_) + *σ*_syn_(*t*, *r*_*d*_)*ξ*_*i*_(*t*), that are uncorrelated between neurons. The moments of the overall synaptic input,

$$\mu_{\text{syn}}=\mu_{\text{ext}}\left(t\right)+JKr_{d}\left(t\right),\;\sigma_{\text{syn}}^{2}=\sigma_{\text{ext}}^{2}\left(t\right)+J^{2}Kr_{d}\left(t\right),$$ (2)

depend on time via the moments of the external input and, due to recurrent coupling, on the delayed spike rate *r*_*d*_. The latter is governed by

$$\frac{dr_{d}}{dt}=\frac{r-r_{d}}{\tau_{d}},$$ (3)

which corresponds to individual propagation delays drawn from an exponentially distributed random variable with mean *τ*_*d*_. The FP model involves solving a partial differential equation (PDE) to obtain the time-varying membrane voltage distribution *p*(*V*, *t*) and the spike rate *r*(*t*).

The first reduction approach is based on the spectral decomposition of the Fokker-Planck operator L and leads to the following two low-dimensional models: the “basic” model variant (spec_1_) is given by a complex-valued differential equation describing the spike rate evolution in its real part,

$$\frac{d\tilde{r}}{dt}=\lambda_{1}\left(\tilde{r}-r_{\infty }\right),\;r\left(t\right)=\text{Re}\left\{\tilde{r}\right\},$$ (4)

where λ_1_(*μ*_tot_, *σ*_tot_) is the dominant eigenvalue of L and *r*_∞_(*μ*_tot_, *σ*_tot_) is the steady-state spike rate. Its parameters λ_1_, *r*_∞_, and 〈*V*〉_∞_ (cf. Eq (1)) depend on the *total* input moments given by *μ*_tot_(*t*) = *μ*_syn_ − 〈*w*〉/*C* and $\sigma_{\text{tot}}^{2}\left(t\right)=\sigma_{\text{syn}}^{2}$ which closes the model (Eqs (1)–(4)). The other, “advanced” spectral model variant (spec_2_) is given by a real-valued second order differential equation for the spike rate,

$$\beta_{2}\;\ddot{r}+\beta_{1}\;\dot{r}+\beta_{0}\;r=r_{\infty }-r-\beta_{c},$$ (5)

where the dots denote time derivatives. Its parameters *β*_2_, *β*_1_, *β*_0_, *β*_*c*_, *r*_∞_ and 〈*V*〉_∞_ depend on the total input moments (*μ*_tot_, $\sigma_{\text{tot}}^{2}$) as follows: the latter two parameters explicitly as in the basic model above, the former four indirectly via the first two dominant eigenvalues λ_1_, λ_2_ and via additional quantities obtained from the (stationary and the first two nonstationary) eigenfunctions of L and its adjoint $L^{*}$. Furthermore, the parameter *β*_*c*_ depends explicitly on the population-averaged adaptation current 〈*w*〉, the delayed spike rate *r*_*d*_, and on the first and second order time derivatives of the external moments *μ*_ext_ and $\sigma_{\text{ext}}^{2}$.

The second approach is based on a Linear-Nonlinear (LN) cascade, in which the population spike rate is generated by applying to the time-varying mean and standard deviation of the overall synaptic input, *μ*_syn_ and *σ*_syn_, separately a linear temporal filter, followed by a common nonlinear function. These three components–two linear filters and a nonlinearity–are extracted from the Fokker-Planck equation. Approximating the linear filters using exponentials and damped oscillating functions yields two model variants: In the basic “exponential” (LN_exp_) model the filtered mean *μ*_f_ and standard deviation *σ*_f_ of the overall synaptic input are given by

$$\frac{d\mu_{f}}{dt}=\frac{\mu_{\text{syn}}-\mu_{f}}{\tau_{\mu }},\;\frac{d\sigma_{f}}{dt}=\frac{\sigma_{\text{syn}}-\sigma_{f}}{\tau_{\sigma }},$$ (6)

where the time constants *τ*_*μ*_(*μ*_eff_, *σ*_eff_), *τ*_*σ*_(*μ*_eff_, *σ*_eff_) depend on the *effective* (filtered) input mean *μ*_eff_(*t*) = *μ*_f_ − 〈*w*〉/*C* and standard deviation *σ*_eff_(*t*) = *σ*_f_. The “damped oscillator” (LN_dos_) model variant, on the other hand, describes the filtered input moments by

$${\ddot{\mu }}_{f}+\frac{2}{\tau }{\dot{\mu }}_{f}+(\frac{2}{\tau^{2}}+\omega^{2})\;\mu_{f}=\frac{1+\tau^{2}\omega^{2}}{\tau }\;(\frac{\mu_{\text{syn}}}{\tau }+{\dot{\mu }}_{\text{syn}}),$$ (7)

$$\frac{d\sigma_{f}}{dt}=\frac{\sigma_{\text{syn}}-\sigma_{f}}{\tau_{\sigma }},$$ (8)

where the time constants *τ*(*μ*_tot_, *σ*_tot_), *τ*_*σ*_(*μ*_tot_, *σ*_tot_) and the angular frequency *ω*(*μ*_tot_, *σ*_tot_) depend on the *total* input moments defined above. In both LN model variants the spike rate is obtained by the nonlinear transformation of the *effective* input moments through the steady-state spike rate,

$$r\left(t\right)=r_{\infty }\;(\mu_{\text{eff}},\;\sigma_{\text{eff}}),$$ (9)

and the steady-state mean membrane voltage 〈*V*〉_∞_ (cf. Eq (1)) is also evaluated at (*μ*_eff_, *σ*_eff_).

These four models (spec_1_, spec_2_, LN_exp_, LN_dos_) from both reduction approaches involve a number of parameters that depend on the strengths of synaptic input and adaptation current only via the *total* or *effective* input moments. We refer to these parameters as *quantities* below to distinguish them from fixed (independent) parameters. The computational complexity when numerically solving the models forward in time (for different parametrizations) can be greatly reduced by precomputing those quantities for a range of values for the *total*/*effective* input moments and using look-up tables during time integration. Changing any parameter value of the external input, the recurrent coupling or the adaptation current does not require renewed precomputations, enabling rapid explorations of parameter space and efficient (linear) stability analyses of network states.

**Fig 1:**
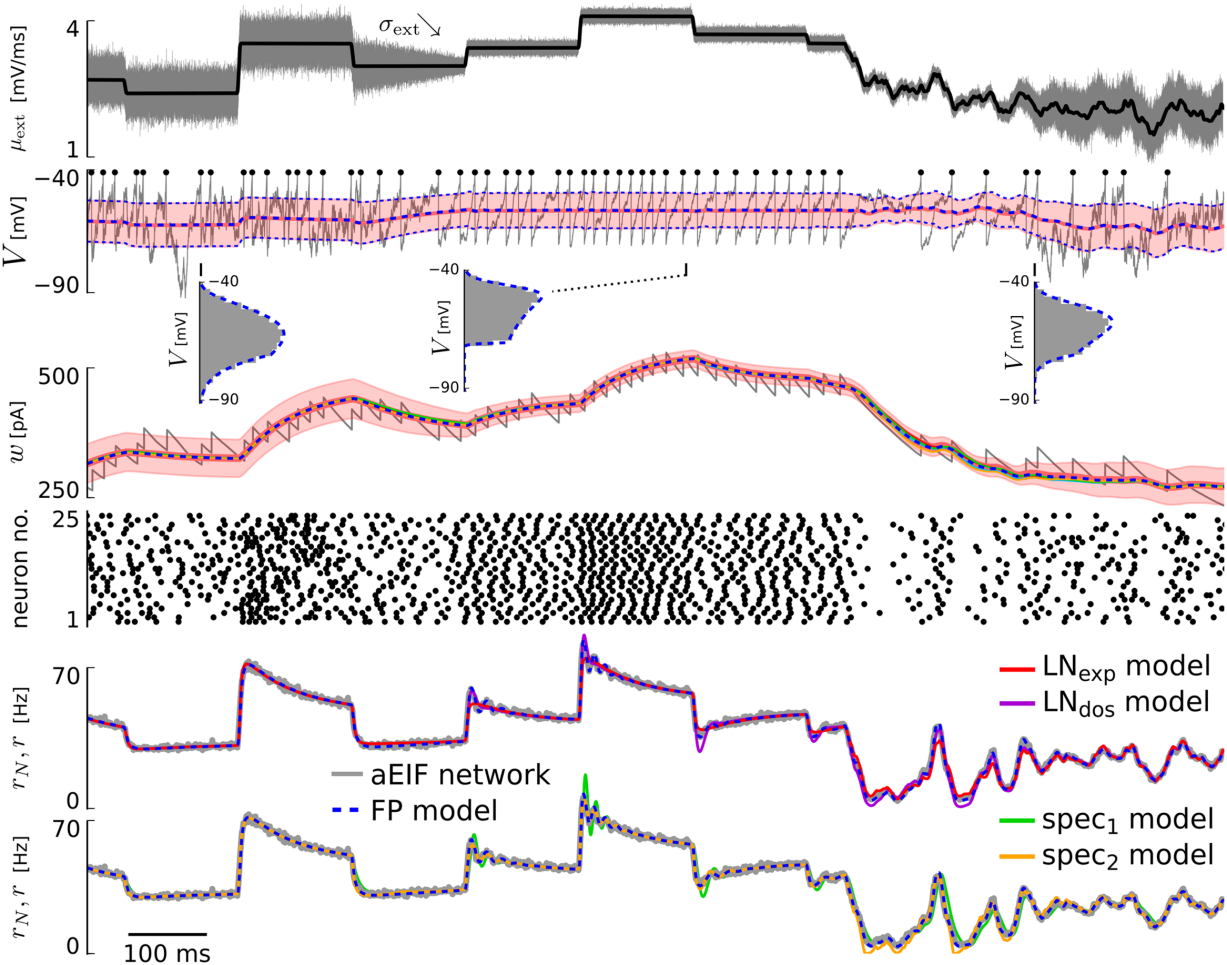
Example of aEIF network response and output of derived models for varying input. From top to bottom: Mean input *μ*_ext_ (black) together with input standard deviation *σ*_ext_ (gray, visualized for one neuron by sampling the respective white noise process *ξ*_ext,i_). 2^nd^ row: Membrane voltage *V* of one neuron (gray, with spike times highlighted by black dots) and membrane voltage statistics from the excitatory coupled aEIF population of 50,000 neurons (red) and from the FP model (blue dashed): mean ± standard deviation over time, as well as voltage histograms (gray) and probability densities *p*(*V*, *t*) (blue dashed) at three indicated time points. 3^rd^ row: Adaptation current *w* of one neuron (gray) and mean adaptation currents of all models ± standard deviation for the aEIF network (shaded area). Note that differences in the mean adaptation currents of the different models are hardly recognizable. 4^th^ row: Spike times of a subset of 25 neurons randomly chosen from the network. Below: Spike rate *r* of the LN cascade based models (LN_exp_, LN_dos_) and the spectral models (spec_1_, spec_2_) in comparison to the FP model and the aEIF network (*r*_*N*_). The values of the coupling parameters were *J* = 0.05 mV, *K* = 100, *τ*_*d*_ = 3 ms.

The full specification of the “ground truth” system (network of aEIF neurons), the derivations of the intermediate description (FP model) and the low-dimensional spike rate models complemented by concrete numerical implementations are provided in Sect. Methods (that is complemented by the supporting material S1 Text). In Fig 1 we visualize the outputs of the different models using an example excitatory aEIF network exposed to external input with varying mean *μ*_ext_(*t*) and standard deviation *σ*_ext_(*t*).

### Performance for variations of the mean input

Here, and in the subsequent two sections, we assess the accuracy of the four low-dimensional models to reproduce the spike rate dynamics of the underlying aEIF population. The *intermediate* FP model is included for reference. The derived models generate population activity in response to overall synaptic input moments *μ*_syn_ and $\sigma_{\text{syn}}^{2}$. These depend on time via the external moments *μ*_ext_(*t*) and $\sigma_{\text{ext}}^{2}\left(t\right)$, and the delayed spike rate *r*_*d*_(*t*). Therefore, it is instrumental to first consider an uncoupled population and suitable variations of external input moments that effectively mimic a range of biologically plausible presynaptic spike rate dynamics. This allows us to systematically compare the reproduction performance of the different models over a manageable parameter space (without *K*, *J*, *τ*_*d*_), yet it provides useful information on the accuracy for recurrent networks.

For many network settings the dominant effect of synaptic coupling is on the mean input (cf. Eq (2)). Therefore, we consider first in detail time-varying mean but constant variance of the input. Specifically, to account for a wide range of oscillation frequencies for presynaptic spike rates, *μ*_ext_ is described by an Ornstein-Uhlenbeck (OU) process

$${\dot{\mu }}_{\text{ext}}=\frac{\bar{\mu }-\mu_{\text{ext}}}{\tau_{\text{ou}}^{\mu }}+\sqrt{\frac{2}{\tau_{\text{ou}}^{\mu }}}\vartheta_{\mu }\xi \left(t\right),$$ (10)

where $\tau_{\text{ou}}^{\mu }$ denotes the correlation time, $\bar{\mu }$ and *ϑ*_*μ*_ are the mean and standard deviation of the stationary normal distribution, i.e., $\lim_{t\to \infty }\mu_{\text{ext}}\left(t\right)∼N\left(\bar{\mu },\vartheta_{\mu }^{2}\right)$, and *ξ* is a unit Gaussian white noise process. Sample time series generated from the OU process are filtered using a Gaussian kernel with a small standard deviation *σ*_*t*_ to obtain sufficiently differentiable time series ${\tilde{\mu }}_{\text{ext}}$ (due to the requirements of the spec_2_ model and the LN_dos_ model). The filtered realization ${\tilde{\mu }}_{\text{ext}}\left(t\right)$ is then used for all models to allow for a quantitative comparison of the different spike rate responses to the same input. The value of *σ*_*t*_ we use in this study effectively removes very large oscillation frequencies which are rarely observed, while lower frequencies [22] are passed.

The parameter space we explore covers large and small correlation times $\tau_{\text{ou}}^{\mu }$, strong and weak input mean $\bar{\mu }$ and standard deviation *σ*_ext_, and for each of these combinations we consider an interval from small to large variation magnitudes *ϑ*_*μ*_. The values of $\tau_{\text{ou}}^{\mu }$ and *ϑ*_*μ*_ determine how rapid and intense *μ*_ext_(*t*) fluctuates.

We apply two performance measures, as in [21]. One is given by Pearson’s correlation coefficient,

$$\rho \left(r_{N},r\right)≔\frac{\sum_{k=1}^{M}\;\left(r_{N}\left(t_{k}\right)-{\bar{r}}_{N}\right)\;(r\left(t_{k}\right)-\bar{r})}{\sqrt{\sum_{k=1}^{M}\;{\left(r_{N}\left(t_{k}\right)-{\bar{r}}_{N}\right)}^{2}\;\sum_{k=1}^{M}\;{\left(r\left(t_{k}\right)-\bar{r}\right)}^{2}}},$$ (11)

between the (discretely given) spike rates of the aEIF population and each derived model with time averages ${\bar{r}}_{N}=1/M\;\sum_{k=1}^{M}\;r_{N}\left(t_{k}\right)$ and $\bar{r}=1/M\;\sum_{k=1}^{M}\;r\left(t_{k}\right)$ over a time interval of length *t*_*M*_ − *t*_1_. For comparison, we also include the correlation coefficient between the aEIF population spike rate and the time-varying mean input, *ρ*(*r*_*N*_, *μ*_ext_). In addition, to assess absolute differences we calculate the root mean square (RMS) distance,

$${\text{d}}_{\text{RMS}}\left(r_{N},r\right)≔\sqrt{\frac{1}{M}\sum_{k=1}^{M}{\left(r_{N}\left(t_{k}\right)-r\left(t_{k}\right)\right)}^{2}},$$ (12)

where *M* denotes the number of elements of the respective time series (*r*_*N*_, *r*).

**Fig 2:**
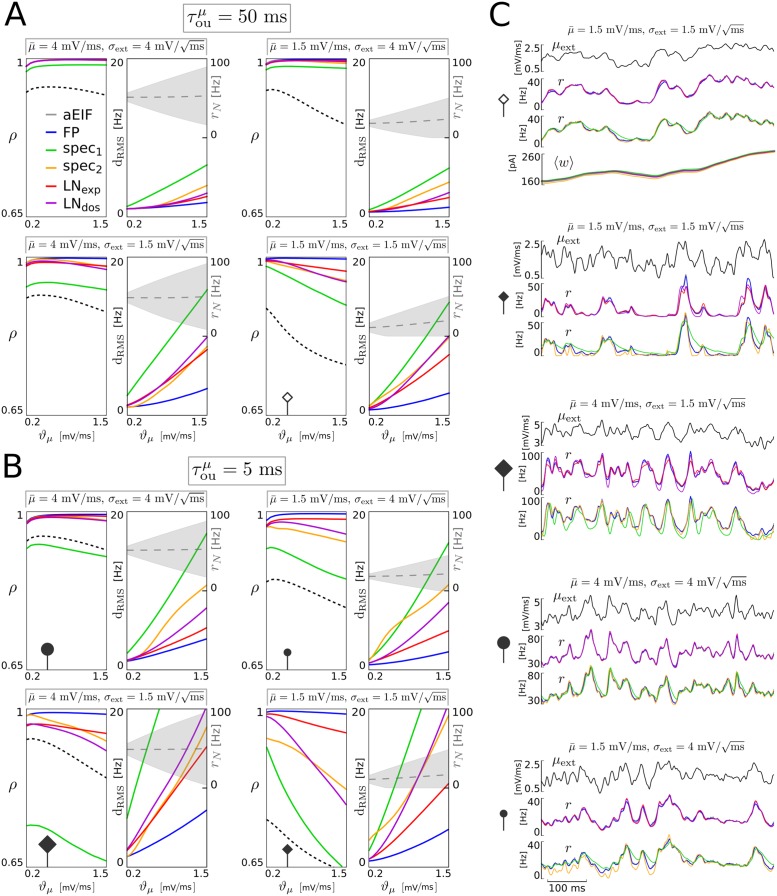
Reproduction accuracy of the reduced models for variations of the mean input. Pearson correlation coefficient (*ρ*) and root mean square distance (*d*_RMS_) between the spike rate time series *r*_*N*_(*t*) of the aEIF population and *r*(*t*) of each derived model (FP, spec_1_, spec_2_, LN_exp_, LN_dos_) for different strengths of baseline mean input $\bar{\mu }$, input standard deviation *σ*_ext_, mean input variation *ϑ*_*μ*_ and for a large value of time constant $\tau_{\text{ou}}^{\mu }$ (**A**, moderately fast variations) as well as a smaller value (**B**, rapid variations). The input-output correlation (between *μ*_ext_ and *r*_*N*_) is included as a reference (black dashed lines), and mean ± standard deviation of the population spike rate *r*_*N*_ are indicated (gray dashed lines, shaded areas). For each parametrization, activity time series of 60 s duration were generated (from 50,000 aEIF neurons and each derived model), from which the first second was omitted (each) to exclude transients, since the initial conditions of the models were not matched. Representative time series examples are shown on the right side with parameter values indicated (**C**), where empty and filled symbols correspond to large and small correlation time $\tau_{\text{ou}}^{\mu }$, respectively, relating the examples to the panels **A** and **B**. The adaptation current traces were excluded in all but the first example to allow for a larger number of parameter points.

We find that three of the four low-dimensional spike rate models (spec_2_, LN_exp_, LN_dos_) very well reproduce the spike rate *r*_*N*_ of the aEIF neurons: for the LN_exp_ model *ρ* > 0.95 and for the spec_2_ and LN_dos_ models *ρ* ≳ 0.8 (each) over the explored parameter space, see Fig 2. Only the basic spectral model (spec_1_) is substantially less accurate. Among the best models, the simplest (LN_exp_) overall outperforms spec_2_ and LN_dos_, in particular for fast and strong mean input variations. However, in the strongly mean-driven regime the best performing model is spec_2_.

We observe that the performance of any of the spike rate models decreases (with model-specific slope) with (i) increasing variation strength *ϑ*_*μ*_ larger than a certain (small) value, and with (ii) smaller $\tau_{\text{ou}}^{\mu }$, i.e., faster changes of *μ*_ext_. For small values of *ϑ*_*μ*_ fluctuations of *r*_*N*_, which are caused by the finite aEIF population size *N* and do not depend on the fluctuations of *μ*_ext_, deteriorate the performance measured by *ρ* (see also [21], p.13 right). This explains why *ρ* does not increase as *ϑ*_*μ*_ decreases (towards zero) for any of the models. Naturally, the FP model is the by far most accurate spike rate description in terms of both measures, correlation coefficient *ρ* and RMS distance. This is not surprising because the four low-dimensional models are derived from that (infinite-dimensional) representation. Thus, the performance of the FP system defines an upper bound on the correlation coefficient *ρ* and a lower bound on the RMS distance for the low-dimensional models.

In detail: for moderately fast changing mean input (large $\tau_{\text{ou}}^{\mu }$) the three models spec_2_, LN_exp_ and LN_dos_ exhibit excellent reproduction performance with *ρ* > 0.95, and spec_1_ shows correlation coefficients of at least *ρ* = 0.9 (Fig 2A), which is substantially better than *ρ*(*r*_*N*_, *μ*_ext_). The small differences between the three top models can be better assessed from the RMS distance measure. For large input variance $\sigma_{\text{ext}}^{2}$ the two LN models perform best (cf. Fig 2A, top, and for an example, 2C). For weak input variance and large mean (small *σ*_ext_, large $\bar{\mu }$) the spec_2_ model outperforms the LN models, unless the variation magnitude *ϑ*_*μ*_ is very large. For small mean $\bar{\mu }$, where transient activity is interleaved with periods of quiescence, the LN_exp_ model performs best, except for weak variations *ϑ*_*μ*_, where LN_dos_ is slightly better (see Fig 2A, bottom).

Stronger differences in performance emerge when considering faster changes of the mean input *μ*_ext_(*t*) (i.e., for small $\tau_{\text{ou}}^{\mu }$), see Fig 2B, and for examples, Fig 2C. The spec_1_ model again performs worst with *ρ* values even below the input/output correlation baseline *ρ*(*r*_*N*_, *μ*_ext_) for large mean input $\bar{\mu }$ (cf. Fig 2B, left). The spec_1_ spike rate typically decays too slowly (cf. Fig 2C). The three better performing models differ as follows: for large input variance and mean (large *σ*_ext_ and $\bar{\mu }$), where the spike rate response to the input is rather fast (cf. increased *ρ*(*r*_*N*_, *μ*_ext_)), the performance of all three models in terms of *ρ* is very high, but the RMS distance measure indicates that LN_exp_ is the most accurate model (cf. Fig 2B, top). For weak mean input LN_exp_ is once again the top model while LN_dos_ and, especially noticeable, spec_2_ show a performance decline (see example in Fig 2C). For weak input variance (Fig 2A, bottom), where significant (oscillatory) excursions of the spike rates in response to changes in the mean input can be observed (see also Fig 1), we obtain the following benchmark contrast: for large mean drive $\bar{\mu }$ the spec_2_ model performs best, except for large variation amplitudes *ϑ*_*μ*_, at which LN_exp_ is more accurate. Smaller mean input on the other hand corresponds to the most sensitive regime where periods of quiescence alternate with rapidly increasing and decaying spike rates. The LN_exp_ model shows the most robust and accurate spike rate reproduction in this setting, while LN_dos_ and spec_2_ each exhibit decreased correlation and larger RMS distances–spec_2_ even for moderate input variation intensities *ϑ*_*μ*_. The slowness approximation underlying the spec_2_ model likely induces an error due to the fast external input changes in comparison with the rather slow intrinsic time scale by the dominant eigenvalue, $\tau_{\text{ou}}^{\mu }=5\;\text{ms}$ vs. 1/|Re{λ_1_}| ≈ 15 ms (cf. visualization of the spectrum in Sect. *Spectral models*). Note that for these weak inputs the distribution of the spike rate is rather asymmetric (cf. Fig 2B). Interestingly the LN_dos_ model performs worse than LN_exp_ for large mean input variations (i.e., large *ϑ*_*μ*_) in general, and only slightly better for small input variance and mean input variations that are not too large and fast.

We would like to note that decreasing the Gaussian filter width *σ*_*t*_ to smaller values, e.g., fractions of a millisecond, can lead to a strong performance decline for the spec_2_ model because of its explicit dependence on first and second order time derivatives of the mean input.

**Fig 3:**
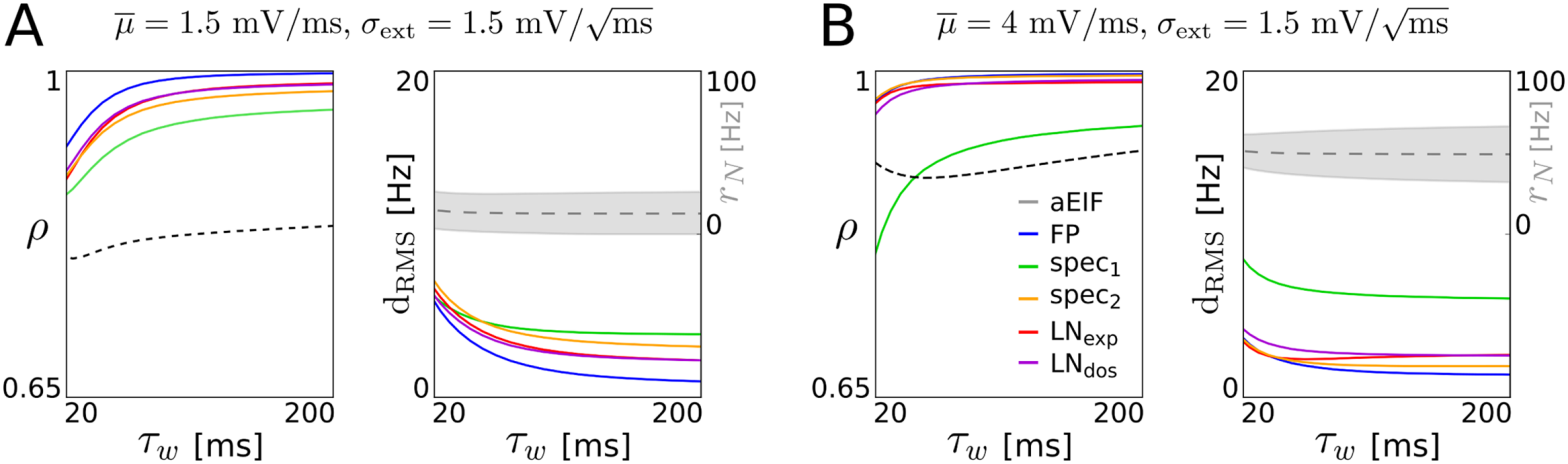
Effect of adaptation current timescale on reproduction accuracy. Performance measures and population spike rate statistics (cf. Fig 2A and 2B) as a function of the adaptation time constant *τ*_*w*_, that takes values between 20 ms (equal to the membrane time constant) and 200 ms (used throughout the rest of the study). The spike-triggered adaptation increment *b* was co-varied (antiproportional to *τ*_*w*_) such that the product *τ*_*w*_ *b* = 8 pAs is fixed for all shown parametrizations. The input mean *μ*_ext_(*t*) fluctuates with timescale $\tau_{\text{ou}}^{\mu }=50\;\text{ms}$ and strength *ϑ*_*μ*_ = 0.54 mV/ms (same value as for examples in Fig 2C) around a smaller (**A**) and a larger (**B**) baseline mean $\bar{\mu }$, while the input deviation *σ*_ext_ is constant. Note that the rightmost parametrization of **A** corresponds to Fig 2C (top example) and is contained in Fig 2A (bottom right) while that of **B** is shown in Fig 2A (bottom left).

Furthermore, we show how the adaptation parameters affect the reproduction performance of the different models in Fig 3. The adaptation time constant *τ*_*w*_ and spike-triggered adaptation increment *b* are varied simultaneously (keeping their product constant) such that the average spike rate and adaptation current, and thus the spiking regime, remain comparable for all parametrizations. As expected, the accuracy of the derived models decreases for faster adaptation current dynamics, due to the adiabatic approximation that relies on sufficiently slow adaptation (cf. Sect. Methods). Interestingly however, the performance of all reduced models (except spec_1_) declines only slightly as the adaptation time constant decreases to the value of the membrane time constant (which means the assumption of separated time scales underlying the adiabatic approximation is clearly violated). This kind of robustness is particularly pronounced for input with large baseline mean $\bar{\mu }$ and small noise amplitude *σ*_ext_, cf. Fig 3B.

### Performance for variations of the input variance

For perfectly balanced excitatory and inhibitory synaptic coupling the contribution of presynaptic activity to the mean input *μ*_syn_ is zero by definition, but the input variance $\sigma_{\text{syn}}^{2}$ is always positively (linearly) affected by a presynaptic spike rate–even for a negative synaptic efficacy *J* (cf. Eq (2)). To assess the performance of the derived models in this scenario, but within the reference setting of an uncoupled population, we consider constant external mean drive *μ*_ext_ and let the variance $\sigma_{\text{ext}}^{2}\left(t\right)$ evolve according to a filtered OU process (such as that used for the mean input *μ*_ext_ in the previous section) with parameters $\bar{\sigma^{2}}$ and *ϑ*_*σ*^2^_ of the stationary normal distribution $N(\bar{\sigma^{2}},\vartheta_{\sigma^{2}}^{2})$, correlation time $\tau_{\text{ou}}^{\sigma^{2}}$ and Gaussian filter standard deviation *σ*_*t*_ as before.

**Fig 4:**
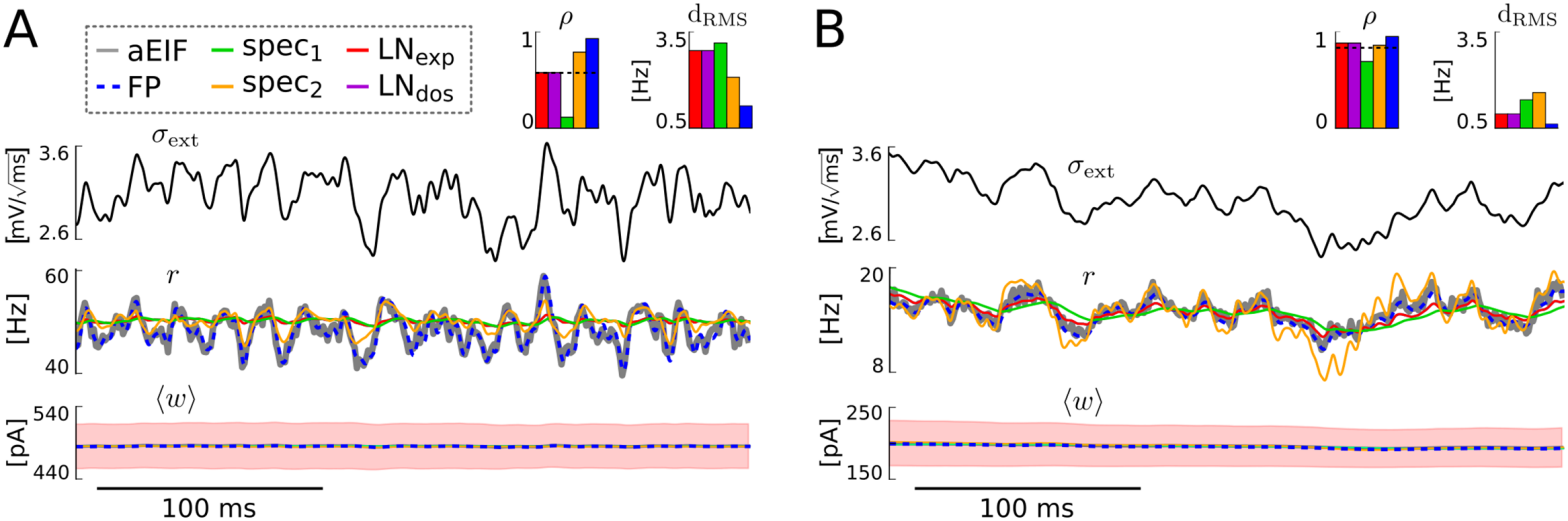
Performance for variations of the input variance. Time series of population spike rate and mean adaptation current from the different models in response to varying $\sigma_{\text{ext}}^{2}$ for large mean input and rapid variations, *μ*_ext_ = 4 mV/ms, $\tau_{\text{ou}}^{\sigma^{2}}=5\;\text{ms}$ (**A**) and for small mean input and moderately fast variations, *μ*_ext_ = 1.5 mV/ms, $\tau_{\text{ou}}^{\sigma^{2}}=50\;\text{ms}$ (**B**). The values for the remaining input parameters were ${\bar{\sigma }}_{\text{ext}}^{2}=9\;{\text{mV}}^{2}/\text{ms}$, *ϑ*_*σ*^2^_ = 2 mV^2^/ms. For the aEIF population 〈*w*〉± standard deviation are visualized (red shaded areas). Note that the mean adaptation time series of all models as well as the spike rates of the cascade based models are on top of each other. The indicated Pearson correlation coefficients (with dashed input-output correlation) and root mean square distances were calculated from simulated spike rate time series of 60 s duration from which the first second was excluded, as the initial conditions of the models were not matched. In **A** the correlation *ρ* (but not the distance d_RMS_) between the spike rates of the model spec_1_ and the aEIF population) is strongly decreased due to a small time lag between the two time series which is difficult to see in the figure.

The results of two input parametrizations are shown in Fig 4. For large input mean *μ*_ext_ and rapidly varying variance $\sigma_{\text{ext}}^{2}\left(t\right)$ the spike rate response of the aEIF population is very well reproduced by the FP model and, to a large extent, by the spec_2_ model (cf. Fig 4A). This may be attributed to the fact that the latter model depends on the first two time derivatives of the input variance $\sigma_{\text{ext}}^{2}$. The LN models cannot well reproduce the rapid spike rate excursions in this setting, and the spec_1_ model performs worst, exhibiting time-lagged spike rate dynamics compared to *r*_*N*_(*t*) which leads to a very small value of correlation coefficient *ρ* (below the input/output correlation baseline $\rho (r_{N},\sigma_{\text{ext}}^{2})$). For smaller mean input *μ*_ext_ and moderately fast varying variance $\sigma_{\text{ext}}^{2}\left(t\right)$ (larger correlation time $\tau_{\text{ou}}^{\sigma^{2}}$) the fluctuating aEIF population spike rate is again nicely reproduced by the FP model while the rate response of the spec_2_ model exhibits over-sensitive behavior to changes in the input variance, as indicated by the large RMS distance (see Fig 4B). This effect is even stronger for faster variations, i.e., smaller $\tau_{\text{ou}}^{\sigma^{2}}$ (cf. supplementary visualization S1 Fig). The LN models perform better in this setting, and the spec_1_ model (again) performs worst in terms of correlation coefficient *ρ* due to its time-lagged spike rate response.

It should be noted that the lowest possible value of the input standard deviation, i.e., *σ*_ext_ (plus a nonnegative number in case of recurrent input) cannot be chosen completely freely but must be large enough ($≳\;0.5\;\text{mV}/\sqrt{\text{ms}}$) for our parametrization. This is due to theoretical reasons (Fokker-Planck formalism) and practical reasons (numerics for Fokker-Planck solution and for calculation of the derived quantities, such as *r*_∞_).

### Oscillations in a recurrent network

To demonstrate the applicability of the low-dimensional models for network analyses we consider a recurrently coupled population of aEIF neurons that produces self-sustained network oscillations by the interplay of strong excitatory feedback and spike-triggered adaptation or, alternatively, by delayed recurrent synaptic inhibition [16, 23]. The former oscillation type is quite sensitive to changes in input, adaptation and especially coupling parameters for the current-based type of synaptic coupling considered here and due to lack of (synaptic) inhibition and refractoriness. For example, a small increase in coupling strength can lead to a dramatic (unphysiologic) increase in oscillation amplitude because of strong recurrent excitation. Hence we consider a difficult setting here to evaluate the reduced spike rate models–in particular, when the network operates close to a bifurcation.

**Fig 5:**
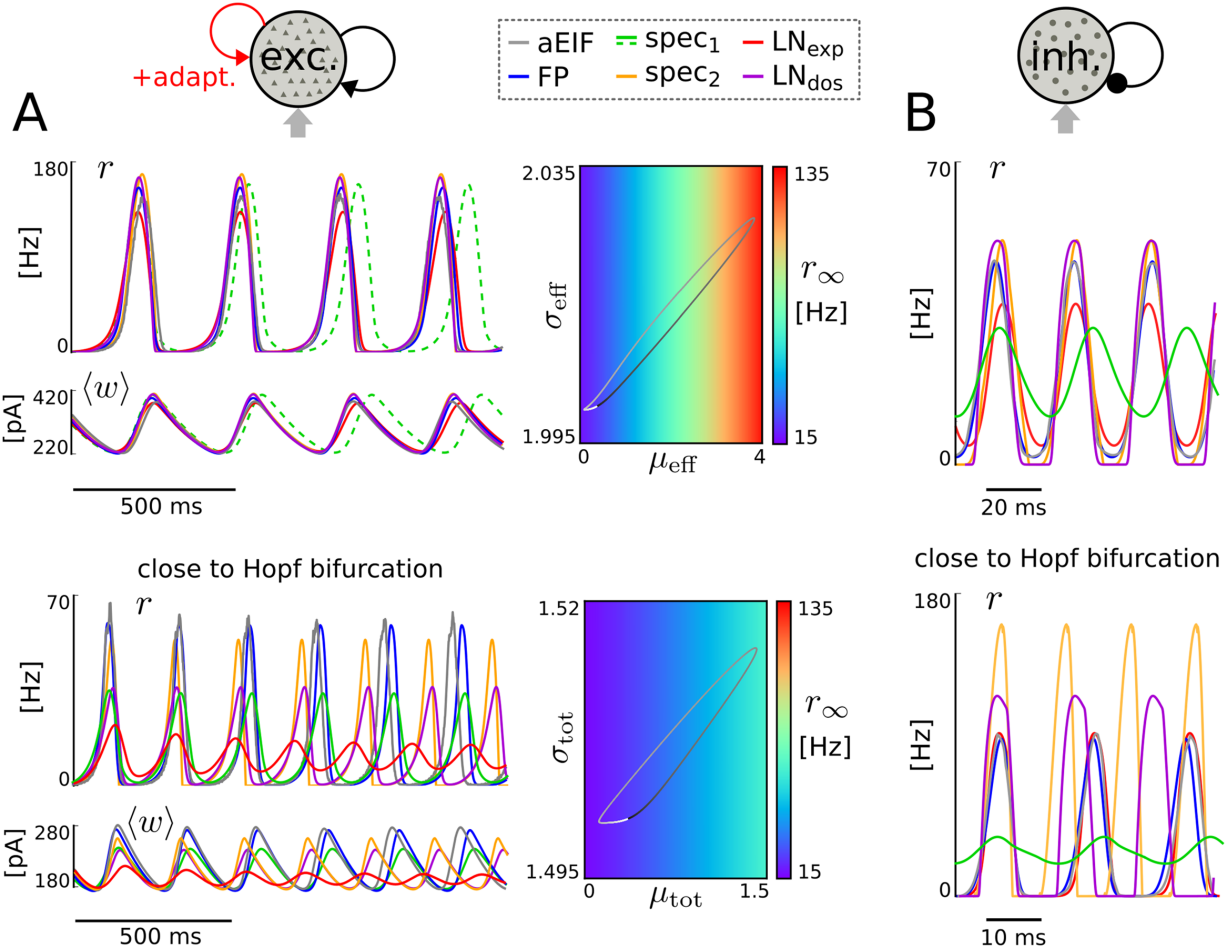
Network-generated oscillations. Oscillatory population spike rate and mean adaptation current of 50,000 excitatory coupled aEIF neurons and each of the derived models (for constant external input moments) generated by the interplay of recurrent excitation/adaptation current (**A**) and by delayed recurrent inhibition (**B**). In addition, the limit cycle of the LN_exp_ model is shown in terms of the (quantity) steady-state spike rate *r*_∞_ as a function of effective input moments *μ*_eff_, $\sigma_{\text{eff}}^{2}$ (**A**, top) and for the spec_2_ model in dependence of the total input moments (*μ*_tot_, $\sigma_{\text{tot}}^{2}$ (**A**, bottom). The phase of the cycle is visualized by grayscale color code (increasing phase from black to white). The values for the input, adaptation and coupling parameters were *μ*_ext_ = 1.5 mV/ms, $\sigma_{\text{ext}}=2\;\text{mV}/\sqrt{\text{ms}}$, *a* = 3 nS, *b* = 30 pA (**A**, top), *μ*_ext_ = 1.275 mV/ms, $\sigma_{\text{ext}}=1.5\;\text{mV}/\sqrt{\text{ms}}$, *a* = 3 nS, *b* = 60 pA (**A**, bottom), *K* = 1000, *J* = 0.03 mV, *τ*_*d*_ = 3 ms (**A**, both). In **B** adaptation was removed (*a* = *b* = 0) and delays were identical *d*_*ij*_ = *d*; input and coupling parameter values were *μ*_ext_ = 1.5 mV/ms, $\sigma_{\text{ext}}=1.5\;\text{mV}/\sqrt{\text{ms}}$, *K* = 1000, *J* = −0.0357 mV, *d* = 10 ms (top) and *μ*_ext_ = 3 mV/ms, $\sigma_{\text{ext}}=2\;\text{mV}/\sqrt{\text{ms}}$, *K* = 1000, *J* = −0.087 mV, *d* = 5 ms (bottom).

In Fig 5A we present two example parametrizations from a region (in parameter space) that is characterized by stable oscillations. This means the network exhibits oscillatory spike rate dynamics for constant external input moments *μ*_ext_ and $\sigma_{\text{ext}}^{2}$. The derived models reproduce the limit cycle behavior of the aEIF network surprisingly well, except for small frequency and amplitude deviations (FP, spec_2_, LN_dos_, LN_exp_) and larger frequency mismatch (spec_1_), see Fig 5A, top. For weaker input moments and increased spike-triggered adaptation strength the network is closer to a Hopf bifurcation [16, 23]. It is, therefore, not surprising that the differences in oscillation period and amplitude are more prominent (cf. Fig 5A, bottom). The bifurcation point of the LN_exp_ model is slightly shifted, shown by the slowly damped oscillatory convergence to a fixed point. This suggests that the bifurcation parameter value of each of the derived models is not far from the true critical parameter value of the aEIF network but can quantitatively differ (slightly) in a model-dependent way.

The second type of oscillation is generated by delayed synaptic inhibition [22] and does not depend on the (neuronal) inhibition that is provided by an adaptation current. To demonstrate this independence the adaptation current was disabled (by setting the parameters *a* = *b* = 0) for the two respective examples that are shown in Fig 5B. Similarly as for the previous oscillation type, the low-dimensional models (except spec_1_) reproduce the spike rate limit cycle of the aEIF network surprisingly well, in particular for weak external input (see Fig 5B, top). For larger external input and stronger inhibition with shorter delay the network operates close to a Hopf bifurcation, leading to larger differences in oscillation amplitude and frequency in a model-dependent way (Fig 5B, bottom). Note that the intermediate (Fokker-Planck) model very well reproduces the inhibition-based type of oscillation which demonstrates the applicability of the underlying mean-field approximation. We would also like to note that enabling the adaptation current dynamics (only) leads to decreased average spike rates but does not affect the reproduction accuracy.

We would like to emphasize that the previous comprehensive evaluations for an uncoupled population provide a deeper insight on the reproduction performance–also for a recurrent network–than the four examples shown here, as explained in the Sect. *Performance for variations of the mean input*. For example, the (improved) reproduction performance for increased input variance in the uncoupled setting (cf. Fig 2) informs about the reproduction performance for networks of excitatory and inhibitory neurons that are roughly balanced, i.e., where the overall input mean is rather small compared to the input standard deviation.

### Implementation and computational complexity

We have developed efficient implementations of the derived models using the Python programming language and by employing the library Numba for low-level machine acceleration [24]. These include: (i) the numerical integration of the Fokker-Planck model using an accurate finite volume scheme with implicit time discretization (cf. Sect. Methods), (ii) the parallelized precalculation of the quantities required by the low-dimensional spike rate models and (iii) the time integration of the latter models, as well as example scripts demonstrating (i)–(iii). The code is available as open source software under a free license at GitHub: https://github.com/neuromethods/fokker-planck-based-spike-rate-models

With regards to computational cost, summarizing the results of several aEIF network parametrizations, the duration to generate population activity time series for the low-dimensional spike rate models is usually several orders of magnitude smaller compared to numerical simulation of the original aEIF network and a few orders of magnitude smaller in comparison to the numerical solution of the FP model. For example, considering a population of 50,000 coupled neurons with 2% connection probability, a single simulation run of 5 s and the same integration time step across the models, the computation times amounted to 1.1–3.6 s for the low-dimensional models (with order–fast to slow–LN_exp_, spec_1_, LN_dos_, spec_2_), about 100 s for the FP model and roughly 1500 s for the aEIF network simulation on a dual-core laptop computer. The time difference to the network simulation substantially increases with the numbers of neurons and connections, and with spiking activity within the network due to the extensive propagation of synaptic events. Note that the speedup becomes even more pronounced with increasing number of populations, where the runtimes of the FP model and the aEIF network simulation scale linearly and the low-dimensional models show a sublinear runtime increase due to vectorization of the state variables representing the different populations.

The derived low-dimensional (ODE) spike rate models are very efficient to integrate given that the required input-dependent parameters are available as precalulated look-up quantities. For the grids used in this contribution, the precomputation time was 40 min. for the cascade (LN_exp_, LN_dos_) models and 120 min. for the spectral (spec_1_, spec_2_) models, both on a hexa-core desktop computer. The longer calculation time for the spectral models was due to the finer internal grid for the mean input (see S1 Text).

Note that while the time integration of the spec_2_ model is on the same order as for the other low-dimensional models its implementation complexity is larger because of the many quantities it depends on, cf. Eqs (63)–(66).

## Discussion

In this contribution we have developed four low-dimensional models that approximate the spike rate dynamics of coupled aEIF neurons and retain all parameters of the underlying model neurons. These simple spike rate models were derived in two different ways from a Fokker-Planck PDE that describes the evolving membrane voltage distribution in the mean-field limit of large networks, and is complemented by an ODE for the population-averaged slow adaptation current. Two of the reduced spike rate models (spec_1_ and spec_2_) were obtained by a truncated spectral decomposition of the Fokker-Planck operator assuming vanishingly slow (for spec_1_) or moderately slow (for spec_2_) changes of the input moments. The other two reduced models (LN_exp_ and LN_dos_) are described by a cascade of linear filters (one for the input mean and another for its standard deviation) and a nonlinearity which were derived from the Fokker-Planck equation, and subsequently the filters were semi-analytically approximated. Our approaches build upon [18–20] as well as [21], and extend those methods for adaptive nonlinear integrate-and-fire neurons that are sparsely coupled with distributed delays (cf. Sect. Methods).

We have compared the different spike rate representations for a range of biologically plausible input statistics and found that three of the reduced models (spec_2_, LN_exp_ and LN_dos_) accurately reproduce the spiking activity of the underlying aEIF population while one model (spec_1_) shows the least accuracy. Among the best models, the simplest (LN_exp_) was the most robust and (somewhat surprisingly) overall outperformed spec_2_ and LN_dos_–especially in the sensitive regime of rapidly changing sub- and suprathreshold mean drive and in general for rapid and strong input variations. The LN_exp_ model did not exhibit exaggerated deflections in that regime as compared to the other two models. This result is likely due to the importance of the quantitatively correct decay time of the filter for the mean input in the LN_exp_ model, while the violations of the slowness assumptions for the spec_2_ and LN_dos_ models seem more harmful in this regime. In the strongly mean-driven regime, however, the best performing model was spec_2_ for variations both in the mean drive (as long as those variations are not too strong and fast) and for variations of the input variance.

We have also demonstrated that the low-dimensional models well reproduce the dynamics of recurrently coupled aEIF populations in terms of asynchronous states (see Fig 1) and spike rate oscillations (cf. Fig 5), where mild deviations at critical (bifurcation) parameter values are expected due to the approximative nature of the model reduction.

The computational demands of the low-dimensional models are very modest in comparison to the aEIF network and also to the integration of the Fokker-Planck PDE, for which we have developed a novel finite volume discretization scheme. We would like to emphasize that any change of a parameter value for input, coupling or adaptation current does not require renewed precomputations. To facilitate the application of the presented models we have made available implementations that precompute all required quantities and numerically integrate the derived low-dimensional spike rate models as well as the Fokker-Planck equation, together with example (Python) scripts, as open source software.

Since the derived models are formulated in terms of simple ODEs, they allow to conveniently perform linear stability analyses, e.g., based on the eigenvalues of the Jacobian matrix of the respective vector field. In this way network states can be rapidly characterized by quantifying the bifurcation structure of the population dynamics–including regions of the parameter space where multiple fixed points and/or limit cycle attractors co-exist. For a characterization of stable network states by numerical continuation and an assessment of their controllability through neuromodulators using the LN_exp_ model see [23] ch. 4.2 and [25]. Furthermore, the low-dimensional models are well suited to be employed in large neuronal networks of multiple populations for efficient simulations of population-averaged activity time series. Overall, the LN_exp_ model seems a good candidate for that purpose considering its accuracy and robustness, as well as its computational and implementational simplicity.

### Extensions

#### Heterogeneity

We considered a homogeneous population of neurons in the sense that the parameter values across model neurons are identical except those for synaptic input. Thereby we assume that neurons with similar dynamical properties can be grouped into populations [3]. Heterogeneity is incorporated by distributed synaptic delays, by sparse random coupling, and by fluctuating external inputs for each neuron. The (reduced) population models further allow for heterogeneous synaptic strengths that are sampled from a Gaussian distribution and can be included in a straightforward way [16, 26] (see also Sect. Methods). Distributed values for other parameters (of the isolated model neurons within the same population) are currently not supported.

#### Multiple populations

The presented mean-field network model can be easily adjusted for multiple populations. In this case we obtain a low-dimensional ODE for each population and the overall synaptic moments for population *k* become

$$\mu_{\text{syn},k}=\mu_{\text{ext},k}\left(t\right)+\sum_{l}\;J_{kl}K_{kl}r_{d,kl}\left(t\right),\;\sigma_{\text{syn},k}^{2}=\sigma_{\text{ext},k}^{2}\left(t\right)+\sum_{l}\;J_{kl}^{2}K_{kl}r_{d,kl}\left(t\right),$$ (13)

where *J*_*kl*_ is the synaptic strength for the *K*_*kl*_ neurons from population *l* targeting neurons from population *k* and *r*_*d*,*kl*_ is the delayed spike rate of population *l* affecting population *k* (cf. Eq (2)). For each pair of coupled populations we may consider identical or distributed delays (using distributions from the exponential family) as well as identical or distributed synaptic strengths (sampled from a Gaussian distribution).

#### Synaptic coupling

Here we described synaptic interaction by delayed (delta) current pulses with delays sampled from an exponential distribution. This description leads to a fluctuating overall synaptic input current with white noise characteristics. Interestingly, for the mean-field dynamics this setting is very similar to considering exponentially decaying synaptic currents with a decay constant that matches that of the delay distribution, although the overall synaptic input current is a colored noise process in that case, see [27] and, for an intuitive explanation [28].

A conductance-based model of synaptic coupling can also be considered in principle [16, 29], which results in a multiplicative noise process for the overall synaptic input. This, however, would in general impede the beneficial concept of precalculated “look-up” quantities that are unaffected by the input and coupling parameters.

It should be noted that most current- or conductance-based models of synaptic coupling (including the one considered here) can produce unphysiologically large amounts of synaptic current in case of high presynaptic activity, unless the coupling parameters are carefully tuned. This problem can be solved, for example, by considering a (more realistic) model of synaptic coupling based on [30], from which activity-dependent coupling terms can be derived for the mean-field and reduced population models [23] ch. 4.2. Using that description ensures robust simulation of population activity time series without having to fine-tune the coupling parameter values, which is particularly useful for multi-population network models. In this contribution though we used for simplicity a basic synaptic coupling model that has frequently been applied in the mean-field literature.

#### Input noise process

The Gaussian stochastic process driving the individual neurons could also be substituted by colored noise, which would lead to a Fokker-Planck model with increased dimensionality [31]. However, this would require more complex and computationally expensive numerical schemes not only to solve that model but also for the different dimension reduction approaches.

#### Slow adaptation

To derive low-dimensional models of population activity we approximated the adaptation current by its population average, justified by its slow dynamics compared to the other time scales of the system. This approximation is equivalent to a first order moment closure method [17]. In case of a faster adaptation time scale the approximation may be improved by considering second and higher order moments [17, 32].

#### Population size

The mean-field models presented here can well reproduce the dynamics of population-averaged state variables (that is, spike rate, mean membrane voltage, and mean adaptation current) for large populations (*N* → ∞ in the derivation). Fluctuations of those average variables due to the finite size of neuronal populations, however, are not captured. Hence, it would be interesting to extend the mean-field models so as to reproduce these (so-called) finite size effects, for example, by incorporating an appropriate stochastic process [18] or using concepts from [15].

#### Cascade approach

For uncoupled EIF populations (without an adaptation current) and constant input standard deviation it has been shown that the LN cascade approximation performs well for physiological ranges of amplitude and time scale for mean input variations [21]. Our results for the cascade models are consistent with [21], but the performance is substantially improved for the sensitive low (baseline) input regime (LN_exp_ and LN_dos_, also in absence of adaptation), and damped oscillatory behavior (including over- and undershoots) is accounted for by the LN_dos_ model.

To achieve these improvements we semi-analytically fit the linear filters derived from the Fokker-Planck equation using exponential and damped oscillator functions considering a range of input frequencies. The approximation can be further improved by using more complex functions, such as a damped oscillator with two time scales. That, however, can lead to less robustness (i.e., undesired model behavior) for rapid and strong changes of the input moments (cf. Sect. Methods).

LN cascade models are frequently applied in neuroscience to describe population activity, and the model components are often determined from electrophysiological recordings using established techniques. The methodology presented here contributes to establishing quantitative links between networks of spiking neurons, a mesoscopic description of population activity and recordings at the population level.

#### Spectral approach

Here we provide a new numerical solver for the eigenvalue problem of the Fokker-Planck operator and its adjoint. This allows to compute the full spectrum together with associated eigenfunctions and is applicable to nonlinear integrate-and-fire models, extending [18, 19, 33].

Using that solver the spec_2_ model, which is based on two eigenvalues, can be further improved by interpolating its coefficients, Eqs (63)–(66), around the double eigenvalues at the spectrum’s real-to-complex transition. This interpolation would effectively smooth the quantities—e.g., preventing the jumps and kinks that are present in the visualization of Sect. *Spectral models*—and is expected to increase the spike rate reproduction accuracy (particularly for weak mean input) beyond what was reported in this contribution.

The spec_2_ model can also be extended to yield a third order ODE with everywhere smooth coefficients by considering an additional eigenvalue (cf. Sect. *Remarks on the spectrum*).

Moreover, the spec_2_ model, and more generally the whole spectral decomposition approach, can be extended to account for a refractory period in the presence of time-varying total input moments, e.g., by building upon previous attempts [18, 34, 35].

Furthermore, it could be beneficial to explicitly quantify the approximation error due to the slowness assumption that underlies the spec_2_ model by integration of the (original) spectral representation of the Fokker-Planck model.

Both reduced spectral models allow for a refined description of the mean adaptation current dynamics, cf. Eq (1), by replacing the mean membrane voltage 〈*V*〉 with its steady-state value 〈*V*〉_∞_, using that the membrane voltage distribution is available through the eigenfunctions of the Fokker-Planck operator.

The numerical eigenvalue solver can be extended in a straightforward way to yield quantities that are required by the original spectral representation of the Fokker-Planck model and by the corresponding stochastic equation for finite population size [18].

### Alternative derived models

In addition to the work we build upon [18–21] (cf. Sect. Methods) there are a few other approaches to derive spike rate models from populations of spiking neurons. Some methods also result in an ODE system, taking into account (slow) neuronal adaptation [17, 26, 36–38] or disregarding it [39]. The settings differ from the work presented here in that (i) the intrinsic neuronal dynamics are adiabatically neglected [17, 26, 36, 37], (ii) only uncoupled populations [38] or all-to-all connected networks [17, 36, 39] are assumed in contrast to sparse connectivity, and (iii) (fixed) heterogeneous instead of fluctuating input is considered [39]. Notably, these previous methods yield rather qualitative agreements with the underlying spiking neuron population activity except for [39] where an excellent quantitative reproduction for (non-adaptive) quadratic integrate-and-fire oscillators with quenched input randomness is reported.

Other approaches yield mesoscopic representations of population activity in terms of model classes that are substantially less efficient to simulate and more complicated to analyze than low-dimensional ODEs [14–17, 40–42]. The spike rate dynamics in these models has been described (i) by a rather complex ODE system that depends on a stochastic jump process derived for integrate-and-fire neurons without adaptation [40], (ii) by PDEs for recurrently connected aEIF [16] or Izhikevich [17] neurons, (iii) by an integro-PDE with displacement for non-adaptive neurons [42] or (iv) by integral equations that represent the (mean) activity of coupled phenomenological spiking neurons without [41] and with adaptation [14, 15].

Furthermore, the stationary condition of a noise-driven population of adaptive EIF neurons [32, 43, 44] and the first order spike rate response to weak input modulations [43, 44] have been analyzed using the Fokker-Planck equation. Ref. [32] also considered a refined approximation of the (purely spike-triggered) adaptation current including higher order moments.

It may be interesting for future studies to explore ways to extend the presented methods and relax some of the underlying assumptions, in particular, considering (i) the diffusion approximation (via shot noise input, e.g., [45, 46]), (ii) the Poisson assumption (e.g., using the concept from [47] in combination with results from [48]) and (iii) (noise) correlations (see, e.g., [49]).

## Methods

Here we present all models in detail—the aEIF network (*ground truth*), the mean-field FP system (*intermediate model*) and the low-dimensional models: spec_1_, spec_2_, LN_exp_, LN_dos_—including step-by-step derivations and essential information on the respective numerical solution methods. An implementation of these models using Python is made available at GitHub: https://github.com/neuromethods/fokker-planck-based-spike-rate-models

### Network model

We consider a large (homogeneous) population of *N* synaptically coupled aEIF model neurons [5]. Specifically, for each neuron (*i* = 1, …, *N*), the dynamics of the membrane voltage *V*_*i*_ is described by

$$C\frac{dV_{i}}{dt}=I_{L}\left(V_{i}\right)+I_{\text{exp}}\left(V_{i}\right)-w_{i}+I_{\text{syn},i}\left(t\right),$$ (14)

where the capacitive current through the membrane with capacitance *C* equals the sum of three ionic currents and the synaptic current *I*_syn,*i*_. The ionic currents consist of a linear leak current *I*_L_(*V*_*i*_) = −*g*_L_(*V*_*i*_ − *E*_L_) with conductance *g*_L_ and reversal potential *E*_L_, a nonlinear term *I*_exp_(*V*_*i*_) = *g*_L_ Δ_T_ exp((*V*_*i*_ − *V*_T_)/Δ_T_) that approximates the rapidly increasing Na^+^ current at spike initiation with threshold slope factor Δ_T_ and effective threshold voltage *V*_T_, and the adaptation current *w*_*i*_ which reflects a slowly deactivating K^+^ current. The adaptation current evolves according to

$$\tau_{w}\frac{dw_{i}}{dt}=a\left(V_{i}-E_{w}\right)-w_{i},$$ (15)

with adaptation time constant *τ*_*w*_. Its strength depends on the subthreshold membrane voltage via conductance *a*. *E*_*w*_ denotes its reversal potential. When *V*_*i*_ increases beyond *V*_T_, it diverges to infinity in finite time due to the exponentially increasing current *I*_exp_(*V*_*i*_), which defines a spike. In practice, however, the spike is said to occur when *V*_*i*_ reaches a given value *V*_s_—the spike voltage. The downswing of the spike is not explicitly modeled; instead, when *V*_*i*_ ≥ *V*_s_, the membrane voltage *V*_*i*_ is instantaneously reset to a lower value *V*_r_. At the same time, the adaptation current *w*_*i*_ is incremented by a value of parameter *b*, which implements suprathreshold (spike-dependent) activation of the adaptation current.

Immediately after the reset, *V*_*i*_ and *w*_*i*_ are clamped (i.e., remain constant) for a short refractory period *T*_ref_, and subsequently governed again by Eqs (14) and (15). At the end of the Methods section we describe how (optionally) a spike shape can be included in the aEIF model, together with the associated small changes for the models derived from it.

To complete the network model the synaptic current in Eq (14) needs to be specified: for each cell it is given by the sum of recurrent and external input, *I*_syn,*i*_ = *I*_rec,*i*_(*t*) + *I*_ext,*i*_(*t*). Recurrent synaptic input is received from *K* other neurons of the network, that are connected in a sparse (*K* ≪ *N*) and uniformly random way, and is modeled by

$$I_{\text{rec},i}=C\;\sum_{j}\;J_{ij}\;\sum_{t_{j}}\;\delta (t-t_{j}-d_{ij}),$$ (16)

where *δ* denotes the Dirac delta function. Every spike by one of the *K* presynaptic neurons with indices *j* and spike times *t*_*j*_ causes a postsynaptic membrane voltage jump of size *J*_*ij*_. The coupling strength is positive (negative) for excitation (inhibition) and of small magnitude. Here it is chosen to be constant, i.e., *J*_*ij*_ = *J*. Each of these membrane voltage deflections occur after a time delay *d*_*ij*_ that takes into account (axonal and dendritic) spike propagation times and is sampled (independently) from a probability distribution *p*_*d*_. In this work we use exponentially distributed delays, i.e., *p*_*d*_(*τ*) = exp(−*τ*/*τ*_*d*_)/*τ*_*d*_ (for *τ* ≥ 0) with mean delay *τ*_*d*_.

The second type of synaptic input is a fluctuating current generated from network-external neurons,

$$I_{\text{ext},i}=C\left[\mu_{\text{ext}}\left(t\right)+\sigma_{\text{ext}}\left(t\right)\xi_{\text{ext},i}\left(t\right)\right],$$ (17)

with time-varying moments *μ*_ext_ and $\sigma_{\text{ext}}^{2}$, and unit Gaussian white noise process *ξ*_ext,*i*_. The latter is uncorrelated with that of other neurons *j* ≠ *i*, i.e., 〈*ξ*_ext,*i*_(*t*)*ξ*_ext,*j*_(*t* + *τ*)〉 = *δ*(*τ*)*δ*_*ij*_, where 〈·〉 denotes expectation (w.r.t. the joint ensemble of noise realizations at times *t* and *t* + τ) and *δ*_*ij*_ is the Kronecker delta. This external current, for example, accurately approximates the input generated from a large number of independent Poisson neurons that produce instantaneous postsynaptic potentials of small magnitude, cf. [48].

The spike rate *r*_*N*_ of the network is defined as the population-averaged number of emitted spikes per time interval [*t*, *t* + Δ*T*],

$$r_{N}(t)=\frac{1}{N}\;\sum_{i=1}^{N}\;\frac{1}{\Delta T}\;\int_{t}^{t+\Delta T}\;\sum_{t_{i}}\;\delta (s-t_{i})ds,$$ (18)

where the interval size Δ*T* is practically chosen small enough to capture the dynamical structure and large enough to yield a comparably smooth time evolution for a finite network, i.e., *N* < ∞.

We chose values for the neuron model parameters to describe cortical pyramidal cells, which exhibit “regular spiking” behavior and spike frequency adaptation [7, 50, 51]. For the complete parameter specification see Table 1.

**Table 1:** Parameter values used throughout the study.

Name	Symbol	Value
**Network model**		
Number of neurons	*N*	50,000
Membrane capacitance	*C*	200 pF
Leak conductance	*g*_L_	10 nS
Leak reversal potential	*E*_L_	−65 mV
Threshold slope factor	Δ_T_	1.5 mV
Threshold voltage	*V*_T_	−50 mV
Spike voltage	*V*_s_	−40 mV
Reset voltage	*V*_r_	−70 mV
Subthreshold adaptation conductance^1^	*a*	4 nS
Spike-triggered adaptation increment^1^	*b*	40 pA
Adaptation reversal potential	*E*_*w*_	−80 mV
Adaptation time constant	*τ*_*w*_	200 ms
Refractory period^2^	*T*_ref_	0 ms
Gaussian filter width for external input	*σ*_*t*_	1 ms
Discretization time step	Δ*t*	0.05 ms
Spike rate estimation bin width	Δ*T*	1 ms
**Fokker-Planck model**		
Membrane voltage lower bound	*V*_lb_	−200 mV
Finite-volume membrane voltage spacing	Δ*V*	0.028 mV
Discretization time step	Δ*t*	0.05 ms
**Low-dimensional models**		
Discretization time step	Δ*t*	0.01 ms
Membrane voltage spacing^3^	Δ*V*	0.01 mV
Spacing of mean input^3^	Δ*μ*	0.025 mV/ms
Spacing of input standard deviation^3^	Δ*σ*	$0.1\;\text{mV}/\sqrt{\text{ms}}$

^1^If not specified otherwise.

^2^A nonzero refractory period is not supported by the spec_2_ model.

^3^Parameters for precalculation of model quantities (before simulation).

The values of coupling parameters (*K*, *J*, *τ*_*d*_) are specified in the captions of Figs 1 and 5, the values of parameters for the external input, *μ*_ext_ or ($\bar{\mu }$, $\tau_{\text{ou}}^{\mu }$, *ϑ*_*μ*_), and *σ*_ext_ or ($\bar{\sigma^{2}}$, $\tau_{\text{ou}}^{\sigma^{2}}$, *ϑ*_*σ*^2^_), are provided in each figure (caption).

All network simulations were performed using the Python software BRIAN2 [52, 53] with C++ code generation enabled for efficiency. The aEIF model Eqs (14) and (15) were discretized using the Euler-Maruyama method with equidistant time step Δ*t* and initialized with *w*_*i*_(0) = 0 and *V*_*i*_(0) that is (independently) sampled from a Gaussian initial distribution *p*_0_(*V*) with mean *V*_r_ − *δV* and standard deviation *δV*/2 where *δV* = *V*_T_ − *V*_r_. Note that the models derived in the following Sects. do not depend on this particular initial density shape but allow for an arbitrary (density) function *p*_0_.

### Fokker-Planck system

#### Adiabatic approximation

The time scales of (slow) K^+^ channel kinetics which are effectively described by the adaptation current *w*_*i*_, cf. Eq (15), are typically much larger than the faster membrane voltage dynamics modeled by Eq (14), i.e., *τ*_*w*_ ≫ *C*/*g*_L_ [54–57]. This observation justifies to replace the individual adaptation current *w*_*i*_ in Eq (14) by its average across the population, ${\langle w\rangle }_{N}=1/N\;\sum_{i=1}^{N}w_{i}\left(t\right)$, in order to reduce computational demands and enable further analysis. The mean adaptation current is then governed by [16, 26, 48, 58]

$$\frac{d{\left\langle w\right\rangle }_{N}}{dt}=\frac{a({\left\langle V\right\rangle }_{N}-E_{w})-{\left\langle w\right\rangle }_{N}}{\tau_{w}}+b\;r_{N}\left(t\right),$$ (19)

where 〈*V*〉_*N*_ denotes the time-varying population average of the membrane voltage of non-refractory neurons.

The dynamics of the population-averaged adaptation current reflecting the non-refractory proportion of neurons are well captured by Eq (19) as long as *T*_ref_ is small compared to *τ*_*w*_. In this (physiologically plausible) case 〈*w*〉_*N*_ from Eq (19) can be considered equal to the average adaptation current over the refractory proportion of neurons [16, 48].

#### Mean field limit

For large networks (*N* → ∞) the recurrent input can be approximated by a mean part with additive fluctuations, $I_{\text{rec}}{}_{,\text{i}}/C\approx JKr_{d}\left(t\right)+J\sqrt{Kr_{d}\left(t\right)}\xi_{\text{rec},i}\left(t\right)$ with delayed spike rate

$$r_{d}=r*p_{d},$$ (20)

i.e., the spike rate convolved with the delay distribution, and unit white Gaussian noise process *ξ*_rec,*i*_ that is uncorrelated to that of any other neuron [16, 18, 26, 34].

The step is valid under the assumptions of (i) sufficiently many incoming synaptic connections (*K* ≫ 1) with small enough weights |*J*_*ij*_| in comparison with *V*_T_ − *V*_r_ and sufficient presynaptic activity (diffusion approximation) (ii) that neuronal spike trains can be approximated by independent Poisson processes (Poisson assumption) and (iii) that the correlations between the fluctuations of synaptic inputs for different neurons vanish (mean-field limit). The latter assumption is fulfilled by sparse and uniformly random synaptic connectivity, but also when synaptic strengths *J*_*ij*_ and delays *d*_*ij*_ are independently distributed (in case of less sparse or random connections) [18].

This approximation of the recurrent input allows to replace the overall synaptic current in Eq (14) by *I*_syn,i_ = *C*[*μ*_syn_(*t*, *r*_*d*_) + *σ*_syn_(*t*, *r*_*d*_)*ξ*_*i*_(*t*)] with overall synaptic moments

$$\mu_{\text{syn}}=\mu_{\text{ext}}\left(t\right)+JKr_{d}\left(t\right),\;\sigma_{\text{syn}}^{2}=\sigma_{\text{ext}}^{2}\left(t\right)+J^{2}Kr_{d}\left(t\right),$$ (21)

and (overall) unit Gaussian white noise *ξ*_*i*_ that is uncorrelated to that of any other neuron. Here we have used that external *I*_ext,*i*_ and recurrent synaptic current *I*_rec,*i*_ are independent from each other.

The resulting mean-field dynamics of the membrane voltage is given by

$$\frac{dV_{i}}{dt}=\frac{I_{L}\left(V_{i}\right)+I_{\text{exp}}\left(V_{i}\right)-\left\langle w\right\rangle }{C}+\mu_{\text{syn}}\left(t,r_{d}\right)+\sigma_{\text{syn}}\left(t,r_{d}\right)\xi_{i}\left(t\right),$$ (22)

and corresponds to a McKean-Vlasov type of equation with distributed delays [59] and discontinuity due to the reset mechanism [60] that complements the dynamics of *V*_*i*_ as before. The population-averaged adaptation current 〈*w*〉 = lim_*N* → ∞_〈*w*〉_*N*_ is governed by

d⟨w⟩dt=a(⟨V⟩-Ew2)-⟨w⟩τw+br(t), (23)

with mean membrane voltage (of non-refractory neurons), 〈*V*〉 = lim_*N*→∞_〈*V*〉_*N*_, and spike rate *r* = lim_*N*→∞,Δ*t*→0_ *r*_*N*_(*t*).

Remarks: Instead of exponentially distributed synaptic delays we may also consider other continuous densities *p*_*d*_, identical delays, *p*_*d*_(*τ*) = *δ*(*τ* − *d*) with *d* > 0, or no delays at all, *p*_*d*_(*τ*) = *δ*(*τ*). Instead of identical synaptic strengths one may also consider strengths *J*_*ij*_ that are drawn independently from a normal distribution with mean *J*_*m*_ and variance *J*_*v*_ instead, in which case the overall synaptic moments become *μ*_syn_ = *μ*_ext_(*t*) + *J*_*m*_*Kr*_*d*_(*t*) and $\sigma_{\text{syn}}^{2}=\sigma_{\text{ext}}^{2}\left(t\right)+\left(J_{m}^{2}+J_{v}\right)Kr_{d}\left(t\right)$, cf. [16, 26].

#### Continuity equation

In the membrane voltage evolution, Eq (22), individual neurons are exchangeable as they are described by the same stochastic equations and are coupled to each other exclusively through the (delayed) spike rate via the overall synaptic moments *μ*_syn_ and $\sigma_{\text{syn}}^{2}$. Therefore, the adiabatic and mean-field approximations allow us to represent the collective dynamics of a large network by a (1+1-dimensional) Fokker-Planck equation [16, 18, 26, 34],

$$\frac{\partial }{\partial t}p\left(V,t\right)+\frac{\partial }{\partial V}q_{p}\left(V,t\right)=0\;\text{for}\;V\in \left(-\infty ,V_{s}\right],\;t>0,$$ (24)

which describes the evolution of the probability density *p*(*V*, *t*) to find a neuron in state *V* at time *t* (in continuity form). The probability flux is given by

$$q_{p}\left(V,t\right)=(\frac{I_{L}\left(V\right)+I_{\text{exp}}\left(V\right)}{C}+\mu_{\text{tot}}\left(t\right))\;p\left(V,t\right)-\frac{\sigma_{\text{tot}}^{2}\left(t\right)}{2}\frac{\partial }{\partial V}p\left(V,t\right),$$ (25)

with *total* input mean and standard deviation,

$$\mu_{\text{tot}}\left(t\right)=\mu_{\text{syn}}\left(\mu_{\text{ext}}\left(t\right),r_{d}\left(t\right)\right)-\left\langle w\right\rangle \left(t\right)/C$$ (26)

$$\sigma_{\text{tot}}\left(t\right)=\sigma_{\text{syn}}\left(\sigma_{\text{ext}}\left(t\right),r_{d}\left(t\right)\right).$$ (27)

Note that the mean adaptation current (simply) subtracts from the synaptic mean in the drift term, cf. Eq (22).

The mean adaptation current evolves according to Eq (23) with time-dependent mean membrane voltage (of the non-refractory neurons)

$$\left\langle V\right\rangle =\frac{\int_{-\infty }^{V_{s}}vp\left(v,t\right)dv}{\int_{-\infty }^{V_{s}}p\left(v,t\right)dv}.$$ (28)

The spike rate *r* is obtained by the probability flux through *V*_s_,

$$r\left(t\right)=q_{p}\left(V_{s},t\right).$$ (29)

To account for the reset condition of the aEIF neuron dynamics and ensuring that probability mass is conserved, Eq (24) is complemented by the reinjection condition,

$$q_{p}\left(V_{r}^{+},t\right)-q_{p}\left(V_{r}^{-},t\right)=q_{p}\left(V_{s},t-T_{\text{ref}}\right),$$ (30)

where $q_{p}\left(V_{r}^{+}\right)≔\lim_{V↘V_{r}}q_{p}\left(V\right)$ and $q_{p}\left(V_{r}^{-}\right)≔\lim_{V↗V_{r}}q_{p}\left(V\right)$, an absorbing boundary at *V*_*s*_,

$$p(V_{s},t)=0,$$ (31)

and a natural (reflecting) boundary condition,

$$\lim_{V\to -\infty }q_{p}\left(V,t\right)=0.$$ (32)

Together with the initial membrane voltage distribution *p*(*V*, 0) = *p*_0_(*V*) and mean adaptation current 〈*w*〉(0) = 0 the Fokker-Planck mean-field model is now completely specified.

Note that *p*(*V*, *t*) only reflects the proportion of neurons which are not refractory at time *t*, given by $P\left(t\right)=\int_{-\infty }^{V_{s}}p\left(v,t\right)dv\;=1-\int_{t-T_{\text{ref}}}^{t}r\left(s\right)ds$ (<1 for *T*_ref_ > 0 and *r*(*t*) > 0). The total probability density that the membrane voltage is *V* at time *t* is given by *p*(*V*, *t*) + *p*_ref_(*V*, *t*) with refractory density *p*_ref_(*V*, *t*) = (1 − *P*(*t*)) *δ*(*V* − *V*_r_). At the end of the Methods section we describe how an (optional) spike shape extension for the aEIF model changes the calculation of *p*_ref_ and 〈*V*〉.

In practice we consider a finite reflecting lower barrier *V*_*lb*_ instead of negative infinite for the numerical solution (next section) and for the low-dimensional approximations of the Fokker-Planck PDE (cf. sections below). *V*_lb_ is chosen sufficiently small in order to not distort the free diffusion of the membrane voltage for values below the reset, i.e., *V*_lb_ ≪ *V*_r_. The density *p*(*V*, *t*) is then supported on [*V*_lb_, *V*_*s*_] for each time *t*, and in all expressions above *V* → −∞ is replaced by *V*_lb_.

#### Finite volume discretization

In this work we focus on low-dimensional approximations of the FP model. To obtain a reference for the reduced models it is, however, valuable to solve the (full) FP system, Eqs (24)–(32). Here we outline an accurate and robust method of solution that exploits the linear form of the FP model in contrast to previously described numerical schemes [61, 62] which both require rather small time steps due to the steeply increasing exponential current *I*_exp_ in the flux *q*_*p*_ close to the spike voltage *V*_s_.

We first discretize the (finite) domain [*V*_lb_, *V*_s_] into *N*_*V*_ equidistant grid cells $\left[V_{m-\frac{1}{2}},V_{m+\frac{1}{2}}\right]$ with centers *V*_*m*_ (*m* = 1, …, *N*_*V*_) that satisfy *V*_1_ < *V*_2_ < ⋯ < *V*_*N*_*V*__, where $V_{\frac{1}{2}}=V_{\text{lb}}$ and $V_{N_{V}+\frac{1}{2}}=V_{s}$ are the outmost cell borders. Within each cell the numerical approximation of *p*(*V*, *t*) is assumed to be constant and corresponds to the average value denoted by *p*(*V*_*m*_, *t*). Integrating Eq (24) combined with Eq (30) over the volume of cell *m*, and applying the divergence theorem, yields

$$\frac{\partial }{\partial t}p\left(V_{m},t\right)=\frac{q_{p}\left(V_{m-\frac{1}{2}},t\right)-q_{p}\left(V_{m+\frac{1}{2}},t\right)}{\Delta V}+\delta_{mm_{r}}\frac{1}{\Delta V}q_{p}\left(V_{N_{V}+\frac{1}{2}},t-T_{\text{ref}}\right),$$ (33)

where Δ*V* is the grid spacing and *m*_*r*_ corresponds to the index of the cell that contains the reset voltage *V*_r_. To solve Eq (33) forward in time the fluxes at the borders of each cell need to be approximated. Since the Fokker-Planck PDE belongs to the class of drift-diffusion equations this can be accurately achieved by the first order Scharfetter-Gummel flux [63, 64],

$$q_{p}\left(V_{m+\frac{1}{2}},t\right)=v_{m+\frac{1}{2}}\;\frac{p\left(V_{m},t\right)-p\left(V_{m+1},t\right)\;\exp(-v_{m+\frac{1}{2}}\Delta V/D)}{1-\exp(-v_{m+\frac{1}{2}}\Delta V/D)}\;,$$ (34)

where $v_{m+\frac{1}{2}}\left(t\right)=[I_{L}\left(V_{m+\frac{1}{2}}\right)+I_{\text{exp}}\left(V_{m+\frac{1}{2}}\right)]/C+\mu_{\text{tot}}\left(t,r_{d}\left(t\right),\langle w\rangle \left(t\right)\right)$ and $D\left(t\right)=\frac{1}{2}\sigma_{\text{tot}}^{2}\left(t,r_{d}\left(t\right)\right)$ denote the drift and diffusion coefficients, respectively (cf. Eq (25)). This exponentially fitted scheme [64] is globally first order convergent [65] and yields for large drifts, $|v_{m+\frac{1}{2}}|\Delta V\gg D$, the upwind flux, sharing its stability properties. For vanishing drifts, on the other hand, the centered difference method is recovered [64], leading to more accurate solutions than the upwind scheme in regimes of strong diffusion.

For the time discretization we rewrite Eq (33) (with Eq (34)) in vectorized form and approximate the involved time derivative as first order backward difference to ensure numerical stability. This yields in each time step of length Δ*t* a linear system for the values **p**^*n*+1^ of the (discretized) probability density at *t*_*n*+1_, given the values **p**^*n*^ at the previous time step *t*_*n*_, and the spike rate at the time *t*_*n*+1−*n*_*ref*__ for which the refractory period has just passed,

$$\left(I-\frac{\Delta t}{\Delta V}\;G^{n}\right)p^{n+1}=p^{n}+g^{n+1-n_{\text{ref}}},$$ (35)

with vector elements $p_{m}^{n}=p\left(V_{m},t_{n}\right)$, *m* = 1, …, *N*_*V*_, and $g_{m}^{n+1-n_{\text{ref}}}=\delta_{mm_{r}}\frac{\Delta t}{\Delta V}r\left(t_{n+1-n_{\text{ref}}}\right)$. The refractory period in time steps is given by *n*_*ref*_ = ⌈*T*_ref_/Δ*t*⌉, where the brackets denote the ceiling function, and **I** is the identity matrix. This linear equation can be efficiently solved with runtime complexity $O(N_{V})$ due to the tridiagonal structure of $G^{n}\in R^{N_{V}\times N_{V}}$ which contains the discretization of the membrane voltage (cf. Eqs (33) and (34)), including the absorbing and reflecting boundary conditions (Eqs (31) and (32)). For details we refer to S1 Text.

The spike rate, Eq (29), in this representation is obtained by evaluating the Scharfetter-Gummel flux, Eq (34), at the spike voltage *V*_s_, taking into account the absorbing boundary condition, Eq (31), and introducing an auxiliary ghost cell [66], with center *V*_*N*_*V*_+1_, which yields

$$r\left(t_{n+1}\right)=q_{p}\left(V_{N_{V}+\frac{1}{2}},t_{n+1}\right)=v_{N_{V}+\frac{1}{2}}\;\frac{1+\exp(-v_{N_{V}+\frac{1}{2}}\Delta V/D)}{1-\exp(-v_{N_{V}+\frac{1}{2}}\Delta V/D)}\;p_{N_{V}}^{n+1},$$ (36)

where the drift and diffusion coefficients, $v_{N_{V}+\frac{1}{2}}$ and *D*, are evaluated at *t*_*n*_. The mean membrane voltage (of non-refractory neurons), Eq (28), used for the dynamics of the mean adaptation current, Eq (23), is calculated by $\langle V\rangle \left(t_{n}\right)=\sum_{m=1}^{N_{V}}V_{m}p_{m}^{n}/\sum_{m=1}^{N_{V}}p_{m}^{n}$.

Practically, we use the initialization $p_{m}^{0}=p_{0}\left(V_{m}\right)$ and solve in each time step the linear system, Eq (35), using the function banded_solve from the Python library SciPy [67]. Note that (for a recurrent network or time-varying external input) the tridiagonal matrix **G**^*n*^ has to be constructed in each time step *t*_*n*_, which can be time consuming–especially for small Δ*V* and/or small Δ*t*. Therefore, we employ low-level virtual machine acceleration for this task through the Python package Numba [24] which yields an efficient implementation.

Remark: for a vanishing refractory period *T*_ref_ = 0 the matrix **G**^*n*^ would lose its tridiagonal structure due to the instantaneous reinjection, cf. Eq (36). In this case we enforce a minimal refractory period of one time step, *T*_ref_ = Δ*t*, which is an excellent approximation if the time step is chosen sufficiently small and the spike rate does not exceed biologically plausible values.

### Low-dimensional approximations

In the following sections we present two approaches of how simple spike rate models can be derived from the Fokker-Planck mean-field model described in the previous section, cf. Eqs (20), (21) and (23)–(32).

**Fig 6:**
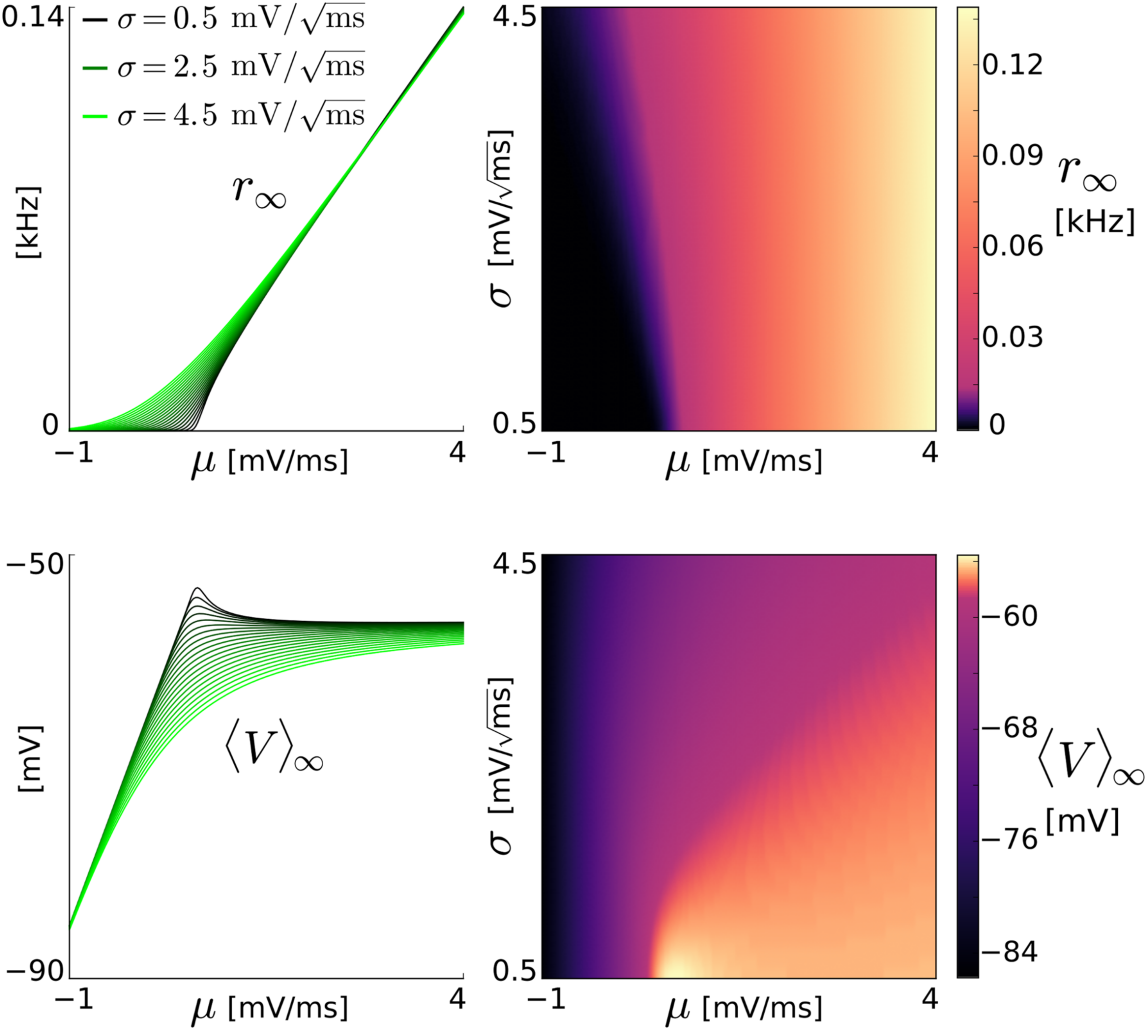
Steady-state spike rate and mean membrane voltage for a population of EIF neurons. *r*_∞_ and 〈*V*〉_∞_ for an uncoupled population of EIF neurons (aEIF with *a* = *b* = 0) as a function of (generic) input mean *μ* and standard deviation *σ*, calculated from the (steady-state) Fokker-Planck equation, shown in two different representations (left and right, each).

The derived models are described by low-dimensional ordinary differential equations (ODEs) which depend on a number of quantities defined in the plane of (generic) input mean and standard deviation (*μ*, *σ*). To explain this concept more clearly we consider, as an example, the steady-state spike rate, which is a quantity required by all reduced models. The steady-state spike rate as a function of *μ* and *σ*,

$$r_{\infty }\left(\mu ,\sigma \right)≔\lim_{t\to \infty }r\left(t;\mu_{\text{tot}}\;=\;\mu ,\sigma_{\text{tot}}\;=\;\sigma \right),$$ (37)

denotes the stationary value of Eq (29) under replacement of the (time-varying) total moments *μ*_tot_ and $\sigma_{\text{tot}}^{2}$ in the probability flux *q*_*p*_, Eq (25), by (constants) *μ* and *σ*^2^, respectively. Thus the steady-state spike rate *r*_∞_ effectively corresponds to that of an uncoupled EIF population whose membrane voltage is governed by *dV*_*i*_/*dt* = [*I*_L_(*V*_*i*_) + *I*_exp_(*V*_*i*_)]/*C* + *μ* + *σξ*_*i*_(*t*) plus reset condition, i.e., adaptation and synaptic current dynamics are detached. For a visualization of *r*_∞_(*μ*, *σ*) see Fig 6.

When simulating the reduced models these quantities need to be evaluated for each discrete time point *t* at a certain value of (*μ*, *σ*) which depends on the overall synaptic moments *μ*_syn_(*t*), $\sigma_{\text{syn}}^{2}\left(t\right)$ and on the mean adaptation current 〈*w*〉(*t*) in a model-specific way (as described in the following Sects.). An example trajectory of *r*_∞_ in the (*μ*, *σ*) space for a network showing stable spike rate oscillations is shown in Fig 5.

Importantly, these quantities depend on the parameters of synaptic input (*J*, *K*, *τ*_*d*_, *μ*_ext_, *σ*_ext_) and adaptation current (*a*, *b*, *τ*_*w*_, *E*_*w*_) only through their arguments (*μ*, *σ*). Therefore, for given parameter values of the EIF model (*C*, *g*_L_, *E*_L_, Δ_T_, *V*_T_, *V*_r_, *T*_ref_) we precalculate those quantities on a (reasonably large and sufficiently dense) grid of *μ* and *σ* values, and access them during time integration by interpolating the quantity values stored in a table. This greatly reduces the computational complexity and enables rapid numerical simulations.

The derived low-dimensional models describe the spike rate dynamics and generally do not express the evolution of the entire membrane voltage distribution. Therefore, the mean adaptation dynamics, which depends on the density *p*(*V*, *t*) (via 〈*V*〉, cf. Eq (23)) is adjusted through approximating the mean membrane voltage 〈*V*〉 by the expectation over the steady-state distribution,

$${\left\langle V\right\rangle }_{\infty }=\frac{\int_{-\infty }^{V_{s}}vp_{\infty }\left(v\right)dv}{\int_{-\infty }^{V_{s}}p_{\infty }\left(v\right)dv},$$ (38)

which is valid for sufficiently slow adaptation current dynamics [48, 58]. The steady-state distribution is defined as *p*_∞_(*V*) = lim_*t* → ∞_ *p*(*V*, *t*; *μ*_tot_ = *μ*, *σ*_tot_ = *σ*), representing the stationary membrane voltages of an uncoupled EIF population for generic input mean *μ* and standard deviation *σ*. The mean adaptation current in all reduced models is thus governed by

$$\frac{d\left\langle w\right\rangle }{dt}=\frac{a({\left\langle V\right\rangle }_{\infty }-E_{w})-\left\langle w\right\rangle }{\tau_{w}}+b\;r\left(t\right),$$ (39)

where the evaluation of quantity 〈*V*〉_∞_ in terms of particular values for *μ* and *σ* at a given time *t* is model-specific (cf. following Sects.). Note again that the calculation of 〈*V*〉_∞_ slightly changes when considering an (optional) spike shape extension for the aEIF model, as described at the end of the Methods section.

The Fokker-Planck model does not restrict the form of the delay distribution *p*_*d*_, except that the convolution with the spike rate *r*, Eq (20), has to be well defined. Here, however, we aim at specifying the complete network dynamics in terms of a low-dimensional ODE system. Exploiting the exponential form of the delay distribution *p*_*d*_ we obtain a simple ordinary differential equation for the delayed spike rate,

$$\frac{dr_{d}}{dt}=\frac{r-r_{d}}{\tau_{d}},$$ (40)

which is equivalent to the convolution *r*_*d*_ = *r* * *p*_*d*_.

Note that more generally any delay distribution from the exponential family allows to represent the delayed spike rate *r*_*d*_ by an equivalent ODE instead of a convolution integral [68]. Identical delays, *r*_*d*_(*t*) = *r*(*t* − *d*), are also possible but lead to delay differential equations. Naturally, in case of no delays, we simply have *r*_*d*_(*t*) = *r*(*t*).

To simulate the reduced models standard explicit time discretization schemes can be applied–directly to the first order equations of the LN_exp_ model, and for the other models (LN_dos_, spec_1_, spec_2_)–to the respective equivalent (real) first order systems. We would like to note that when using the explicit Euler method to integrate any of the latter three low-dimensional models a sufficiently small integration time step Δ*t* is required to prevent oscillatory artifacts. Although the explicit Euler method works well for the parameter values used in this contribution, we have additionally implemented the method of Heun, i.e., the explicit trapezoidal rule, which is second order accurate.

### Spectral models

#### Eigendecomposition of the Fokker-Planck operator

Following and extending [18] we can specify the Fokker-Planck operator L

$$L\left(\mu ,\sigma \right)\left[p\right]=-\frac{\partial }{\partial V}\;[(\frac{I_{L}\left(V\right)+I_{\text{exp}}\left(V\right)}{C}+\mu )\;p]+\frac{\sigma^{2}}{2}\frac{\partial^{2}p}{\partial V^{2}},$$ (41)

for an uncoupled EIF population receiving (constant) input (*μ*, *σ*), cf. Sect. *Low-dimensional approximations*. This operator allows to rearrange the FP dynamics of the recurrent aEIF network, Eq (24), as

$$\frac{\partial p}{\partial t}=L\left(\mu_{\text{tot}}\left(t\right),\sigma_{\text{tot}}\left(t\right)\right)\left[p\right]$$ (42)

which depends on the (time-varying) total input moments *μ*_tot_(*t*, *r*_*d*_, 〈*w*〉) and $\sigma_{\text{tot}}^{2}\left(t,r_{d}\right)$, cf. Eqs (26) and (27), in the drift and diffusion coefficients, respectively.

For each value of (*μ*, *σ*) the operator L possesses an infinite, discrete set of eigenvalues λ_*n*_ in the left complex half-plane including zero [18], i.e., Re{λ_*n*_} ≤ 0, and associated eigenfunctions *ϕ*_*n*_(*V*) (*n* = 0, 1, 2, …) satisfying

$$L\left[\phi_{n}\right]=\lambda_{n}\phi_{n}.$$ (43)

Furthermore, the boundary conditions, Eqs (30)–(32) have to be fulfilled for each eigenfunction *ϕ*_*n*_ separately, i.e., the absorbing boundary at the spike voltage,

$$\phi_{n}\left(V_{s}\right)=0,$$ (44)

and the reflecting barrier at the (finite) lower bound voltage,

$$q_{\phi_{n}}\left(V_{\text{lb}}\right)=0,$$ (45)

must hold. The eigenflux is given by

$$q_{\phi_{n}}\left(V\right)=(\frac{I_{L}\left(V\right)+I_{\text{exp}}\left(V\right)}{C}+\mu )\;\phi_{n}\left(V\right)-\frac{\sigma^{2}}{2}\frac{\partial }{\partial V}\phi_{n}\left(V\right),$$ (46)

i.e., the flux *q*_*p*_ of Eq (25) with eigenfunction *ϕ*_*n*_ and (constant) generic input moments (*μ*, *σ*^2^) instead of density *p* and (time-varying) total input moments, respectively. Moreover, the eigenflux *q*_*ϕ*_*n*__ has to be reinjected into the reset voltage, cf. Eq (30),

$$q_{\phi_{n}}\left(V_{r}^{+}\right)-q_{\phi_{n}}\left(V_{r}^{-}\right)=q_{\phi_{n}}\left(V_{s}\right),$$ (47)

where we have neglected the refractory period, i.e., *T*_ref_ = 0. Note that incorporating a refractory period *T*_ref_ > 0 is straightforward only for the simplified case of vanishing total input moment variations, ${\dot{\mu }}_{\text{tot}}\approx {\dot{\sigma }}_{\text{tot}}^{2}\approx 0$, which is described in the following section and is not captured here in general.

**Fig 7:**
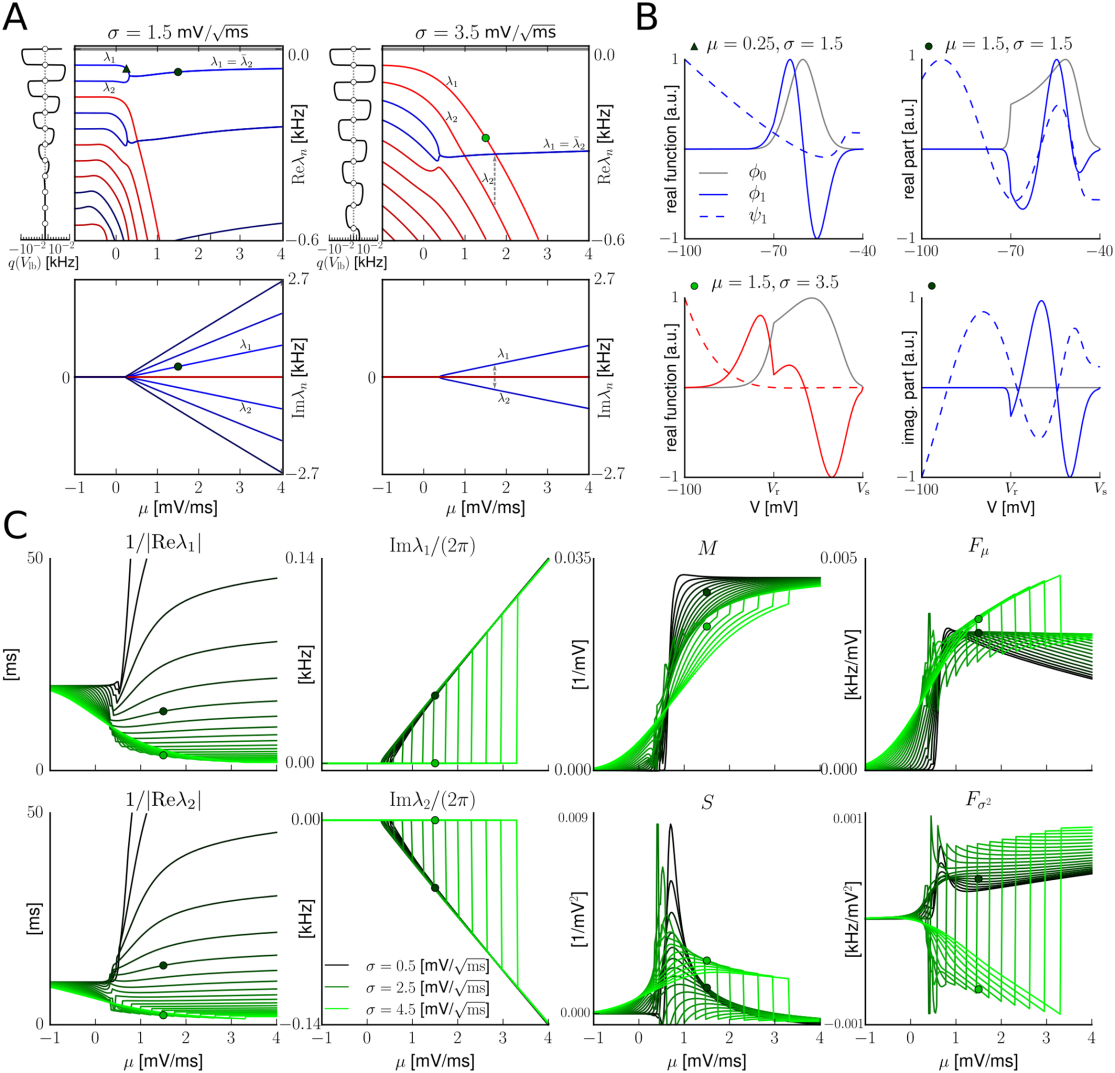
Spectrum of the Fokker-Planck operator L and related quantities. **A**: regular eigenvalues of L (blue) and diffusive ones (red) with real and imaginary part (top and bottom, respectively) as a function of the mean input *μ* for small noise intensity *σ* (left) and larger input fluctuations (right). The first two dominant eigenvalues λ_1_, λ_2_ are indicated together with discontinuities in the real (λ_2_) and imaginary part (λ_1_ and λ_2_), respectively. The stationary eigenvalue λ_0_ = 0 is shown in gray. Note that the value of the mean input *μ* at which the eigenvalue λ_*n*_ changes from real to complex values depends on the noise amplitude *σ* and the eigenvalue index *n* which is difficult to see in the figure. The narrow winding curves attached to the left side of the respective spectra represent the lower bound flux *q*(*V*_lb_) for *μ*_min_ = −1.5 mV/ms as a function of (real) eigenvalue candidate λ. The flux axis has a logarithmic scale between the large ticks (absolute values between 10^−10^ and 10^−2^ kHz) and is linear around the dashed zero value. The open circles denote the eigenvalues, i.e., those λ that satisfy *q*(*V*_lb_) = 0. Note that *q*(*V*_lb_) ranges over several orders of magnitude. **B**: stationary eigenfunction *ϕ*_0_ = *p*_∞_ (gray) and nonstationary eigenfunctions *ϕ*_1_ of L and *ψ*_1_ of $L^{*}$ corresponding to the first dominant eigenvalue λ_1_ for three different input parameter values indicated by the triangle and circles in **A** (same units of *μ* and *σ* as therein). The eigenfunctions are biorthonormalized, but (only) for visualization truncated at *V* = −100 mV and furthermore individually scaled to absolutely range within the unit interval of arbitrary units [a.u.]. **C**: first and second dominant eigenvalues λ_1_, λ_2_ with real and imaginary part (that are also indicated in **A**), as well as additional (real-valued) quantities of the model spec_2_ (*M*, *S*, *F*_*μ*_, *F*_*σ*^2^_) as a function of input mean *μ* and noise strength *σ* in steps of $0.2\;\text{mV}/\sqrt{\text{ms}}$ from small values (black) to larger ones (green). The dots indicate identical parameter values to the spectra of **A** and the eigenfunctions of **B** (darker: $\sigma =1.5\;\text{mV}/\sqrt{\text{ms}}$, brighter green: $\sigma =3.5\;\text{mV}/\sqrt{\text{ms}}$).

The spectrum of L is shown in Fig 7A and further discussed in Sect. *Remarks on the spectrum*.

Defining the non-conjugated [69] inner product $\langle \psi ,\phi \rangle =\int_{V_{\text{lb}}}^{V_{s}}\psi \left(v\right)\phi \left(v\right)dv$ yields the corresponding adjoint operator $L^{*}\left(\mu ,\sigma \right)$ given by [18]

$$L^{*}=(\frac{I_{L}\left(V\right)+I_{\text{exp}}\left(V\right)}{C}+\mu )\;\frac{\partial }{\partial V}+\frac{\sigma^{2}}{2}\frac{\partial^{2}}{\partial V^{2}},$$ (48)

which satisfies $\langle \psi ,L\phi \rangle =\langle L^{*}\psi ,\phi \rangle$ for any complex-valued functions *ψ* and *ϕ* that are sufficiently smooth on [*V*_lb_, *V*_s_]. $L^{*}$ has the same set of eigenvalues λ_*n*_ as L but distinct associated eigenfunctions *ψ*_*n*_(*V*), i.e.,

$$L^{*}\left[\psi_{n}\right]=\lambda_{n}\psi_{n},$$ (49)

which have to satisfy three boundary conditions,

$$\psi_{n}\left(V_{s}\right)=\psi_{n}\left(V_{r}\right),$$ (50)

$$\frac{\partial \psi_{n}}{\partial V}\left(V_{\text{lb}}\right)=0,$$ (51)

$$\frac{\partial \psi_{n}}{\partial V}\left(V_{r}^{+}\right)=\frac{\partial \psi_{n}}{\partial V}\left(V_{r}^{-}\right)$$ (52)

that are determined by integrating $\langle \psi_{n},L\left[\phi_{n}\right]\rangle$ by parts and equalling with $\langle L^{*}\left[\psi_{n}\right],\phi_{n}\rangle$ using the conditions of L, Eqs (44)–(47). Note that the last condition, Eq (52), ensures a continuous derivative of *ψ*_*n*_ at *V*_r_ in contrast to the eigenfunctions *ϕ*_*n*_ of L, that have a kink at the reset due to reinjection condition, Eq (47), as shown in Fig 7B.

The eigenfunctions of L and $L^{*}$ are pairwise orthogonal and in the following (without loss of generality) assumed to be scaled according to the biorthonormality condition,

$$\left\langle \psi_{n},\phi_{m}\right\rangle =\delta_{nm}.$$ (53)

The membrane voltage probability density can now be expanded onto the (moving) eigenbasis of L [18, 70],

$$p\left(V,t\right)=\sum_{n=0}^{\infty }\alpha_{n}\left(t\right)\phi_{n}\left(V\right),$$ (54)

where each eigenfunction *ϕ*_*n*_ depends on time via the total input moments *μ* = *μ*_tot_(*t*, *r*_*d*_, 〈*w*〉), $\sigma^{2}=\sigma_{\text{tot}}^{2}\left(t,r_{d}\right)$, and the projection coefficients are given by *α*_*n*_ = 〈*ψ*_*n*_, *p*〉 and particularly *α*_0_ = 1 [18]. Deriving *α*_*n*_ with respect to time (for *n* = 1, 2, …) and using the expansion, Eq (54), the Fokker-Planck Eq (42) as well as the definition of the adjoint operator yields an infinite-dimensional equation for the complex-valued projection coefficients ***α***(*t*) = (*α*_1_(*t*), *α*_2_(*t*), …)^*T*^,

$$\dot{\alpha }=(\Lambda +C_{\mu }\;{\dot{\mu }}_{\text{tot}}+C_{\sigma^{2}}\;{\dot{\sigma }}_{\text{tot}}^{2})\;\alpha +c_{\mu }\;{\dot{\mu }}_{\text{tot}}+c_{\sigma^{2}}\;{\dot{\sigma }}_{\text{tot}}^{2}.$$ (55)

This dynamics is initialized by *α*_*n*_(0) = 〈*ψ*_*n*_, *p*_0_〉, and is complemented by (i) an expression for the spike rate,

$$r\left(t\right)=r_{\infty }+f\cdot \alpha ,$$ (56)

that is obtained from Eq (29) (using Eq (54)), and (ii) by the mean adaptation and delayed spike rate dynamics, Eqs (39) and (40). The dots in Eqs (55) and (56) denote time derivatives (e.g., ${\dot{\sigma }}_{\text{tot}}^{2}\;=d\sigma_{\text{tot}}^{2}/dt$) and a non-conjugated scalar product of complex vectors (i.e., $f\cdot \alpha =\sum_{n=1}^{\infty }f_{n}\alpha_{n}$), respectively. The matrix **Λ** = diag(λ_1_, λ_2_, …) contains the eigenvalues of L, the matrices **C**_*μ*_ and **C**_*σ*^2^_ have elements (**C**_*x*_)_*n*,*m*_ = 〈∂_*x*_*ψ*_*n*_, *ϕ*_*m*_〉 for *x* ∈ {*μ*, *σ*^2^} and *n*, m∈N with partial derivative ∂_*x*_ = ∂/∂*x*, the vectors **c**_*μ*_ and **c**_*σ*^2^_ consist of components $c_{n}^{x}=\langle \partial_{x}\psi_{n},\;\phi_{0}\rangle$. The steady-state spike rate is given by *r*_∞_ = *q*_*ϕ*_0__(*V*_s_), i.e., the flux of the eigenfunction *ϕ*_0_ = *p*_∞_ that represents the stationary membrane voltage distribution *p*_∞_ and corresponds to the (stationary) eigenvalue λ_0_ = 0 [18]. The vector **f** contains the (nonstationary) eigenfluxes evaluated at the spike voltage, *f*_*n*_ = *q*_*ϕ*_*n*__(*V*_s_).

Note that the quantities (**Λ**, **C**_*μ*_, **C**_*σ*^2^_, **c**_*μ*_, **c**_*σ*^2^_, *r*_∞_, **f**, 〈*V*〉_∞_) all depend on time in Eqs (55), (56) and (39) via the total input moments (*μ*_tot_, $\sigma_{\text{tot}}^{2}$). Particularly, at time *t* the (biorthonormal) solution of the eigenvalue problems for L and its adjoint $L^{*}$, Eqs (43)–(47) and (49)–(52) with $n\in N_{0}$, is required for *μ* = *μ*_tot_(*t*, *r*_*d*_, 〈*w*〉), *σ* = *σ*_tot_(*t*, *r*_*d*_). Because L is a real operator its spectrum contains only real eigenvalues and/or complex conjugated pairs depending on (*μ*, *σ*). This property carries over to the eigenfunctions and therefore also to the components of all quantities above implying for example that the scalar product **f** · ***α*** is always real-valued.

Although the spectral representation, Eqs (55) and (56), is fully equivalent to the original (partial differential) Fokker-Planck equation without refractory period and while it contains only time derivatives it is still an infinite-dimensional and furthermore generally an implicit model. Therefore, to derive an explicit low-dimensional ordinary differential model for the spike rate *r*(*t*), it is not sufficient to truncate the expansion in Eq (54) after, e.g., two terms but additional assumptions have to be considered.

#### Basic model: One eigenvalue, negligible input variations

The first and simpler, derived spectral model is based on [19] and requires the strong assumption of vanishing changes of the total input moments, ${\dot{\mu }}_{\text{tot}}\approx 0$, ${\dot{\sigma }}_{\text{tot}}^{2}\;\approx 0$. Under this approximation the projection coefficient dynamics, Eq (55), simplifies to

$${\dot{\alpha }}_{n}=\lambda_{n}\alpha_{n}$$ (57)

for n∈N. Considering only the dominant (nonzero) eigenvalue

$$\lambda_{1}=\text{argmin}\{|\text{Re}\left\{\lambda_{n}\right\}\left|:\lambda_{n}\neq 0\right\},$$ (58)

i.e., the “slowest mode”, we obtain from Eqs (56) and (57) that *r*(*t*) = *r*_∞_ + *m*_1_ Re{*f*_1_*α*_1_} with *m*_1_ = 1 if $\lambda_{1}\in R$ and *m*_1_ = 2 if $\lambda_{1}\in C\setminus R$. Here we have included for a complex eigenvalue λ_1_ also its complex conjugate ${\bar{\lambda }}_{1}$ that has the projection coefficient $\bar{\alpha_{1}}\left(t\right)$. They jointly yield a zero imaginary part in the scalar product of Eq (56). Defining $\tilde{r}\left(t\right)=r_{\infty }+m_{1}f_{1}\alpha_{1}$ yields the complex first order equation for the spike rate [19],

$$\dot{\tilde{r}}=\lambda_{1}\left(\tilde{r}-r_{\infty }\right),\;r\left(t\right)=\text{Re}\left\{\tilde{r}\right\}.$$ (59)

While this derivation is based on neglecting changes of the total input moments, their time-variation is effectively reintroduced in the two quantities (dominant eigenvalue and steady-state rate), i.e., λ_1_(*μ*_tot_, *σ*_tot_) and *r*_∞_(*μ*_tot_, *σ*_tot_) with *μ*_tot_(*t*) and *σ*_tot_(*t*) according to Eqs (26) and (27). Therefore, the spike rate evolution, Eq (59), is complemented with the dynamics of mean adaptation current 〈*w*〉 and delayed spike rate *r*_*d*_, Eqs (39) and (40), where the former involves the third (*μ*_tot_, *σ*_tot_)-dependent quantity 〈*V*〉_∞_. See Figs 6 and 7C for the involved quantities depending on (generic) input *μ*, *σ*.

We call this first derived low-dimensional spike rate model, i.e., Eqs (59), (39) and (40), spec_1_. It is very simple in comparison with the full Fokker-Planck system in the spectral representation, Eqs (55)–(56), (39) and (40), in the sense that it does not depend on “nonstationary” quantities of L or its adjoint $L^{*}$ except for the dominant eigenvalue λ_1_.

Note that under the assumption of vanishing input moment variations the dynamics of the expansion coefficients *α*_*n*_, Eq (55), is simply exponentially decaying in time, cf. Eq (57). This allows to incorporate a refractory period *T*_ref_ > 0 into the spectral decomposition framework by inserting the eigenbasis expansion of the membrane voltage distribution, Eq (54), into the reinjection condition of the Fokker-Planck model, Eq (30), using *α*_*n*_(*t*) = *α*_*n*_(0) exp(λ_*n*_*t*) and the absorbing boundary, Eq (44). This generalizes the reinjection condition for the eigenfunctions *ϕ*_*n*_ of L from Eq (47) to

$$q_{\phi_{n}}\left(V_{r}^{+}\right)-q_{\phi_{n}}\left(V_{r}^{-}\right)=q_{\phi_{n}}\left(V_{s}\right)\exp\left(-\lambda_{n}T_{\text{ref}}\right),$$ (60)

which was applied in [19], and the corresponding boundary condition for the adjoint operator’s eigenfunctions *ψ*_*n*_ from Eq (50) to

$$\psi_{n}\left(V_{s}\right)=\psi_{n}\left(V_{r}\right)\exp\left(-\lambda_{n}T_{\text{ref}}\right).$$ (61)

#### Advanced model: Two eigenvalues, slow input moment variations

Another possibility to derive a low-dimensional spike rate model is based on ongoing work of Maurizio Mattia. He has recognized that under the weaker (compared to the basic spec_1_ model above) assumption of small but not vanishing input moment changes a real-valued second order ordinary differential equation for the spike rate *r*(*t*) can be consistently derived [20]. Here we extend this approach to account for neuronal adaptation, time-varying external input moments and delay distributions.

The steps in a nutshell (for the detailed derivation see S1 Text) are (i) taking the derivative of Eq (55) (once) and of (56) (twice) w.r.t. time, (ii) considering only the first two dominant eigenvalues λ_1_ and λ_2_, i.e., neglecting all (faster) eigenmodes that correspond to eigenvalues with larger absolute real part (“modal approximation”), (iii) assuming slowly changing input moments, i.e., small ${\dot{\mu }}_{\text{tot}}$ and ${\dot{\sigma }}_{\text{tot}}^{2}$ that allow to consider projections coefficients of that order, i.e., $\alpha_{n}=O\left({\dot{\mu }}_{\text{tot}}\right)$ and $\alpha_{n}=O\left({\dot{\sigma }}_{\text{tot}}^{2}\right)$ for *n* = 1, 2, and therefore to consider only linear occurences of ${\dot{\mu }}_{\text{tot}}$, ${\dot{\sigma }}_{\text{tot}}^{2}$, *α*_1_, *α*_2_, and neglect terms of second and higher order. The slowness approximation implies that neither the external moments *μ*_ext_(*t*), $\sigma_{\text{ext}}^{2}\left(t\right)$ nor the (delayed) spike rate *r*_*d*_(*t*) nor the population-averaged adaptation current 〈*w*〉(*t*) should change very fast (cf. Eqs (26), (27) and (21). Note that the dynamics of 〈*w*〉 is assumed to be slow already, cf. Sect. *Fokker-Planck system*.

Under these approximations we obtain for the spike rate dynamics the following real second order ODE,

$$\beta_{2}\;\ddot{r}+\beta_{1}\;\dot{r}+\beta_{0}\;r=r_{\infty }-r-\beta_{c},$$ (62)

that is complemented with the mean adaptation current and delayed spike rate dynamics, Eqs (39) and (40), and we call the model spec_2_. The coefficients

$$\beta_{2}=\;\;D,$$ (63)

$$\beta_{1}=\;-T+DM\frac{b}{C}-\frac{R}{\tau_{d}},$$ (64)

$$\beta_{0}=-DM\frac{b}{\tau_{w}C}-\frac{b}{C}H_{\mu }+\frac{1}{\tau_{d}}\;(KJH_{\mu }+KJ^{2}H_{\sigma^{2}})+\frac{R}{\tau_{d}^{2}},$$ (65)

$$\begin{array}{ccc} \beta_{\text{c}} & = & \;\;r_{d}\;(-\frac{1}{\tau_{d}}(KJH_{\mu }+\;KJ^{2}H_{\sigma^{2}})-\frac{R}{\tau_{d}^{2}})\\ & - & ({\ddot{\mu }}_{\text{ext}}+\frac{a({\left\langle V\right\rangle }_{\infty }-E_{w})-\left\langle w\right\rangle }{\tau_{w}^{2}C})\;DM-\ddot{\sigma_{\text{ext}}^{2}\;}\;DS\\ & + & ({\dot{\mu }}_{\text{ext}}-\frac{a({\left\langle V\right\rangle }_{\infty }-E_{w})-\left\langle w\right\rangle }{\tau_{w}C})\;H_{\mu }+\dot{\sigma_{\text{ext}}^{2}\;}\;H_{\sigma^{2}}, \end{array}$$ (66)

depend on the (lumped) quantities *D* = 1/λ_1_ ⋅ 1/λ_2_, *T* = 1/λ_1_ + 1/λ_2_, *M* = ∂_*μ*_*r*_∞_ + **f** ⋅ **c**_*μ*_, *S* = ∂_*σ*^2^_*r*_∞_ + **f** ⋅ **c**_*σ*^2^_, *R* = *DMKJ* + *DSKJ*^2^, *H*_*μ*_ = *TM* + *DMa*∂_*μ*_〈*V*〉_∞_/(*τ*_*w*_*C*) − *DF*_*μ*_, *H*_*σ*^2^_ = *TS* + *DMa*∂_*σ*^2^_〈*V*〉_∞_/(*τ*_*w*_*C*) − *DF*_*σ*^2^_, *F*_*μ*_ = **f** ⋅ **Λ** **c**_*μ*_ and *F*_*σ*^2^_ = **f** ⋅ **Λ** **c**_*σ*^2^_. Here the diagonal eigenvalue matrix **Λ** = diag(λ_1_, λ_2_) and the vectors **f** = (*f*_1_, *f*_2_)^T^, $c_{\mu }={\left(c_{1}^{\mu },c_{2}^{\mu }\right)}^{T}$, $c_{\sigma^{2}}={\left(c_{1}^{\sigma^{2}},c_{2}^{\sigma^{2}}\right)}^{T}$ are two-dimensional in contrast to infinite as in the original dynamics, Eqs (55) and (56). These individual quantities, that also include 〈*V*〉_∞_ (cf. Eq (39)), *r*_∞_ and derivatives of both w.r.t. generic mean *μ* and variance *σ*^2^, are evaluated at the total input moments *μ* = *μ*_tot_(*t*, *r*_*d*_, 〈*w*〉), $\sigma^{2}=\sigma_{\text{tot}}^{2}\left(t,r_{d}\right)$. A relevant subset of individual and lumped quantities is shown in Figs 6 and 7C.

The four coefficients, Eqs (63)–(66), are real-valued because we define λ_2_ as the second dominant eigenvalue conditioned that (λ_1_, λ_2_) compose either a real or a complex conjugated eigenvalue pair, i.e.,

$$\lambda_{2}=\text{argmin}\{|\text{Re}\left\{\lambda_{n}\right\}\left|\;:\;\lambda_{n}\neq \lambda_{1}\;\;\text{s.t.}\;\;\lambda_{n}\neq 0,\;\lambda_{1}+\lambda_{n}\in R\right\},$$ (67)

where λ_1_ is obtained as for the (basic) spec_1_ model, cf. Eq (58). This condition ensures that all related complex quantities occur in vectors of two complex conjugate components (for example, $f_{1}=\bar{f_{2}}$) implying that the scalar products above are real-valued (e.g., $f\cdot c_{\mu }\in R$) and therefore all (nine) lumped quantities, too. Note that this specific definition of λ_2_ is required only for integrate-and-fire neuron models that have a lower bound different from the reset, i.e., *V*_lb_ < *V*_r_, as discussed in the following section.

The coefficients of the spec_2_ model require–in addition to eigenvalues λ_*n*_ and steady-state rate *r*_∞_ (cf. basic spec_1_ model, Eq (59))–quantities that involve the first and second eigenfunctions of the Fokker-Planck operator L and its adjoint $L^{*}$. Additionally, *β*_1_, *β*_0_ and *β*_*c*_ contain the parameters of membrane voltage (*C*) and mean adaptation current dynamics (*a*, *b*, *τ*_*w*_, *E*_*w*_) as well as of the recurrent coupling (*K*, *J*, *τ*_*d*_) and, importantly, explicit dependencies on the population-averaged adaptation current 〈*w*〉 and the delayed spike rate *r*_*d*_ (that is in addition to implicitly via *μ*_tot_ and *σ*_tot_). Furthermore *β*_*c*_ depends on the first two time derivatives of the external input moments *μ*_ext_(*t*) and $\sigma_{\text{ext}}^{2}\left(t\right)$. This explicit occurence of neuronal and coupling parameters, state variables and input moment derivatives in the coefficients is not expressed in the basic spectral model (spec_1_), Eq (59). A consequence is that for the (advanced) model spec_2_ the external moments have to be provided twice differentiable or in case of non-smooth time series to be filtered (e.g., see Sect. *Performance for variations of the mean input*).

The particular coefficients, Eqs (63)–(66), are specific for the choice of an exponential delay distribution, as indicated by the occurrence of the mean delay *τ*_*d*_ in *β*_1_, *β*_0_ and *β*_*c*_. Other choices such as identical delays or no delays are described in the supplementary material S1 Text.

Note that the original (infinite-dimensional) spectral dynamics, Eqs (55) and (56), assumes for the refractory period a value of *T*_ref_ = 0 which carries over to the same choice for the spec_2_ model, whereas *T*_ref_ > 0 is not supported (yet).

The spike rate is by definition nonnegative, however, the model spec_2_, Eq (62), can yield negative rates *r*(*t*), especially for small total mean input *μ*_tot_ and fast (external) input changes, e.g., when ${\dot{\mu }}_{\text{ext}}$ is large. We explicitly avoid that behaviour by setting both the spike rate of this model, *r*, and its derivative, $\dot{r}$, to zero whenever *r*(*t*)<0 and continue the integration of the differential equation afterwards.

#### Remarks on the spectrum

In the previous two sections we have developed two spike rate models based on approximations of the Fokker-Planck system’s spectral representation, Eqs (55) and (56) under different slowness assumptions. In the derivation of the (simple) model spec_1_, cf. Eq (59), temporal variations of the total input moments are completely neglected while the (advanced) model spec_2_, cf. Eq (62), incorporates (slow) changes of the total input moments through linear terms (proportional to ${\dot{\mu }}_{\text{tot}}$ or ${\dot{\sigma }}_{\text{tot}}^{2}$) and neglects (faster) quadratic and higher order ones.

Both slowness approximations imply that the eigenvalue matrix Λ is the dominant term in the homogeneous part of Eq (55). Therefore the eigenvalues approximately correspond to decay time constants 1/|Re{λ_*n*_}| in their real part and in case of complex eigenvalues they contribute damped oscillatory components with frequency |Im{λ_*n*_}|/(2*π*) through their imaginary part to the dynamics. How the spectrum of the Fokker-Planck operator L depends on the input moments therefore gives insights into the behavior of the derived spike rate models spec_1_ and spec_2_, and of the FP model with slow adaptation in general.

Here we summarize the main properties of the eigenvalues λ_*n*_(*μ*, *σ*) for the (uncoupled, nonadaptive) EIF neuron model as a function of generic input mean *μ* and standard deviation *σ* (cf. Sect. *Low-dimensional approximations*) that are shown in Fig 7A. Note that at time *t* the total input determines the particularly effective eigenvalue, λ_*n*_(*μ*_tot_(*t*), *σ*_tot_(*t*)), in dependence of external input moments, (delayed) spike rate and adaptation current, cf. Eqs (26), (27) and (21. We thereby extend the findings of [18] concerning the perfect integrate-and-fire neuron with reflecting lower barrier at the reset voltage (PIFb), i.e., *dV*_*i*_/*dt* = *μ* + *σξ*_*i*_(*t*) with *V*_lb_ = *V*_r_.

1. The eigenvalue λ_0_ = 0 exists for all (generic) input moments *μ*, *σ*^2^ with stationary membrane voltage distribution as corresponding eigenfunction, *ϕ*_0_ = *p*_∞_ (see Fig 7A and 7B), the respective adjoint eigenfunction is constant, *ψ*_0_ = 1, cf. [18]. The other, nonstationary eigenvalues λ_*n*_ with *n* ≥ 1 have negative real parts for all (*μ*, *σ*) which yields stability of the stationary distribution for constant total input moments. For sufficiently small mean input *μ*_min_ the eigenvalues λ_*n*_(*μ*_min_, *σ*) are real-valued for all *n* and *σ*, i.e., no (damped) oscillatory spike rate transient in that regime are present (consistent with [18]), cf. Fig 7A.
2. Two classes of modes can be distinguished: eigenvalues of the first kind occur in couples which are real-valued at *μ*_min_ but merge for increasing mean input *μ* to become a complex conjugated pair of decreasing absolute real part and almost linearly increasing imaginary part (see Fig 7A). Thus they correspond in this situation to damped oscillatory dynamics with increased frequency |Im{λ_*n*_}|/(2*π*) and decay time constant 1/|Re{λ_*n*_}| for stronger mean input. Note that there is no single critical parameter for the real-to-complex transition, instead that depends on the input mean *μ* and standard deviation *σ* as well as on the eigenvalue index *n* in contrast to the PIFb neuron where *μ* = 0 (alone) induces the transition [18]. We call this type of eigenvalue *regular* because it is observed also for very simple integrate-and-fire models (such as PIFb).
  The second type of eigenvalue is real for the whole input parameter space of (*μ*, *σ*). The corresponding decay time constant is large if noise dominates while for stronger mean input the respective dynamics is negligibly fast. Note that by setting the lower bound *V*_lb_ equal to the reset voltage, this eigenvalue class is completely removed for the EIFb model, which is the EIF membrane voltage description with *V*_lb_ = *V*_r_ (not used here). This explains why this new type of eigenvalue has not been described in [18], and suggests a relationship to the diffusion of the membrane voltage for hyperpolarized neuronal states. The latter correspondence is further supported from the significant values of the respective eigenfunction *ϕ*_*n*_ below the reset voltage *V*_r_ in contrast to the eigenfunctions of regular eigenvalues (cf. Fig 7B). Therefore we label this second type of eigenvalue *diffusive*.
3. The input noise intensity *σ* controls the spectrum’s mixture of the two eigenvalue classes as follows: weak noise favors regular modes, i.e., the dominant two eigenvalues are pairs of real (for smaller mean input *μ*) or complex conjugated eigenvalues (for larger *μ*) while the diffusive modes are irrelevantly fast in this regime (cf. Fig 7A, left). Increased input fluctuations, i.e., a larger *σ*, on the other hand leads to a spectrum with dominant (“slowest” eigenvalue λ_1_) of the diffusive kind for small mean input *μ* while for larger *μ* the dominant mode is from the regular class (being real or complex depending on *μ*, *σ*, *n*), see Fig 7A, right. Furthermore an increased noise strength *σ* leads to a smaller decay time constant 1/|Re{λ_*n*_}| for regular modes, i.e., their respective contribution to the spike rate dynamics is faster in the fluctuation-dominated regime than in the drift-dominated one, whereas for diffusive modes the opposite holds true.

The specific definition of the second dominant eigenvalue λ_2_, Eq (67), is necessary to ensure real coefficients in the spec_2_ model, Eqs (63)–(66), for regions in (*μ*, *σ*)-space where the first dominant eigenvalue λ_1_ is of the (real) diffusive type and the second is part of a complex conjugate couple (for an example see Fig 7A, right).

Extracting the required dominant eigenvalues λ_1_ (for both spectral models: spec_1_, spec_2_) and λ_2_ (for spec_2_ model only) according to Eqs (58) and (67) in the (*μ*, *σ*)-plane leads to points of instantaneous changes in the imaginary part (λ_1_ and λ_2_) and the real part (only λ_2_) due to transitions from a dominant diffusive mode for small mean input *μ* to a regular eigenvalue (or pair) becoming dominant for larger *μ* (see Fig 7A and 7C and the last property above). These discontinuities (which lie on a one-dimensional curve *μ*(*σ*)) could be avoided by either restricting to *V*_lb_ = *V*_r_ (no diffusive modes) or by deriving a third spectral model based on three eigenvalues: the dominant regular pair together with the dominant diffusive eigenvalue. Making the latter extension is straightforward by using the same steps and slowness approximation as for the model spec_2_ and would yield a 3rd order ODE for the spike rate *r*(*t*) with smooth coefficients and is expected to show increased reproduction accuracy (especially for small mean input) compared to the model spec_2_.

The properties above imply for the low-dimensional models spec_1_ and spec_2_, that both enable damped oscillatory spike rates for mean-dominated input (for large *μ*) since then the first two dominant eigenvalues (λ_1_ and λ_2_) have nonzero imaginary parts and are complex conjugates of each other (see Fig 7C). Furthermore in this case the effective time constant, 1/|Re{λ_1_}| = 1/|Re{λ_2_}|, is large (especially for small input standard deviation *σ*). For noise-dominated input, i.e., when *σ* (and not *μ*) is large, on the other hand, the corresponding spike rate dynamics is fast and does not contain an oscillatory component.

#### Numerical solver

Here we present a numerical solution method of the Fokker-Planck boundary eigenvalue problem (BEVP) for the operator L(μ,σ), Eqs (43)–(47), and its adjoint $L^{*}\left(\mu ,\sigma \right)$, Eqs (49)–(52). The solution of the two BEVPs in terms of eigenvalues λ_*n*_ and associated (biorthonormal) eigenfunctions (*ϕ*_*n*_(*V*), *ψ*_*n*_(*V*)) as well as quantities that are derived from those and are required by the models spec_1_ and spec_2_, is obtained for a rectangle of the input mean *μ* and standard deviation *σ* (see Sect. *Low-dimensional Approximations*). Note that the numerical method is not restricted to the EIF neuron model and supports other integrate-and-fire models as well (e.g., perfect, leaky or quadratic).

The eigenequation L(μ,σ)[ϕ]=λϕ, i.e., Eq (43) (omitting the eigenvalue index *n*), represents a second order ODE (cf. Eq (41)), that is equivalent to the following first order system for the eigenflux *q*_*ϕ*_(*V*) and the eigenfunction *ϕ*(*V*),

$$-\frac{d}{dV}\left(\begin{array}{c} q_{\phi }\\ \phi \end{array}\right)=\underset{=\text{A}}{\underset{︸}{\left(\begin{array}{cc} 0 & \lambda \\ \frac{2}{\sigma^{2}} & -2\frac{g\left(V\right)+\mu }{\sigma^{2}} \end{array}\right)}}\;\left(\begin{array}{c} q_{\phi }\\ \phi \end{array}\right)$$ (68)

with coefficient matrix **A**(*V*) that has a nonlinear component through *g*(*V*) = [*I*_L_(*V*) + *I*_exp_(*V*)]/*C* that contains leak and exponential (membrane) currents. Here it was used the form of the eigenflux *q*_*ϕ*_ (cf. Eq (46)), and that $L\left(\mu ,\sigma \right)\left[\phi \right]=-\partial_{V}q_{\phi }$ for generic input moments *μ* and *σ*^2^ (cf. Eq (41)).

Basically a direct discretization, e.g., by a finite difference approximation, of the membrane voltage derivatives in the System (68) can be applied. In combination with the boundary conds., Eqs (44)–(47), this leads to a (sparse) matrix eigenvalue problem that allows for application of standard (Arnoldi iteration based) numerical solvers. However, the convergence properties of this approach are very poor in the sense that extremely small voltage steps Δ*V* have to be chosen for the finite differences. Thus, the technique is inefficient as huge systems appear or even inaccurate due to amplified round-off errors by ill-conditioned matrices.

Here we propose an alternative solution procedure which is based on a reformulation of the System (68) with corresponding boundary conditions, Eqs (44)–(47), as a complex-valued algebraic root finding problem

$$\lambda \;\mapsto \;q_{\phi }\left(V_{\text{lb}};\lambda \right)\overset{!}{=}0$$ (69)

whose solutions are the eigenvalues λ_*n*_. To evaluate the nonlinear function (the left hand side of this equation) for an arbitrary λ∈C (not necessary an eigenvalue) Eq (68) is integrated backward starting from the spike voltage *V*_s_ (initializing one component to satisfy the absorbing boundary cond., Eq (44), i.e., *ϕ*(*V*_s_) = 0, and another component that can be chosen arbitrarily, $q_{\phi }\left(V_{s}\right)\in C\setminus \left\{0\right\}$, due to the linearity of the problem) via the reset *V*_r_ (where the reinjection cond., Eq (47), is enforced, i.e., $q_{\phi }\left(V_{r}^{-}\right)=q_{\phi }\left(V_{r}^{+}\right)-q_{\phi }\left(V_{s}\right)$, which induces a discontinuity in *q*_*ϕ*_ at the reset voltage) finally to the lower bound voltage *V*_lb_. There, a nonzero value of $q_{\phi }\left(V_{\text{lb}}\right)\in C$ indicates that λ is not an eigenvalue since in this case *ϕ*(*V*) violates the reflecting boundary condition, Eq (45). *q*_*ϕ*_(*V*_lb_) = 0 on the other hand shows that λ is an eigenvalue with corresponding eigenfunction *ϕ*(*V*) and eigenflux *q*_*ϕ*_(*V*). Note that when considering a nonzero refractory period the generalized version of the reinjection cond., Eq (60), i.e., $q_{\phi }\left(V_{r}^{-}\right)=q_{\phi }\left(V_{r}^{+}\right)-q_{\phi }\left(V_{s}\right)e^{-\lambda T_{\text{ref}}}$, is enforced instead of the expression above. The latter is only valid for the spec_1_ model, Eq (59) (see Sect. *Basic model: one eigenvalue, negligible input variations*) and makes the Fokker-Planck eigenvalue problem nonlinear due to the exponentiation of λ in Eq (60).

The (complex-valued) root finding problem, Eq (69) can be solved numerically to yield a target eigenvalue λ_*n*_ using an iterative procedure (for example a variant of Newton’s method) given that a sufficiently close initial approximation ${\tilde{\lambda }}_{n}\in C$ is available. In our Python implementation we apply Powell’s hybrid method as implemented in MINPACK wrapped through SciPy [67] to the equivalent real system of the two variables Re{λ} and Im{λ}.

Appropriate initial approximations ${\tilde{\lambda }}_{n}$ can be achieved (i) by exploiting that for sufficiently small generic mean input *μ*_min_ all eigenvalues have zero imaginary part (see Sect. *Remarks on the spectrum*). In that case the eigenvalues are given by the roots {λ_0_, λ_1_, …} of *q*(*V*_lb_; λ)–the one-dimensional function of the real-valued eigenvalue candidate λ ∈ (−∞, 0]–which are obtained, for example, by (dense) evaluation of that function in a sufficiently large (sub)interval below zero. Furthermore (ii) all eigenvalues depend continuously on the (input) parameters *μ* and *σ* (cf. Fig 7A), i.e., for a small step in the respective parameter space the solution λ_*n*_ of Eq (69) for the last parametrization is a very good initial value ${\tilde{\lambda }}_{n}$ for the current parametrization. With this initialization the solution of Eq (69) is typically found in a few steps of the chosen Newton-like method (see S1 Text for details).

To efficiently and accurately evaluate *q*_*ϕ*_(*V*_lb_; λ) in each iteration (of the root finding algorithm), we perform an exponential (backward) integration of the System (68). The resulting scheme is based on truncating the Magnus expansion of the exact solution after one term [71]. Here matrix exponential function evaluations of the form exp[**A**(*V*)Δ*V*] occur that are calculated using an analytic expression [72]. Note that ODE solvers that either do not exploit the linear structure of Eq (68) at all (such as the explicit Euler method but also higher order Runge-Kutta methods) or utilize linearity only in one variable (e.g., [73]) have poor convergence behaviour and thus require very small step sizes Δ*V* due to the strong nonlinearity *g*(*V*) as a consequence of the large value of the current *I*_exp_ close to *V*_s_ and significantly large absolute eigenvalues |λ_*n*_|, respectively.

For more information on the numerical solver we refer to S1 Text, where details regarding the adjoint operator, the exponential integration, the initial eigenvalue determination and the (parallelized) treatment of the input parameters are included.

### Cascade models

Linear-Nonlinear (LN) cascade models of neuronal activity are often applied in neuroscience, because they are simple and efficient, and the model components can be estimated using established experimental procedures [21, 74, 75]. Here we use the LN cascade as an ansatz to develop a low-dimensional model and we determine its components from the underlying Fokker-Planck model. This section builds upon [21] and extends that approach for recurrently coupled aEIF neurons; specifically, by taking into account an adaptation current and variations of the input variance. Furthermore, we consider an improved approximation of the derived linear filters and include an (optional) explicit description of the spike shape, cf. [23] (ch 4.2).

The cascade models considered here produce spike rate output by applying to the time-varying mean *μ*_syn_ and standard deviation *σ*_syn_ of the (overall) synaptic input, cf. Eq (21), separately a linear temporal filter, *D*_*μ*_ and *D*_*σ*_, followed by a common nonlinear function *F*. That is,

$$r\left(t\right)=F\;(\mu_{f},\sigma_{f},\left\langle w\right\rangle ),$$ (70)

$$\mu_{f}\left(t\right)=D_{\mu }*\mu_{\text{syn}}\left(t\right),$$ (71)

$$\sigma_{f}\left(t\right)=D_{\sigma }*\sigma_{\text{syn}}\left(t\right),$$ (72)

where *μ*_f_ and *σ*_f_ denote the filtered mean and filtered standard deviation of the input, respectively. $D_{\mu }*\mu_{\text{syn}}\left(t\right)=\int_{0}^{\infty }D_{\mu }\left(\tau \right)\mu_{\text{syn}}\left(t-\tau \right)d\tau$ is the convolution between *D*_*μ*_ and *μ*_syn_. The filters *D*_*μ*_, *D*_*σ*_ are adaptive in the sense that they depend on the mean adaptation current 〈*w*〉 and on the (arbitrary) baseline input in terms of baseline mean $\mu_{\text{syn}}^{0}$ and standard deviation $\sigma_{\text{syn}}^{0}$. For improved readability these dependencies are not explicitly indicated in Eqs (71) and (72). Note, that the nonlinearity *F* also depends on 〈*w*〉, which is governed by Eq (23). Since the mean adaptation current depends on the mean membrane voltage 〈*V*〉 we also consider a nonlinear mapping *H* for that population output quantity,

$$\left\langle V\right\rangle \left(t\right)=H\;(\mu_{f},\sigma_{f},\left\langle w\right\rangle ).$$ (73)

For the derivation below it is instructive to first consider an uncoupled population, i.e., the input moments do not depend on *r*_*d*_ for now. In particular, the input statistics are described by $\mu_{\text{syn}}\left(t\right)=\mu_{\text{syn}}^{0}+\mu_{\text{syn}}^{1}\left(t\right)$ and $\sigma_{\text{syn}}\left(t\right)=\sigma_{\text{syn}}^{0}+\sigma_{\text{syn}}^{1}\left(t\right)$. In the following, we derive the components *F*, *D*_*μ*_ and *D*_*σ*_ from the Fokker-Planck model for small amplitude variations $\mu_{\text{syn}}^{1}$, $\sigma_{\text{syn}}^{1}$ and for a slowly varying adaptation current (as already assumed). We then approximate the derived linear filter components using suitable functions such that the convolutions can be expressed in terms of simple ODEs. Finally, we account for time-varying baseline input ($\mu_{\text{syn}}^{0}\left(t\right)$, $\sigma_{\text{syn}}^{0}\left(t\right)$) and for recurrent coupling in the resulting low-dimensional spike rate models.

#### Deriving the components of the cascade

We first expand *F* in Eq (70) around the baseline $\mu_{f}=\mu_{\text{syn}}^{0}$, $\sigma_{f}=\sigma_{\text{syn}}^{0}$, 〈*w*〉 = 〈*w*〉_0_ to linear order, assuming that the amplitudes of $\mu_{\text{syn}}^{1}$ and $\sigma_{\text{syn}}^{1}$ are small, and the mean adaptation current varies slowly compared to the input moments, to obtain the approximation for Eq (70),

$$\begin{array}{ccc} r\left(t\right)\approx F\left(\mu_{\text{syn}}^{0},\sigma_{\text{syn}}^{0},\left\langle w\right\rangle \right) & + & D_{\mu }*\mu_{\text{syn}}^{1}\left(t\right)\;\frac{\partial }{\partial \mu }F\left(\mu_{\text{syn}}^{0},\sigma_{\text{syn}}^{0},\left\langle w\right\rangle \right)\\ & + & D_{\sigma }*\sigma_{\text{syn}}^{1}\left(t\right)\;\frac{\partial }{\partial \sigma }F\left(\mu_{\text{syn}}^{0},\sigma_{\text{syn}}^{0},\left\langle w\right\rangle \right). \end{array}$$ (74)

Due to slow adaptation (〈*w*〉(*t*) = 〈*w*〉_0_ + 〈*w*〉_1_(*t*) with vanishing 〈*w*〉_1_) we have neglected the expansion term in the direction of 〈*w*〉 and replaced 〈*w*〉_0_ = 〈*w*〉 in the approximation above. Note also that, without loss of generality, we have assumed normalized filters, $\int_{0}^{\infty }D_{\mu }\left(\tau \right)d\tau =\int_{0}^{\infty }D_{\sigma }\left(\tau \right)d\tau =1$. Under the same assumptions the output from the Fokker-Planck model (Eqs (23)–(32)) can be approximated as

$$r\left(t\right)\approx r_{\infty }(\mu_{\text{tot}}^{0},\sigma_{\text{tot}}^{0})+R_{\mu }*\mu_{\text{syn}}^{1}\left(t\right)+R_{\sigma }*\sigma_{\text{syn}}^{1}\left(t\right),$$ (75)

$$\frac{d\left\langle w\right\rangle }{dt}\approx \frac{a({\left\langle V\right\rangle }_{\infty }-E_{w})-\left\langle w\right\rangle }{\tau_{w}}+b\;r\left(t\right),$$ (76)

$$\mu_{\text{tot}}^{0}\left(t\right)=\mu_{\text{syn}}^{0}-\left\langle w\right\rangle /C,\;\sigma_{\text{tot}}^{0}=\sigma_{\text{syn}}^{0},$$ (77)

where *r*_∞_ and 〈*V*〉_∞_ are the steady-state spike rate and mean membrane voltage of a population of EIF neurons in response to an input of *total* mean $\mu_{\text{tot}}^{0}$ plus Gaussian white noise with standard deviation $\sigma_{\text{tot}}^{0}$. In particular, 〈*V*〉_∞_ reflects the mean over all nonrefractory neurons, cf. Eq (38). *R*_*μ*_ and *R*_*σ*_ are the so-called linear rate response functions of the population for weak modulations of the input mean and standard deviation around $\mu_{\text{tot}}^{0}$ and $\sigma_{\text{tot}}^{0}$, respectively [21, 73, 76]. Comparing Eqs (74) and (75) we obtain for the nonlinearity *F* and the linear filters *D*_*μ*_, *D*_*σ*_,

$$F\left(\mu ,\sigma ,\left\langle w\right\rangle \right)=r_{\infty }\;(\mu -\langle w\rangle /C,\sigma ),$$ (78)

$$D_{\mu }\left(t\right)=\frac{R_{\mu }\left(t\right)}{\frac{\partial }{\partial \mu }r_{\infty }\;(\mu_{\text{tot}}^{0},\sigma_{\text{tot}}^{0})},$$ (79)

$$D_{\sigma }\left(t\right)=\frac{R_{\sigma }\left(t\right)}{\frac{\partial }{\partial \sigma }r_{\infty }\;(\mu_{\text{tot}}^{0},\sigma_{\text{tot}}^{0})}.$$ (80)

Furthermore, the function *H* in Eq (73) is given by

$$H\left(\mu ,\sigma ,\left\langle w\right\rangle \right)={\left\langle V\right\rangle }_{\infty }\;(\mu -\langle w\rangle /C,\sigma ),$$ (81)

ensuring that the mean membrane voltage corresponds with the instantaneous spike rate estimate of the model (in every time step). Note that *R*_*μ*_ and *R*_*σ*_ depend on $\mu_{\text{tot}}^{0}$ and $\sigma_{\text{tot}}^{0}$ (which is again not explicitly indicated for improved readability).

Fortunately, the quantities *r*_∞_, 〈*V*〉_∞_, *R*_*μ*_, and *R*_*σ*_ can be calculated from the Fokker-Planck equation using an efficient numerical method [73]. In particular, for *r*_∞_ and 〈*V*〉_∞_ we need to solve a linear boundary value problem (BVP), and *R*_*μ*_(*t*), *R*_*σ*_(*t*) are calculated in the Fourier domain, where we need to solve two linear BVPs to obtain ${\hat{R}}_{\mu }\left(f\right)$, ${\hat{R}}_{\sigma }\left(f\right)$ for each frequency *f*. It is worth noting that the refractory period is included in a straightforward way and does not increase the complexity of the BVPs to be solved, see [23, 73] (and our provided code).

#### Approximating the filter components

**Fig 8:**
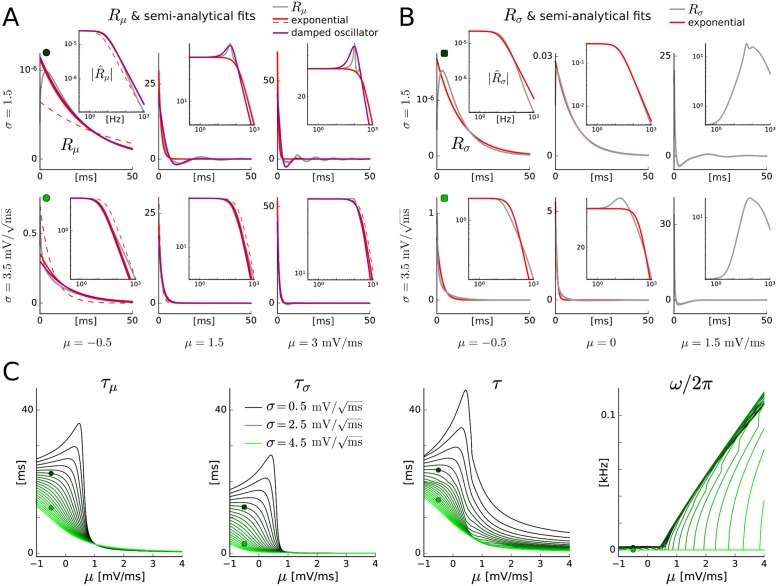
Linear rate response functions and quantities for the cascade models. Linear rate response functions of EIF neurons subject to white noise input for modulations of the input mean around *μ* with constant *σ*: *R*_*μ*_(*t*; *μ*, *σ*) in kHz/V (**A**, gray) and for modulations of the input standard deviation around *σ* with constant *μ*: *R*_*σ*_(*t*; *μ*, *σ*) in $\text{kHz}/(\text{V}\cdot \sqrt{\text{ms}}$) (**B**, gray). These functions are calculated in the Fourier domain for a range of modulation frequencies [${\hat{R}}_{\mu }\left(f;\mu ,\sigma \right)$ in 1/V and ${\hat{R}}_{\sigma }\left(f;\mu ,\sigma \right)$ in $1/(\text{V}\cdot \sqrt{\text{ms}}$)] (insets, gray; absolute values are shown), and fit using an exponential function exploiting asymptotic results for ${\hat{R}}_{\mu }$ (**A**, red dashed), as well as considering a range of frequencies (**A** and **B**, red solid). In addition, ${\hat{R}}_{\mu }$ is fit using a damped oscillator function (**A**, violet). The details of the fitting procedures are described in the text. **C**: quantities (*τ*_*μ*_, *τ*_*σ*_, *τ* and *ω*) from the linear filter approximations (cf. **A**, **B**), required for the LN_exp_ and LN_dos_ model variants (Eqs (85), (87) and (89)), as a function of *μ* and *σ*.

To express the LN model with adaptation, Eqs (70)–(73), (23) and (78)–(81), in terms of a low-dimensional ODE system we next approximate the linear filters *D*_*μ*_ and *D*_*σ*_ using suitable functions. The shapes of the true filters (proportional to *R*_*μ*_ and *R*_*σ*_, cf. Eqs (79) and (80)) for different input parameter values ($\mu =\mu_{\text{tot}}^{0}$, $\sigma =\sigma_{\text{tot}}^{0}$) are shown in Fig 8A and 8B.

We first consider the linear filter *D*_*μ*_ (Eq (79)) and apply the approximation

$$D_{\mu }\left(t\right)\approx A_{\mu }\exp(-t/\tau_{\mu }),$$ (82)

motivated by the exponential decay exhibited by *R*_*μ*_, particularly for large input variance compared to its mean. Note that *D*_*μ*_ depends on $\mu_{\text{tot}}^{0}$, $\sigma_{\text{tot}}^{0}$ and therefore the scaling parameter *A*_*μ*_ and the time constant *τ*_*μ*_ both depend on $\mu_{\text{tot}}^{0}$, $\sigma_{\text{tot}}^{0}$, which is not explicitly indicated. *A*_*μ*_ and *τ*_*μ*_ may be determined analytically using asymptotic results for the Fourier transform ${\hat{R}}_{\mu }$ of the linear rate response for vanishing and very large frequencies, respectively [21, 76],

$$\lim_{f\to 0}{\hat{R}}_{\mu }\left(f\right)=\frac{\partial }{\partial \mu }r_{\infty }\;(\mu_{\text{tot}}^{0},\sigma_{\text{tot}}^{0}),\;\lim_{f\to \infty }\;{\hat{R}}_{\mu }\left(f\right)=\frac{r_{\infty }\;(\mu_{\text{tot}}^{0},\sigma_{\text{tot}}^{0})}{i2\pi f\Delta_{T}}.$$ (83)

To guarantee that these asymptotics are matched by the Fourier transform *A*_*μ*_*τ*_*μ*_/(1 + *i*2*πfτ*_*μ*_) of the exponential, taking into account the scaling factor in Eq (79), we obtain

$$A_{\mu }=\frac{1}{\tau_{\mu }},\;\tau_{\mu }=\frac{\Delta_{T}}{r_{\infty }\;(\mu_{\text{tot}}^{0},\sigma_{\text{tot}}^{0})}\frac{\partial }{\partial \mu }r_{\infty }\;(\mu_{\text{tot}}^{0},\sigma_{\text{tot}}^{0}).$$ (84)

Note that matching the zero frequency limit in the Fourier domain is equivalent to the natural requirement that the time integral $\int_{0}^{\infty }D_{\mu }\left(\tau \right)d\tau$ of the linear filter is reproduced exactly, that is, the approximation is normalized appropriately. An advantage of this approximation is that it is no longer required to calculate the linear rate response function *R*_*μ*_ explicitly. On the other hand, as only the limit *f* → ∞ is used for fitting in addition to the normalization constraint, the approximation of the linear filter can be poor for a range of intermediate frequencies (in the Fourier domain), in particular for small input mean and standard deviation (see Fig 8A here, and Fig. 4B,C in [21]). To improve the approximation for intermediate frequencies we use the same normalization condition, which fixes the parameter *A*_*μ*_ = 1/*τ*_*μ*_, and we determine *τ*_*μ*_ by a least-squares fit of ${\hat{D}}_{\mu }$ over the range of frequencies *f* ∈ [0, 1] kHz. In both cases, using the approximation Eq (82) the filtered mean input *μ*_f_(*t*) = *D*_*μ*_ * *μ*_syn_(*t*) can be equivalently obtained by solving the simple scalar ODE,

$$\frac{d\mu_{f}}{dt}=\frac{\mu_{\text{syn}}-\mu_{f}}{\tau_{\mu }}.$$ (85)

Recall that *τ*_*μ*_ depends on $\mu_{\text{tot}}^{0}$, $\sigma_{\text{tot}}^{0}$. This exponentially decaying filter is part of the LN_exp_ cascade model variant.

A shortcoming of the approximation Eqs (82) and (85) above is that it cannot reproduce damped oscillations exhibited by the true linear filter, in particular, for large input mean and small variance (see Fig 8A). Therefore, we introduce an alternative approximation using a damped oscillator function,

$$D_{\mu }\left(t\right)\approx B_{\mu }\exp\left(-t/\tau \right)\cos\left(\omega t\right).$$ (86)

Note that here *B*_*μ*_, *τ* and *ω* depend on $\mu_{\text{tot}}^{0}$, $\sigma_{\text{tot}}^{0}$, which is not explicitly indicated. We fix the scaling parameter *B*_*μ*_ by (again) requiring that the approximation is normalized to reproduce the time integral of the true linear filter *D*_*μ*_, which yields *B*_*μ*_ = (1 + (*τ*^2^ *ω*^2^))/*τ*. The remaining two parameters *τ* and *ω* are determined such that the dominant oscillation frequency is reproduced. Specifically, the approximation should match ${\hat{D}}_{\mu }$ at the frequencies $f_{R}={\text{argmax}}_{f}\;|\text{Re}\{{\hat{D}}_{\mu }\left(f\right)\}|$ and $f_{I}={\text{argmax}}_{f}\;|\text{Im}\{{\hat{D}}_{\mu }\left(f\right)\}|$ in the Fourier domain as close as possible. We would like to note that using the method of least-squares over a range of frequencies instead can generate approximated filters which decay to zero instantly, particularly for large input mean and small variance (not shown). For such inputs a damped oscillator with a single decay time constant is too simple to fit the complete, rather complex linear filter shape. With Eq (86) the filtered mean input can be obtained by solving the second order ODE

$${\ddot{\mu }}_{f}+\frac{2}{\tau }{\dot{\mu }}_{f}+(\frac{2}{\tau^{2}}+\omega^{2})\;\mu_{f}=\frac{1+\tau^{2}\omega^{2}}{\tau }\;(\frac{\mu_{\text{syn}}}{\tau }+{\dot{\mu }}_{\text{syn}}).$$ (87)

Using this damped oscillator filter gives rise to the LN_dos_ cascade model variant.

Considering the linear filter *D*_*σ*_ (Eq (80)) we use the approximation *D*_*σ*_(*t*) ≈ *A*_*σ*_ exp(−*t*/*τ*_*σ*_), with *A*_*σ*_ = 1/*τ*_*σ*_ and *τ*_*σ*_ determined by a least-squares fit of ${\hat{D}}_{\sigma }$ for frequencies *f* ∈ [0, 1] kHz, as long as $\frac{\partial }{\partial \sigma }r_{\infty }\;(\mu_{\text{tot}}^{0},\sigma_{\text{tot}}^{0})>0$. When this condition is not fulfilled, which occurs for large input mean and small variance, the full linear filter cannot be properly fit by an exponential function. This may be seen by the asymptotic behavior [77]

$$\lim_{f\to \infty }{\hat{R}}_{\sigma }\left(f\right)=\frac{\sigma_{\text{tot}}^{0}r_{\infty }\;(\mu_{\text{tot}}^{0},\sigma_{\text{tot}}^{0})}{i2\pi f\Delta_{T}^{2}},$$ (88)

which implies negative $\lim_{f\to \infty }{\hat{D}}_{\sigma }\left(f\right)$ (cf. Eq (80)) that cannot be approximated for nonnegative *τ*_*σ*_. In this case we use *τ*_*σ*_ → 0 which effectively yields *D*_*σ*_(*t*) ≈ *δ*(*t*), justified by the observation that the full filter rapidly relaxes to zero (see Fig 8B). The linear filter application can be implemented by solving

$$\frac{d\sigma_{f}}{dt}=\frac{\sigma_{\text{syn}}-\sigma_{f}}{\tau_{\sigma }},$$ (89)

where *σ*_f_(*t*) = *D*_*σ*_ * *σ*_syn_(*t*) is the filtered input standard deviation. This filter is used in both model variants (LN_exp_ and LN_dos_). Note (again) that *τ*_*σ*_ depends on $\mu_{\text{tot}}^{0}$, $\sigma_{\text{tot}}^{0}$.

#### Extension for changing input baseline and recurrent coupling

In the derivation above we considered that the synaptic input mean and standard deviation, *μ*_syn_(*t*) and *σ*_syn_(*t*), vary around $\mu_{\text{syn}}^{0}$ and $\sigma_{\text{syn}}^{0}$ with small magnitudes. To extend the LN cascade model(s) to inputs that show large deviations from their baseline values we let the linear filters adjust to a changing input baseline in the following way: using the exponentially decaying mean input filter (LN_exp_ model) the filter parameters *τ*_*μ*_ and *τ*_*σ*_ are evaluated at

$$\mu_{\text{eff}}\left(t\right)=\mu_{f}-\left\langle w\right\rangle \left(t\right)/C,\;\sigma_{\text{eff}}\left(t\right)=\sigma_{f}$$ (90)

in every time step, i.e., these parameters adapt to the *effective* input moments. Using the damped oscillating mean input filter (LN_dos_ model), on the other hand, the filter parameters *τ*, *ω* and *τ*_*σ*_ are evaluated at *μ*_tot_(*t*), *σ*_tot_(*t*) given by Eqs (26) and (27), i.e., these parameters adapt directly to the *total* input moments, assuming that these moments do not fluctuate too vigorously. Note that because of these adjustments we do not need to consider a (particular) input baseline. Two remarks are in place: (i) the parameters of the damped oscillator cannot be adapted to a changing input baseline using the *effective* input mean (with *μ*_f_ given by Eq (87)), because this can lead to stable oscillations (for an uncoupled population) and thus decreased reproduction performance (not shown); (ii) for input moments that change very rapidly the reproduction performance of the LN_dos_ model variant may be improved by alternatively evaluating the parameters *τ*, *ω* and *τ*_*σ*_ at $\mu_{a}\left(t\right)=\mu_{f}^{a}-\langle w\rangle /C$, *σ*_eff_(*t*), with $\mu_{f}^{a}$ governed by Eq (85) (combining LN_exp_ and LN_dos_), cf. [23] (ch. 4.2).

Finally, recurrent coupling within the population is included (in both model variants) by the dependence of the synaptic input moments on the delayed spike rate, *μ*_syn_(*t*, *r*_*d*_), $\sigma_{\text{syn}}^{2}\left(t,r_{d}\right)$, with *r*_*d*_ given by Eq (40). For the LN_dos_ model we can then replace ${\dot{\mu }}_{\text{syn}}$ in Eq (87) by

$${\dot{\mu }}_{\text{syn}}={\dot{\mu }}_{\text{ext}}+JK{\dot{r}}_{d}={\dot{\mu }}_{\text{ext}}+JK\frac{r-r_{d}}{\tau_{d}}.$$ (91)

In case of identical (constant) propagation delays within the population this term would be ${\dot{\mu }}_{\text{syn}}={\dot{\mu }}_{\text{ext}}+JK\dot{r}\left(t-d\right)$ and in case of recurrent coupling without delays we would have

$${\dot{\mu }}_{\text{syn}}={\dot{\mu }}_{\text{ext}}+JK\;[\frac{\partial \;r_{\infty }}{\partial \mu }\;({\dot{\mu }}_{f}-\frac{a({\left\langle V\right\rangle }_{\infty }-E_{w})-\left\langle w\right\rangle }{C\tau_{w}}+\frac{b\;r}{C})+\frac{\partial \;r_{\infty }}{\partial \sigma }\frac{\sigma_{\text{syn}}-\sigma_{f}}{\tau_{\sigma }}].$$ (92)

To summarize both LN cascade models, the population spike rate and mean membrane voltage are described by Eqs (70) and (73), using Eqs (78), (81) and (23), respectively. For the LN_exp_ model input filtering is governed by Eqs (85) and (89), where the filter parameters are evaluated at *μ*_eff_(*t*), *σ*_eff_(*t*) (Eq (90)). For the LN_dos_ model input filtering is described by Eqs (87) and (89), where the filter parameters are evaluated at *μ*_tot_(*t*), *σ*_tot_(*t*) given by Eqs (26) and (27). The system for the recurrent network under consideration is closed by Eqs (21) and (40) which relate population spike rate output and overall synaptic input moments. Note that both LN systems here are fully equivalent to the respective ones specified in the Sect. *Model reduction*.

#### Further remarks

In order to efficiently simulate the derived cascade rate models it is highly recommended to precalculate the quantities which are needed in each time step on a (*μ*, *σ*)-rectangle (see Sect. *Low-Dimensional Approximations* above). For the LN_exp_ model these quantities are the filter time constants *τ*_*μ*_, *τ*_*σ*_, and for the LN_dos_ model we need the quantities *τ*, *ω* and *τ*_*σ*_ (all displayed in Fig 8C). Both models additionally require the steady-state quantities *r*_∞_ and 〈*V*〉_∞_ shown in Fig 6. An efficient implementation to obtain these quantities using Python with the package Numba for low-level virtual machine acceleration is available. Recall, that changing any parameter value of the external input, the recurrent synaptic input or the adaptation current does not require renewed precomputations.

If desired, it is also possible to obtain initial values for the variables of the cascade models (LN_exp_ and LN_dos_ variants) that correspond to a given initial distribution of membrane voltage and adaptation current values {*V*_*i*_(0)}, {*w*_*i*_(0)} of a population of *N* aEIF neurons. We can calculate 〈*w*〉(0) = 1/*N*∑_*i*_ *w*_*i*_(0) and determine *μ*_f_(0), *σ*_f_(0) by requiring that the initial membrane voltage distribution of the respective LN model *p*_∞_(*V*; *μ*_f_(0) − 〈*w*〉(0)/*C*, *σ*_f_(0)) matches the initial voltage distribution from the aEIF population as close as possible (e.g., using the Kolmogorov–Smirnov statistic). For the LN_dos_ model we additionally set ${\dot{\mu }}_{f}\left(0\right)=0$. This means we assume vanishing changes in the input history which underlies the initial membrane voltage distribution and filter parameters in the LN models (i.e., ${\dot{\mu }}_{\text{syn}}\approx 0$, ${\dot{\sigma }}_{\text{syn}}\approx 0$ for a sufficiently long time interval prior to *t* = 0).

The components of the LN model are derived in the limit of small amplitude variations of *μ*_syn_ and *σ*_syn_. However, the approximation also provides an exact description of the population dynamics for very slow variations of *μ*_syn_ and *σ*_syn_, where the spike rate, mean membrane voltage and adaptation current are well approximated by their steady-state values in each time step.

Here we approximated the derived linear filters using exponential and damped oscillator functions. We would like to note that, for a given baseline input ($\mu_{\text{tot}}^{0}$, $\sigma_{\text{tot}}^{0}$) the filter application using the latter function (Eq (86)) can be equivalently described by a complex-valued ODE [23] (ch. 4.2). Furthermore, the true linear filters *D*_*μ*_ and *D*_*σ*_ can be substantially better approximated by a damped oscillator function with two time scales (i.e., two exponentials) each. In these three cases, however, the ODE representation for the filter application can lead to decreased reproduction performance when the baseline input changes very rapidly (due to increased sensitivity to variations of the filter parameters).

### Spike shape extension (optional)

In this contribution the membrane voltage spike shape has been neglected (typical for IF type neuron models) by clamping *V*_*i*_ and *w*_*i*_ during the refractory period, justified by the observation that it is rather stereotyped and its duration is very brief. Furthermore, the spike shape is believed to contain little information compared to the time at which the spike occurs. Nevertheless, it can be incorporated in the aEIF model in a straightforward way using the following reset condition, as suggested in [43]: When *V*_*i*_ reaches the spike voltage *V*_s_ from below we let *V*_*i*_ decrease linearly from *V*_s_ to *V*_r_ during the refractory period and increment the adaptation current *w*_*i*_ ← *w*_*i*_ + *b* at the onset of that period. That is, *V*_*i*_ and *w*_*i*_ are not clamped during the refractory period, instead, *V*_*i*_ has a fixed time course and *w*_*i*_ is incremented by *b* and then governed again by Eq (15). This modification implies that the average membrane voltage in Eq (23) needs to be calculated over all neurons (and not only the nonrefractory ones), that is, 〈*V*〉 is calculated with respect to *p* + *p*_ref_, where $p_{\text{ref}}\left(V,t\right)=\int_{0}^{T_{\text{ref}}}r\left(t-s\right)\delta (V-V_{\text{sp}}\left(s\right))ds$ with spike trajectory *V*_*sp*_(*t*) = *V*_s_ + (*V*_r_ − *V*_s_)*t*/*T*_ref_, cf. [43]. The same applies to the steady-state mean membrane potential in Eqs (1), (39) and (76), i.e., 〈*V*〉_∞_ is then given by

$${\left\langle V\right\rangle }_{\infty }=\int_{-\infty }^{V_{s}}vp_{\infty }\left(v\right)dv+(1-\int_{-\infty }^{V_{s}}p_{\infty }\left(v\right)dv)\;\frac{V_{r}+V_{s}}{2},$$ (93)

instead of Eq (38). Notably, the accuracy of the adiabatic approximation (Eq (15)) does not depend on the refractory period *T*_ref_ in this case. That type of spike shape can therefore be considered in the FP model and the low-dimensional models in a straightforward way without significant additional computational demand. Note, however, that for the spec_2_ model a nonzero refractory period is not supported (see above). For an evaluation of the spike shape extension in terms of reproduction accuracy of the LN models see [23] (Fig. 4.15 in [23]).

## Supporting information

S1 Text Supplementary methods. A) Numerical integration of the time-dependent Fokker-Planck equation. B) Derivation of the model spec_2_ based on the Fokker-Planck operator. C) Numerical solver for the nonlinear Fokker-Planck eigenvalue problem.
(PDF)

S1 Fig Fast changes of the input variance. (PDF)
