# Supplementary material for: Low-dimensional spike rate models derived from networks of adaptive integrate-and-fire neurons: Comparison and implementation
Source: PLoS Comput Biol. 2017 Jun 23;13(6):e1005545. doi: 10.1371/journal.pcbi.1005545 (PMC5507472; doi:10.1371/journal.pcbi.1005545)
Supplement: S1 Text — A) Numerical integration of the time-dependent Fokker-Planck equation. B) Derivation of the model spec2 based on the Fokker-Planck operator. C) Numerical solver for the nonlinear Fokker-Planck eigenvalue problem. (PDF) [file pcbi.1005545.s001.pdf]

Low-dimensional spike rate models derived from networks of adaptive integrate-and-fire neurons: comparison and implementation

Moritz Augustin[1,2,*], Josef Ladenbauer[1,2,3,*], Fabian Baumann[1,2], Klaus Obermayer[1,2]

1 Department of Software Engineering and Theoretical Computer Science, Technische Universität Berlin, Germany
2 Bernstein Center for Computational Neuroscience Berlin, Germany
3 Group for Neural Theory, Laboratoire de Neurosciences Cognitives, École Normale Supérieure, Paris, France

*** augustin@ni.tu-berlin.de and josef.ladenbauer@tu-berlin.de**

S1 Text: Supplementary methods

A) Numerical integration of the time-dependent Fokker-Planck equation 2
A1) Numerical approach . . . 2
A2) Boundary conditions . . . 2
A3) Semi-implicit time discretization . . . 2

B) Derivation of the model spec$_2$ based on the Fokker-Planck operator 4
B1) Base model . . . 4
B2) Slowness and modal approximations . . . 5
B3) Compactification . . . 6
B4) Second order ordinary differential equation . . . 7
B5) Remarks . . . 9

C) Numerical solver for the nonlinear Fokker-Planck eigenvalue problem 10
C1) Problem statement . . . 10
C2) Parameter-dependent solution . . . 10
C3) Exponential integration . . . 11
C4) Adjoint operator . . . 12
C5) Quantities . . . 12
C6) Modifications . . . 13

A) Numerical integration of the time-dependent Fokker-Planck equation

A1) Numerical approach

We begin with three equations of the main text: Eq. (33),

$$\frac{\partial}{\partial t} p(V_m, t) = \frac{q_p(V_{m-\frac{1}{2}}, t) - q_p(V_{m+\frac{1}{2}}, t)}{\Delta V} + \delta_{m m_\mathrm{r}} \frac{1}{\Delta V} q_p(V_{N_V+\frac{1}{2}}, t - T_\mathrm{ref}), \qquad \text{(A-1)}$$

describes the membrane voltage discretization of the Fokker-Planck Eq. (24) and includes the reinjection condition, Eq. (30), for each grid cell $[V_{m-\frac{1}{2}}, V_{m+\frac{1}{2}}]$ with center V_m ($m = 1, \ldots, N_V$) and cell spacing ΔV. Secondly, Eq. (34),

$$q_p(V_{m+\frac{1}{2}}, t) = v_{m+\frac{1}{2}} \, \frac{p(V_m, t) - p(V_{m+1}, t) \, \exp(-v_{m+\frac{1}{2}} \Delta V/D)}{1 - \exp(-v_{m+\frac{1}{2}} \Delta V/D)}, \qquad \text{(A-2)}$$

represents the Scharfetter-Gummel flux approximation with drift and diffusion coefficients, $v_{m+\frac{1}{2}}$ and D, respectively. Lastly, Eq. (35),

$$(\mathbf{I} - \frac{\Delta t}{\Delta V} \mathbf{G}^n) \mathbf{p}^{n+1} = \mathbf{p}^n + \boldsymbol{g}^{n+1-n_\mathrm{ref}}, \qquad \text{(A-3)}$$

is the linear system after time discretization that is solved for $p_m^{n+1} = p(V_m, t_{n+1})$ in each timestep $t_n \to t_{n+1}$, where $g_m^{n+1-n_\mathrm{ref}} = \delta_{m m_\mathrm{r}} \frac{\Delta t}{\Delta V} r(t_{n+1-n_\mathrm{ref}})$ contains the flux reinjection. The coefficients of the system matrix $\mathbf{G}^n$ are obtained as follows.

A2) Boundary conditions

The absorbing boundary condition, $p(V_\mathrm{s}, t) = 0$ (cf. Eq. (31)), at cell border $V_{N_V+\frac{1}{2}} = V_\mathrm{s}$, is discretized through linear interpolation between the last cell N_V and a ghost cell that is introduced with center $V_{N_V+1} = V_\mathrm{s} + \Delta V/2$, yielding for the ghost value

$$p(V_{N_V+1}, t) = -p(V_{N_V}, t). \qquad \text{(A-4)}$$

The reflecting boundary cond. of Eq. (32), $q_p(V_\mathrm{lb}, t) = 0$, at cell border $V_{\frac{1}{2}} = V_\mathrm{lb}$ is discretized by inserting a second ghost cell with center $V_0 = V_\mathrm{lb} - \Delta V/2$ and by setting the flux in that cell, i.e., Eq. (A-2) for $m = 0$, to zero, which gives for the ghost value

$$p(V_0, t) = p(V_1, t) \exp(-v_{\frac{1}{2}} \Delta V/D). \qquad \text{(A-5)}$$

Furthermore, the spike rate, $r(t) = q_p(V_s, t)$, in this representation is given by evaluating the Scharfetter-Gummel flux, Eq. (A-2), at $V_{N_V+\frac{1}{2}}$, using the ghost value from the discretized absorbing boundary, Eq. (A-4), which yields

$$r(t) = q_p(V_{N_V+\frac{1}{2}}, t) = v_{N_V+\frac{1}{2}} \, \frac{1 + \exp(-v_{N_V+\frac{1}{2}} \Delta V/D)}{1 - \exp(-v_{N_V+\frac{1}{2}} \Delta V/D)} \, p(V_{N_V}, t), \qquad \text{(A-6)}$$

A3) Semi-implicit time discretization

Inserting the Scharfetter-Gummel flux representation, Eq. (A-2), into Eq. (A-1), using Eq. (A-6) (at $t - T_\mathrm{ref}$) and approximating the time derivative with first order

backward differences, results in

$$\begin{aligned}
\frac{p_m^{n+1} - p_m^n}{\Delta t} &= \frac{v_{m-\frac{1}{2}}}{\Delta V} \left(\frac{p_{m-1}^{n+1} - p_m^{n+1} \exp(-v_{m-\frac{1}{2}} \Delta V/D)}{1 - \exp(-v_{m-\frac{1}{2}} \Delta V/D)} \right) \\
&- \frac{v_{m+\frac{1}{2}}}{\Delta V} \left(\frac{p_m^{n+1} - p_{m+1}^{n+1} \exp(-v_{m+\frac{1}{2}} \Delta V/D)}{1 - \exp(-v_{m+\frac{1}{2}} \Delta V/D)} \right) \\
&+ \delta_{m m_\mathrm{r}} \frac{1}{\Delta V} r(t_{n+1-n_\mathrm{ref}}),
\end{aligned} \tag{A-7}$$

where the drift and diffusion coefficients, $v_{m\pm\frac{1}{2}}$ and D, respectively, are evaluated at t_n here and in the following, which corresponds more precisely to a semi-implicit time discretization.

Collecting terms for inner grid cells, $m = 2, \ldots, N_V - 1$, gives the following elements of the tridiagonal matrix $\mathbf{G}^n$ from Eq. (A-3):

$$\mathbf{G}^n_{m,m} = \frac{v_{m+\frac{1}{2}}}{\exp(-v_{m+\frac{1}{2}} \Delta V/D) - 1} + \frac{v_{m-\frac{1}{2}} \exp(-v_{m-\frac{1}{2}} \Delta V/D)}{\exp(-v_{m-\frac{1}{2}} \Delta V/D) - 1}, \tag{A-8}$$

$$\mathbf{G}^n_{m,m-1} = \frac{v_{m-\frac{1}{2}}}{1 - \exp(-v_{m-\frac{1}{2}} \Delta V/D)}, \tag{A-9}$$

$$\mathbf{G}^n_{m,m+1} = \frac{v_{m+\frac{1}{2}} \exp(-v_{m+\frac{1}{2}} \Delta V/D)}{1 - \exp(-v_{m+\frac{1}{2}} \Delta V/D)}. \tag{A-10}$$

The remaining nonzero elements, i.e., those in the first and last row of $\mathbf{G}^n$, are obtained by using the ghost cell values from the discretized boundary conditions. Inserting Eq. (A-5) into Eq. (A-7) with $m = 1$ yields for the reflecting boundary,

$$\mathbf{G}^n_{1,1} = \frac{v_{\frac{3}{2}}}{\exp(-v_{\frac{3}{2}} \Delta V/D) - 1}, \tag{A-11}$$

$$\mathbf{G}^n_{1,2} = \frac{v_{\frac{3}{2}} \exp(-v_{\frac{3}{2}} \Delta V/D)}{1 - \exp(-v_{\frac{3}{2}} \Delta V/D)}. \tag{A-12}$$

Note that these coefficients for the first row of $\mathbf{G}^n$ are alternatively also obtained by setting the term $q_p(V_{\frac{1}{2}}, t)$ to zero in Eq. (A-1) for $m = 1$ which allows to skip the introduction of the auxiliary ghost cell for the reflecting boundary.

For the absorbing boundary we insert Eq. (A-4) into Eq. (A-7) with $m = N_V$, resulting in

$$\mathbf{G}^n_{N_V, N_V-1} = \frac{v_{N_V-\frac{1}{2}}}{1 - \exp(-v_{N_V-\frac{1}{2}} \Delta V/D)} \tag{A-13}$$

$$\begin{aligned}
\mathbf{G}^n_{N_V, N_V} &= v_{N_V-\frac{1}{2}} \frac{\exp(-v_{N_V-\frac{1}{2}} \Delta V/D)}{\exp(-v_{N_V-\frac{1}{2}} \Delta V/D) - 1} \\
&+ v_{N_V+\frac{1}{2}} \frac{\exp(-v_{N_V+\frac{1}{2}} \Delta V/D) + 1}{\exp(-v_{N_V+\frac{1}{2}} \Delta V/D) - 1},
\end{aligned} \tag{A-14}$$

which completes the specification of the tridiagonal matrix $\mathbf{G}^n$ and thus the system, Eq. (A-3), that is, of Eq. (35).

B) Derivation of the model spec$_2$ based on the Fokker-Planck operator

B1) Base model

We start with the Fokker-Planck mean-field model in spectral representation, Eqs. (55), (56) and (39),

$$\dot{\boldsymbol{\alpha}} = \left(\boldsymbol{\Lambda} + \mathbf{C}_\mu \dot{\mu} + \mathbf{C}_{\sigma^2} \dot{\sigma^2}\right) \boldsymbol{\alpha} + \mathbf{c}_\mu \dot{\mu} + \mathbf{c}_{\sigma^2} \dot{\sigma^2} \tag{B-1}$$

$$r(t) = r_\infty + \mathbf{f} \cdot \boldsymbol{\alpha} \tag{B-2}$$

$$\dot{\langle w \rangle} = \frac{a\left(\langle V \rangle_\infty - E_w\right) - \langle w \rangle}{\tau_w} + b\, r, \tag{B-3}$$

for the (infinitely many) projection coefficients $(\alpha_1, \alpha_2, \dots)$, the population-averaged spike rate r and adaptation current $\langle w \rangle$, respectively. They depend on the quantities $\boldsymbol{\Lambda}$, $\mathbf{C}_x$, $\mathbf{c}_x$ (for $x \in \{\mu, \sigma^2\}$), r_∞, $\mathbf{f}$ and $\langle V \rangle_\infty$, which all are evaluated at the total input moments

$$\mu(t) = \overbrace{\mu_{\text{ext}}(t) + KJr_d(t)}^{=\mu_{\text{syn(t)}}} - \frac{\langle w \rangle(t)}{C}, \tag{B-4}$$

$$\sigma^2(t) = \sigma^2_{\text{ext}}(t) + KJ^2\, r_d(t) = \sigma^2_{\text{syn}}(t) \tag{B-5}$$

omitting subscripts $_{\text{tot}}$ here and in the following (cf. Eqs. (21),(26),(27)). Additionally, Eq. (B-1), contains the (first order) time derivative of the total input moments, $\dot{\mu}$, $\dot{\sigma^2}$.

For increased generality here we do not restrict the form of the delay distribution p_d (i.e., the delayed spike rate is given by $r_d = r * p_d$, cf. Eq. (20)) but show specific examples that include exponentially distributed, identical and no delays further below.

Deriving Eqs. (B-1) once and (B-2) twice w.r.t. time gives

$$\begin{aligned}
\ddot{\boldsymbol{\alpha}} =& \Big(\partial_\mu \boldsymbol{\Lambda} \dot{\mu} + \partial_{\sigma^2} \boldsymbol{\Lambda} \dot{\sigma^2} + \left[\partial_\mu \mathbf{C}_\mu \dot{\mu} + \partial_{\sigma^2} \mathbf{C}_\mu \dot{\sigma^2}\right] \dot{\mu} + \mathbf{C}_\mu \ddot{\mu} \\
&+ \left[\partial_\mu \mathbf{C}_{\sigma^2} \dot{\mu} + \partial_{\sigma^2} \mathbf{C}_{\sigma^2} \dot{\sigma^2}\right] \dot{\sigma^2} + \mathbf{C}_{\sigma^2} \ddot{\sigma^2}\Big) \boldsymbol{\alpha} \\
&+ \left(\boldsymbol{\Lambda} + \mathbf{C}_\mu \dot{\mu} + \mathbf{C}_{\sigma^2} \dot{\sigma^2}\right) \dot{\boldsymbol{\alpha}} + \partial_\mu \mathbf{c}_\mu (\dot{\mu})^2 + \mathbf{c}_\mu \ddot{\mu} + \partial_{\sigma^2} \mathbf{c}_\mu \dot{\sigma^2} \dot{\mu} \\
&+ \partial_\mu \mathbf{c}_{\sigma^2} \dot{\mu} \dot{\sigma^2} + \partial_{\sigma^2} \mathbf{c}_{\sigma^2} (\dot{\sigma^2})^2 + \mathbf{c}_{\sigma^2} \ddot{\sigma^2},
\end{aligned} \tag{B-6}$$

$$\dot{r} = \partial_\mu r_\infty \dot{\mu} + \partial_{\sigma^2} r_\infty \dot{\sigma^2} + \left(\partial_\mu \mathbf{f}\, \dot{\mu} + \partial_{\sigma^2} \mathbf{f}\, \dot{\sigma^2}\right) \cdot \boldsymbol{\alpha} + \mathbf{f} \cdot \dot{\boldsymbol{\alpha}}, \tag{B-7}$$

$$\begin{aligned}
\ddot{r} =& \left(\partial_{\mu\mu} r_\infty \dot{\mu} + \partial_{\sigma^2\mu} r_\infty \dot{\sigma^2}\right) \dot{\mu} + \partial_\mu r_\infty \ddot{\mu} \\
&+ \left(\partial_{\mu\sigma^2} r_\infty \dot{\mu} + \partial_{\sigma^2\sigma^2} r_\infty \dot{\sigma^2}\right) \dot{\sigma^2} + \partial_{\sigma^2} r_\infty \ddot{\sigma^2} \\
&+ \Big(\left[\partial_{\mu\mu} \mathbf{f}\, \dot{\mu} + \partial_{\sigma^2\mu} \mathbf{f}\, \dot{\sigma^2}\right] \dot{\mu} + \partial_\mu \mathbf{f}\, \ddot{\mu} \\
&\quad + \left[\partial_{\mu\sigma^2} \mathbf{f}\, \dot{\mu} + \partial_{\sigma^2\sigma^2} \mathbf{f}\, \dot{\sigma^2}\right] \dot{\sigma^2} + \partial_{\sigma^2} \mathbf{f}\, \ddot{\sigma^2}\Big) \cdot \boldsymbol{\alpha} \\
&+ 2\left(\partial_\mu \mathbf{f}\, \dot{\mu} + \partial_{\sigma^2} \mathbf{f}\, \dot{\sigma^2}\right) \cdot \dot{\boldsymbol{\alpha}} + \mathbf{f} \cdot \ddot{\boldsymbol{\alpha}}.
\end{aligned} \tag{B-8}$$

B2) Slowness and modal approximations

Assuming slowly changing total input moments, i.e., small input variations $\dot{\mu}$ and $\dot{\sigma^2}$ allows to consider projections coefficients of that order: $\alpha_n = \mathcal{O}(\dot{\mu})$ and $\alpha_n = \mathcal{O}(\dot{\sigma^2})$. We can therefore neglect all higher order terms and in particular those which are proportional to the following factors: $\dot{\mu}\dot{\sigma^2}$, $(\dot{\mu})^2$, $(\dot{\sigma^2})^2$, $\dot{\mu}\boldsymbol{\alpha}$, $\dot{\sigma^2}\boldsymbol{\alpha}$, $\ddot{\mu}\boldsymbol{\alpha}$, $\ddot{\sigma^2}\boldsymbol{\alpha}$, $\dot{\mu}\dot{\boldsymbol{\alpha}}$, $\dot{\sigma^2}\dot{\boldsymbol{\alpha}}$.

With this approximation Eqs. (B-1),(B-6)–(B-8) become

$$\dot{\boldsymbol{\alpha}} = \boldsymbol{\Lambda}\,\boldsymbol{\alpha} + \mathbf{c}_\mu \dot{\mu} + \mathbf{c}_{\sigma^2}\dot{\sigma^2}, \tag{B-9}$$

$$\ddot{\boldsymbol{\alpha}} = \underbrace{\boldsymbol{\Lambda}^2\,\boldsymbol{\alpha} + \boldsymbol{\Lambda}\,\mathbf{c}_\mu \dot{\mu} + \boldsymbol{\Lambda}\,\mathbf{c}_{\sigma^2}\dot{\sigma^2}}_{=\boldsymbol{\Lambda}\dot{\boldsymbol{\alpha}}} + \mathbf{c}_\mu \ddot{\mu} + \mathbf{c}_{\sigma^2}\ddot{\sigma^2}, \tag{B-10}$$

$$\dot{r} = \partial_\mu r_\infty \dot{\mu} + \partial_{\sigma^2} r_\infty \dot{\sigma^2} + \underbrace{\mathbf{f}\cdot\boldsymbol{\Lambda}\,\boldsymbol{\alpha} + \mathbf{f}\cdot\mathbf{c}_\mu\dot{\mu} + \mathbf{f}\cdot\mathbf{c}_{\sigma^2}\dot{\sigma^2}}_{=\mathbf{f}\cdot\dot{\boldsymbol{\alpha}}}, \tag{B-11}$$

$$\begin{aligned} \ddot{r} &= \partial_\mu r_\infty \ddot{\mu} + \partial_{\sigma^2} r_\infty \ddot{\sigma^2} \\ &\quad + \underbrace{\mathbf{f}\cdot\boldsymbol{\Lambda}^2\,\boldsymbol{\alpha} + \mathbf{f}\cdot\mathbf{c}_\mu\ddot{\mu} + \mathbf{f}\cdot\mathbf{c}_{\sigma^2}\ddot{\sigma^2} + \mathbf{f}\cdot\boldsymbol{\Lambda}\,\mathbf{c}_\mu\dot{\mu} + \mathbf{f}\cdot\boldsymbol{\Lambda}\,\mathbf{c}_{\sigma^2}\dot{\sigma^2}}_{=\mathbf{f}\cdot\ddot{\boldsymbol{\alpha}}}. \end{aligned} \tag{B-12}$$

We now consider only the first two dominant eigenvalues λ_1 and λ_2 (cf. Eqs. (58),(67)) and neglect all (faster) eigenmodes corresponding to eigenvalues with larger absolute real part ("modal approximation"). Therefore, we take into account only the first two components of the (originally infinite-dimensional) variables $\boldsymbol{\alpha} = (\alpha_1, \alpha_2)^T$ and quantities $\boldsymbol{\Lambda} = \mathrm{diag}(\lambda_1, \lambda_2)$, $\mathbf{f} = (f_1, f_2)^T$, $\mathbf{c}_\mu = (c_1^\mu, c_2^\mu)^T$, $\mathbf{c}_{\sigma^2} = (c_1^{\sigma^2}, c_2^{\sigma^2})^T$. Note that here and in the following the bold symbols denote the two-dimensional vectors and (diagonal) matrix, respectively.

Due to the modal approximation Eqs. (B-11),(B-12) form a linear, two-dimensional algebraic system with unknowns $f_1\alpha_1$ and $f_2\alpha_2$. Solving this problem and inserting the solution into Eq. (B-2) using $\mathbf{f}\cdot\boldsymbol{\alpha} = f_1\alpha_1 + f_2\alpha_2$ yields

$$\begin{aligned} r_\infty - r = &-\left(\frac{1}{\lambda_1} + \frac{1}{\lambda_2}\right)\left[\dot{r} - \partial_\mu r_\infty \dot{\mu} - \partial_{\sigma^2} r_\infty \dot{\sigma^2} - \mathbf{f}\cdot\mathbf{c}_\mu\dot{\mu} - \mathbf{f}\cdot\mathbf{c}_{\sigma^2}\dot{\sigma^2}\right] \\ &+ \frac{1}{\lambda_1\lambda_2}\Big[\ddot{r} - \partial_\mu r_\infty \ddot{\mu} - \partial_{\sigma^2} r_\infty \ddot{\sigma^2} - \mathbf{f}\cdot\mathbf{c}_\mu\ddot{\mu} - \mathbf{f}\cdot\mathbf{c}_{\sigma^2}\ddot{\sigma^2} \\ &\qquad - \mathbf{f}\cdot\boldsymbol{\Lambda}\,\mathbf{c}_\mu\dot{\mu} - \mathbf{f}\cdot\boldsymbol{\Lambda}\mathbf{c}_{\sigma^2}\dot{\sigma^2}\Big]. \end{aligned} \tag{B-13}$$

This Equation represents the complete spike rate dynamics under the modal and slowness approximations. It involves the first two time derivatives of the total input

moments,

$$\begin{aligned}
\dot{\mu} &= \dot{\mu}_{\text{ext}}(t) + KJ\dot{r}_d(t) - \frac{\dot{\langle w \rangle}}{C} \qquad \text{(B-14)}\\
&= \dot{\mu}_{\text{ext}}(t) + KJ\dot{r}_d(t) - \frac{a\left(\langle V\rangle_\infty - E_w\right) - \langle w\rangle}{\tau_w C} - \frac{br}{C},\\
\ddot{\mu} &= \ddot{\mu}_{\text{ext}}(t) + KJ\ddot{r}_d(t) - \frac{\ddot{\langle w \rangle}}{C}, \qquad \text{(B-15)}\\
&= \ddot{\mu}_{\text{ext}}(t) + KJ\ddot{r}_d(t) - \frac{a}{\tau_w C}\Big(\partial_\mu \langle V\rangle_\infty \dot{\mu} + \partial_{\sigma^2}\langle V\rangle_\infty \dot{\sigma^2}\Big)\\
&\quad + \frac{\dot{\langle w \rangle}}{\tau_w C} - \frac{b\dot{r}}{C},\\
&= \ddot{\mu}_{\text{ext}}(t) + KJ\ddot{r}_d(t) - \frac{a}{\tau_w C}\Big(\partial_\mu \langle V\rangle_\infty \dot{\mu} + \partial_{\sigma^2}\langle V\rangle_\infty \dot{\sigma^2}\Big) \qquad \text{(B-16)}\\
&\quad + \frac{a\left(\langle V\rangle_\infty - E_w\right) - \langle w\rangle}{\tau_w^2 C} + \frac{br}{\tau_w C} - \frac{b\dot{r}}{C},\\
\dot{\sigma^2} &= \dot{\sigma}^2_{\text{ext}}(t) + KJ^2\dot{r}_d(t), \qquad \text{(B-17)}\\
\ddot{\sigma^2} &= \ddot{\sigma}^2_{\text{ext}}(t) + KJ^2\ddot{r}_d(t). \qquad \text{(B-18)}
\end{aligned}$$

B3) Compactification

Expanding the terms of Eqs. (B-14)–(B-18) in Eq. (B-13) yields

$$\begin{aligned}
r_\infty - r =& - T\dot{r} + D\ddot{r}\\
&- D\left(\partial_\mu r_\infty + \mathbf{f}\cdot\mathbf{c}_\mu\right)\Bigg(\ddot{\mu}_{\text{ext}} + \frac{a\left(\langle V\rangle_\infty - E_w\right) - \langle w\rangle}{\tau_w^2 C}\\
&\qquad\qquad + KJ\ddot{r}_d + \frac{br}{\tau_w C} - \frac{b\dot{r}}{C}\Bigg)\\
&- D\big(\partial_{\sigma^2} r_\infty + \mathbf{f}\cdot\mathbf{c}_{\sigma^2}\big)\big(\ddot{\sigma}^2_{\text{ext}} + KJ^2\ddot{r}_d\big)\\
&+ \Bigg(\dot{\mu}_{\text{ext}} + KJ\dot{r}_d - \frac{a\left(\langle V\rangle_\infty - E_w\right) - \langle w\rangle}{\tau_w C} - \frac{br}{C}\Bigg) \qquad \text{(B-19)}\\
&+ \left(\dot{\sigma}^2_{\text{ext}} + KJ^2\dot{r}_d\right)\\
&\quad\cdot\Big[T\big(\partial_\mu r_\infty + \mathbf{f}\cdot\mathbf{c}_\mu\big) + D\big(\partial_\mu r_\infty + \mathbf{f}\cdot\mathbf{c}_\mu\big)\frac{a}{\tau_w C}\partial_\mu\langle V\rangle_\infty\\
&\qquad - D\mathbf{f}\cdot\mathbf{\Lambda}\,\mathbf{c}_\mu\Big]\\
&\quad\cdot\Big[T\big(\partial_{\sigma^2} r_\infty + \mathbf{f}\cdot\mathbf{c}_{\sigma^2}\big) + D\big(\partial_\mu r_\infty + \mathbf{f}\cdot\mathbf{c}_\mu\big)\frac{a}{\tau_w C}\partial_{\sigma^2}\langle V\rangle_\infty\\
&\qquad - D\mathbf{f}\cdot\mathbf{\Lambda}\,\mathbf{c}_{\sigma^2}\Big],
\end{aligned}$$

where we have introduced the trace T and determinant D of the inverse eigenvalue matrix $\mathbf{\Lambda}^{-1}$,

$$\begin{aligned}
T &= 1/\lambda_1 + 1/\lambda_2, \qquad \text{(B-20)}\\
D &= 1/\lambda_1 \cdot 1/\lambda_2, \qquad \text{(B-21)}
\end{aligned}$$

for simplicity. With the definitions of the additional (lumped) quantities,

$$
\begin{aligned}
F_\mu &= \mathbf{f} \cdot \mathbf{\Lambda}\, \mathbf{c}_\mu, & \text{(B-22)}\\
F_{\sigma^2} &= \mathbf{f} \cdot \mathbf{\Lambda}\, \mathbf{c}_{\sigma^2}, & \text{(B-23)}\\
M &= \partial_\mu r_\infty + \mathbf{f} \cdot \mathbf{c}_\mu, & \text{(B-24)}\\
S &= \partial_{\sigma^2} r_\infty + \mathbf{f} \cdot \mathbf{c}_{\sigma^2}, & \text{(B-25)}\\
R &= DMKJ + DSKJ^2, & \text{(B-26)}\\
H_\mu &= TM + DM\frac{a}{\tau_w C}\partial_\mu \langle V\rangle_\infty - DF_\mu, & \text{(B-27)}\\
H_{\sigma^2} &= TS + DM\frac{a}{\tau_w C}\partial_{\sigma^2} \langle V\rangle_\infty - DF_{\sigma^2}, & \text{(B-28)}
\end{aligned}
$$

that are composed of the individual quantities (and of neuron and coupling parameters), Eq. (B-19) can be rewritten as

$$
\begin{aligned}
r_\infty - r = {} & r\left(-DM\frac{b}{\tau_w C} - \frac{b}{C}H_\mu\right)\\
& + \dot{r}\left(-T + DM\frac{b}{C}\right)\\
& + \dot{r}_d\left(KJH_\mu + KJ^2 H_{\sigma^2}\right)\\
& + \ddot{r}D\\
& - \ddot{r}_d R\\
& - DM\left(\ddot{\mu}_{\text{ext}} + \frac{a\left(\langle V\rangle_\infty - E_w\right) - \langle w\rangle}{\tau_w^2 C}\right)\\
& + \left(\dot{\mu}_{\text{ext}} - \frac{a\left(\langle V\rangle_\infty - E_w\right) - \langle w\rangle}{\tau_w C}\right)H_\mu\\
& + \dot{\sigma}^2_{\text{ext}} H_{\sigma^2} - DS\ddot{\sigma}^2_{\text{ext}}.
\end{aligned}
\qquad \text{(B-29)}
$$

Note that the first six lumped quantities, Eqs. (B-20)–(B-25), depend on the parameters of recurrent coupling and adaptation current (only) via the total input moments, cf. Eq. (B-4),(B-5), while the last three, Eqs. (B-26)–(B-28), contain them (K, J, a, τ_w) explicitly. Those lumped quantities, i.e., R, H_μ, H_{σ^2}, can, for example, be evaluated during runtime for the respective total input moments by using precalculations of the other lumped quantities (T, D, F_μ, F_{σ^2}, M, S) and of the additional individual quantities $\partial_\mu \langle V\rangle_\infty$ and $\partial_{\sigma^2} \langle V\rangle_\infty$, that all are independent of adaptation and synaptic parameters.

B4) Second order ordinary differential equation

Eq. (B-29) leads to the final model (cf. Eq. (62)): a real-valued second order equation for the spike rate $r(t)$,

$$\beta_2\ddot{r} + \beta_1\dot{r} + \beta_0 r = r_\infty - r - \beta_c, \qquad \text{(B-30)}$$

with coefficients β_2, β_1, β_0 and β_c, that depend on the total input moments $\mu(t, r_d, \langle w\rangle)$ and $\sigma^2(t, r_d, \langle w\rangle)$ (cf. Eqs. (B-4),(B-5)). Their concrete form is determined by the delay distribution p_d. Particularly, we distinguish three cases: (i) coupling without delay, $p_d(\tau) = \delta(\tau)$, (ii) exponentially distributed delays, $p_d(\tau) = \exp(-\tau/\tau_d)/\tau_d$ (for $\tau \geq 0$), and (iii) identical delays, $p_d(\tau) = \delta(\tau - d)$ with $d > 0$.

Case i) – coupling without delays For instantaneous synaptic interaction we have $r = r_d$. Thus, the coefficients of Eq. (B-30) are obtained by direct comparison with Eq. (B-29) which gives

$$\beta_2 = D - R, \tag{B-31}$$

$$\beta_1 = -T + DM\frac{b}{C} + KJH_\mu + KJ^2 H_{\sigma^2}, \tag{B-32}$$

$$\beta_0 = -DM\frac{b}{\tau_w C} - \frac{b}{C}H_\mu, \tag{B-33}$$

$$\beta_c = -\left(\ddot{\mu}_{\text{ext}} + \frac{a\left(\langle V\rangle_\infty - E_w\right) - \langle w\rangle}{\tau_w^2 C}\right) DM - \ddot{\sigma}^2_{\text{ext}} DS, \tag{B-34}$$
$$+\left(\dot{\mu}_{\text{ext}} - \frac{a\left(\langle V\rangle_\infty - E_w\right) - \langle w\rangle}{\tau_w C}\right) H_\mu + \dot{\sigma}^2_{\text{ext}} H_{\sigma^2}.$$

Note that β_c depends explicitly on the population-averged adaptation current $\langle w\rangle$ as well as on the first and second order time derivatives of the external moments μ_{ext} and σ^2_{ext}.

Case ii) – exponentially distributed delays Here we obtain the delayed rate r_d by solving $\dot{r_d} = (r - r_d)/\tau_d$. Inserting this expression together with its time derivative into Eq. (B-29) results in the coefficients

$$\beta_2 = D, \tag{B-35}$$

$$\beta_1 = -T + DM\frac{b}{C} - \frac{R}{\tau_d}, \tag{B-36}$$

$$\beta_0 = -DM\frac{b}{\tau_w C} - \frac{b}{C}H_\mu + \frac{1}{\tau_d}\left(KJH_\mu + KJ^2 H_{\sigma^2}\right) + \frac{R}{\tau_d^2}, \tag{B-37}$$

$$\beta_c = r_d\left(-\frac{1}{\tau_d}\left(KJH_\mu + KJ^2 H_{\sigma^2}\right) - \frac{R}{\tau_d^2}\right), \tag{B-38}$$
$$-\left(\ddot{\mu}_{\text{ext}} + \frac{a\left(\langle V\rangle_\infty - E_w\right) - \langle w\rangle}{\tau_w^2 C}\right) DM - \ddot{\sigma}^2_{\text{ext}} DS$$
$$+\left(\dot{\mu}_{\text{ext}} - \frac{a\left(\langle V\rangle_\infty - E_w\right) - \langle w\rangle}{\tau_w C}\right) H_\mu + \dot{\sigma}^2_{\text{ext}} H_{\sigma^2},$$

that correspond to those in Eqs. (63)–(66). Here β_c depends explicitly on the delayed spike rate r_d (in addition to $\langle w\rangle$ as well as the first and second order time derivatives of μ_{ext} and σ^2_{ext} as in the case without delays).

Case iii) – identical delays The delayed spike rate in this situation is given by $r_d(t) = r(t - d)$. Inserting the first and second order time derivative of this identity into Eq. (B-29) yields

$$\beta_2 = D, \tag{B-39}$$

$$\beta_1 = -T + DM\frac{b}{C}, \tag{B-40}$$

$$\beta_0 = -DM\frac{b}{\tau_w C} - \frac{b}{C}H_\mu, \tag{B-41}$$

$$\begin{aligned}\beta_c = &- \ddot{r}(t-d)R + \dot{r}(t-d)\left(KJH_\mu + KJ^2 H_{\sigma^2}\right) \\ &- \left(\ddot{\mu}_{\text{ext}} + \frac{a\left(\langle V\rangle_\infty - E_w\right) - \langle w\rangle}{\tau_w^2 C}\right)DM - \ddot{\sigma}^2_{\text{ext}}DS \\ &+ \left(\dot{\mu}_{\text{ext}} - \frac{a\left(\langle V\rangle_\infty - E_w\right) - \langle w\rangle}{\tau_w C}\right)H_\mu + \dot{\sigma}^2_{\text{ext}}H_{\sigma^2},\end{aligned} \tag{B-42}$$

Here β_c depends explicitly on $\ddot{r}(t-d)$ and $\dot{r}(t-d)$ (in addition to $\langle w\rangle$ as well as the first and second order time derivatives of μ_{ext} and σ^2_{ext} as in the case without delays).

B5) Remarks

- Equation (B-30) is an ordinary differential spike rate model for the cases (i) and (ii), i.e., without or exponentially distributed delays, while for identical delays (case iii) delayed variables occur explicitly in β_c and due to $r_d(t) = r(t-d)$ also in any model quantity via the total input moments.

- For exponentially distributed delays with an identical shift d, i.e., $p_d(\tau) = \exp[-(\tau - d)/\tau_d]/\tau_d$ with $\tau \geq d$, the delayed spike rate r_d satisfies $\dot{r_d}(t) = [r(t-d) - r_d(t)]/\tau_d$. In this situation the coefficients (β_2, β_1 and β_0) are identical to those of case iii) except for β_c which is modified and depends on $r_d(t)$, $r(t-d)$, $\dot{r}(t-d)$ (and the parameter τ_d), i.e., here Eq. (B-30) also represents a delay differential model.

- Any delay distribution p_d from the exponential family can be incorporated similarly as for the specific instance of an exponentially distribution (cf. case ii) to yield (non-delayed) coefficients of Eq. (B-30) by using the equivalent representation of the delayed spike rate r_d as an ODE (system).

- The scenario of an uncoupled population is obtained from any of the three cases by setting the number of presynaptic neurons K to zero (implying $R = 0$).

- As an alternative to derive and simulate the explicit model, Eq. (B-30), one can directly integrate Eq. (B-13) numerically by replacing the first two time derivatives of the total moments μ and σ^2 by finite (backward) differences in each timestep. This approach avoids lengthy expressions and might be especially useful when considering multiple interacting populations irrespective of the delay distribution.

C) Numerical solver for the nonlinear Fokker-Planck eigenvalue problem

C1) Problem statement

The (main) objective is to find the eigenvalues λ_n of the Fokker-Planck operator $\mathcal{L}$ which are the solutions of the complex-valued Eq. (69),

$$\lambda \mapsto q_\phi(V_{\mathrm{lb}}; \lambda) \overset{!}{=} 0. \tag{C-1}$$

Each evaluation of $q_\phi(V_{\mathrm{lb}}; \lambda)$ involves a backward integration of Eq. (68),

$$-\frac{d}{dV}\begin{pmatrix} q_\phi \\ \phi \end{pmatrix} = \underbrace{\begin{pmatrix} 0 & \lambda \\ \frac{2}{\sigma^2} & -2\frac{g(V)+\mu}{\sigma^2} \end{pmatrix}}_{=\mathbf{A}} \begin{pmatrix} q_\phi \\ \phi \end{pmatrix}, \tag{C-2}$$

which is initialized according to the absorbing boundary condition (cf. Eq. (44)),

$$\phi(V_{\mathrm{s}}) = 0 \tag{C-3}$$

and the arbitrary choice $q_\phi(V_{\mathrm{s}}) \in \mathbb{C} \setminus \{0\}$ (due to the linearity of the problem), and which furthermore has to take into account the (generalized) reinjection condition, Eq. (60),

$$q_\phi(V_{\mathrm{r}}^-) = q_\phi(V_{\mathrm{r}}^+) - q_\phi(V_{\mathrm{s}}) e^{-\lambda T_{\mathrm{ref}}}. \tag{C-4}$$

Note that the latter corresponds for $T_{\mathrm{ref}} = 0$ to the reinjection condition that does not include the refractory period, i.e., Eq. (47).

C2) Parameter-dependent solution

The eigenvalues λ_n, the associated eigenfunctions $\phi_n(V)$ of $\mathcal{L}$ and $\psi_n(V)$ of $\mathcal{L}^*$ are required for a rectangle of input parameter values (μ, σ). Using the property the eigenvalues are real-valued for sufficiently small mean input μ and that they furthermore continuously depend on both (input) parameters, mean μ and standard deviation σ, we establish the following solution algorithm:

1. Discretize the input parameter rectangle, $\{(\mu_k, \sigma_\ell)\}$, $k = 1, \ldots, M_\mu$, $\ell = 1, \ldots, M_\sigma$ with small spacings $\Delta\mu$, $\Delta\sigma$.

2. For $\mu_1 = \mu_{\mathrm{min}}$ evaluate $q(V_{\mathrm{lb}}; \lambda)$ with high resolution on a real negative interval $[\lambda_{\mathrm{min}}, 0)$ with sufficiently small $\lambda_{\mathrm{min}} \ll 0$ such that at least N_λ eigenvalues are found. The zero-crossings of $q(V_{\mathrm{lb}}; \lambda)$ yield the eigenvalues $\lambda_1(\mu_1, \sigma_\ell)$, $\lambda_2(\mu_1, \sigma_\ell), \ldots, \lambda_{N_\lambda}(\mu_1, \sigma_\ell)$, cf. the respectively attached axes in Fig. 7A.

3. Use the computed eigenvalues $\lambda_n(\mu_1, \sigma_\ell)$ as initial approximations $\tilde{\lambda}_n$ for the target eigenvalues at the next larger mean input, $\lambda_n(\mu_2, \sigma_\ell)$ and iteratively solve Eq. (C-1) with Powell's hybrid method. Note this can yield a complex eigenvalue for a real initialization (close to the real-to-complex transition).

4. Repeat the last step by taking the eigenvalues at μ_{k-1} as initial approximation for μ_k where $k = 3, \ldots, M_\mu$.

Since this procedure is independent of the sequential σ_ℓ (and n) order it can be computed in parallel for $\ell = 1, \ldots, M_\sigma$ (and $n = 1, \ldots, N_\lambda$).

The nonlinear solver, Powell's hybrid method, approximates the Jacobian of the equivalent two-dimensional real nonlinear system of the complex function $q(V_{\mathrm{lb}}; \lambda)$

with finite difference step size $\Delta\lambda$ and stops iterating when the relative convergence tolerance ε is reached implying a solution has been found. Since this root finding method (that we apply to solve Eq. (C-1)) converges locally, the input parameter rectangle has to be discretized sufficiently fine, i.e, $\Delta\mu$ and $\Delta\sigma$ have to be small. Otherwise artefacts as jumps between the eigenvalue curves could occur, especially when $\lambda_n(\mu)$ is steep (cf. Fig. 7A) and another eigenvalue is close by (in λ space).

We use for the solver parameters the values of Table C-1, which are suitable for the network model of this study as parametrized by Table 1 of the main text.

Table C-1: **Parameter values of the spectral solver.**

Name	Symbol	Value
Spacing of input rectangle, mean input	$\Delta\mu$	0.005 mV/ms[1]
Spacing of input rectangle, standard dev.	$\Delta\sigma$	0.1 mV/$\sqrt{\text{ms}}$
Membrane voltage discretization width	ΔV	0.01 mV
Smallest mean input (real spectrum)	μ_{min}	-1.5 mV/ms
Number of eigenvalues	N_λ	10
Finite difference step size (MINPACK: EPS)	$\Delta\lambda$	1e-10 kHz
Relative convergence tolerance (MINPACK: XTOL)	ε	1e-8
Finite difference step size (quantities), mean input	$\delta\mu$	0.001 mV/ms
Finite difference step size (quantities), standard dev.	$\delta\sigma$	0.001 mV/$\sqrt{\text{ms}}$

[1]Fine spacing required (in comparison to the value of $\Delta\mu$ used for the quantity precalculation of the cascade based models) due to the continuous tracking of the eigenvalues. Note that after the calculation we downsample the spectrum to the same mean input resolution for comparability.

C3) Exponential integration

A major factor for efficiency and accuracy of the algorithm above is the particular numerical way in which the backward integration of the differential equation system (C-2) is performed since this corresponds to one evaluation of the nonlinear function $\lambda \mapsto q(V_{\text{lb}};\lambda)$. An efficient and accurate discretization scheme is to perform exponential integration steps, i.e.,

$$(q^{m-1}, \phi^{m-1})^T = \exp\left[\mathbf{A}\left(V_{m-\frac{1}{2}}\right)\Delta V\right](q^m, \phi^m)^T \qquad \text{(C-5)}$$

with $q^m = q_\phi(V_m)$ and $\phi^m = \phi(V_m)$ on an equidistant membrane voltage grid $V_m = V_{\text{lb}} + m\Delta V$ ($m = 0, \dots, N_V$ and $V_{N_V} = V_{\text{s}}$). This scheme involves the matrix exponential function, $\exp(\mathbf{A}\Delta V) = \sum_{j=0}^{\infty}(\mathbf{A}\Delta V)^j/j!$, that is inexpensively evaluated as an (equivalent) linear combination of $\mathbf{A}\Delta V$ and the identity matrix [72]. This second order convergent numerical integration scheme that exploits the linearity of the system, Eq. (C-2), is obtained by truncating the Magnus expansion of the exact solution after one term and approximating the occuring integral $\int_{V_{m-1}}^{V_m}\mathbf{A}(V)dV$ using the mid point rule [71]. Note that the matrices $\mathbf{A}(V)$ and $\mathbf{A}(\tilde{V})$ do not commute for $V \neq \tilde{V}$ in general which implies that the solution of Eq. (C-2) does not have a simple exponential representation but is rather described by an (infinite) Magnus series. For the perfect integrate-and-fire model, though, the scheme, Eq. (C-5), gives the exact solution of Eq. (C-2) as the coefficient matrix $\mathbf{A}$ is constant due to $g(V) = 0$ in this case.

The integration of Eq. (C-2) is initialized at V_s with $\phi^{N_V} = 0$ to satisfy the absorbing boundary, Eq. (C-3)), and with the arbitrary choice $q^{N_V} = 1$ (possible due to the linearity of both Eq. (C-2) and the boundary conds). The exponential scheme, Eq. (C-5), is then used to (backward) calculate (q^m, ϕ^m), $m = N_V - 1, \ldots, m_\mathrm{r}$, where the reset voltage is assumed to be contained in the grid, i.e., $V_{m_\mathrm{r}} = V_\mathrm{r}$. At the reset voltage V_r the (generalized) reinjection cond., Eq. (C-4), is applied by

$$q^{m_\mathrm{r}} \leftarrow q^{m_\mathrm{r}} - q^{N_V} \exp(-\lambda T_\mathrm{ref}). \tag{C-6}$$

Continuing the backward integration using the scheme of Eq. (C-5) again for $m = m_\mathrm{r} - 1, \ldots, 0$ finally gives the values q^0 and ϕ^0 at V_lb. Therefore, $q_0(\lambda) \stackrel{!}{=} 0$ corresponds to the root finding problem, Eq. (C-1), after (exponential) membrane voltage discretization, and a value of $q_0 = 0$ indicates that λ is an eigenvalue with respective eigenfunction $\phi(V)$ in discrete representation $(\phi^m, m = 0, \ldots, N_V)$.

C4) Adjoint operator

To calculate the eigenfunctions of the adjoint operator $\mathcal{L}^*$ (cf. Eq. (49)–(52)) we assume that an eigenvalue λ_n is given (obtained for example using the procedure described in the previous two sections). Eq. (49), i.e., $\mathcal{L}^*[\psi_n] = \lambda_n \psi_n$, can be rewritten as a linear second order system for $(\psi_n, d\psi_n)^T$,

$$\frac{d}{dV}\begin{pmatrix}\psi_n \\ d\psi_n\end{pmatrix} = \underbrace{\begin{pmatrix} 0 & 1 \\ \frac{2\lambda_n}{\sigma^2} & -2\frac{g(V)+\mu}{\sigma^2}\end{pmatrix}}_{=\mathbf{B}}\begin{pmatrix}\psi_n \\ d\psi_n\end{pmatrix} \tag{C-7}$$

with $d\psi_n = \partial_V \psi_n$ and (nonlinear) coefficient matrix $\mathbf{B}$. This system is exponentially integrated *forwards* from the lower bound V_lb to the spike voltage V_s. Specifically we define $\psi_n^m = \psi_n(V_m)$ and $d\psi_n^m = \partial_V \psi_n(V_m)$ on the same grid as in the previous section. The integration is initialized according to the boundary cond. at V_lb (cf. Eq. (51)), i.e., $d\psi_n^0 = 0$ together with the arbitrary choice $\psi_n^0 = 1$ due to the linearity of the problem. Then we calculate the values ψ_n^m, $d\psi_n^m$ $(m = 1, \ldots, N_V)$ using the exponential integration scheme

$$(\psi_n^{m+1}, d\psi_n^{m+1})^T = \exp\left[\mathbf{B}\left(V_{m+\frac{1}{2}}\right)\Delta V\right](\psi_n^m, d\psi_n^m)^T. \tag{C-8}$$

The (generalized) boundary cond., Eq. (61), $\psi_n^{N_V} = \psi_n^{m_r}\exp(-\lambda_n T_\mathrm{ref})$ is necessarily fullfilled because λ_n was assumed to be an eigenvalue. This implies that ψ_n^m is the corresponding (everywhere continuously differentiable, cf. main text) eigenfunction $\psi_n(V)$ in discrete form. Note that the generalized boundary condition above corresponds for $T_\mathrm{ref} = 0$ to the respective condition that does not include the refractory period, i.e., Eq. (50).

C5) Quantities

The quantities that are required by the spike rate models spec$_1$ (Eq. (59)), and spec$_2$ (Eq. (62)), i.e., λ_1, λ_2, r_∞, $\partial_x r_\infty$, $\langle V\rangle_\infty$, $\partial_x\langle V\rangle_\infty$, f_n, c_n^x, for $x = \mu, \sigma^2$ and $n = 1, 2$, are calculated for each mean μ_k and standard deviation σ_ℓ of the input rectangle as follows.

Applying the exponential integration scheme Eqs. (C-5),(C-6) for the eigenvalue $\lambda_0 = 0$ gives the (unnormalized) eigenfunction $\hat{\phi}_0$ which is proportional to the stationary distribution p_∞. After normalizing $\hat{\phi}_0$ to yield a probability density, i.e., $\phi_0 = \hat{\phi}_0 / \int_{V_\mathrm{lb}}^{V_\mathrm{s}} \hat{\phi}_0(V)dV$, the stationary quantities, mean membrane voltage

$\langle V\rangle_\infty = \int_{V_{\rm lb}}^{V_{\rm s}} V\phi_0(V)dV$ and spike rate $r_\infty = q_{\phi_0}(V_{\rm s})$, are calculated. Practically, the latter is given by the (scaled) flux initialization of the exponential backward integration, $r_\infty = q^{N_V} / \int_{V_{\rm lb}}^{V_{\rm s}} \hat{\phi}_0(V)dV$, which is – for a refractory period $T_{\rm ref} > 0$ – denoted with $\tilde{r}_\infty$ giving after scaling the steady-state spike rate $r_\infty = \tilde{r}_\infty/(1 + \tilde{r}_\infty T_{\rm ref})$. The optional spike shape extension can be incorporated in this case using Eq. (93) with $p_\infty = \phi_0/(1 + \tilde{r}_\infty T_{\rm ref})$.

To obtain the first two dominant eigenvalues λ_1 and λ_2 we use the procedure of Sects. C2), C3) and calculate a number N_λ of (nonstationary) eigenvalues $\lambda_n(\mu_k, \sigma_\ell)$, $n = 1, \ldots, N_\lambda$ for the given input parameter rectangle. These eigenvalues are sorted, for each input parameter pair (μ_k, σ_ℓ) separately, such that λ_1 and λ_2 are the first and second dominant eigenvalue, respectively, according to Eqs. (58),(67), cf. Fig. 7A,C. Note that the other eigenvalues, $n = 3, \ldots, N_\lambda$, are not used for the models spec$_1$ and spec$_2$. However, they are required within the numerical solution method to account for the points in input parameter space (μ, σ), where the eigenvalue class switches (due to $V_{\rm lb} \neq V_{\rm r}$, see main text). For example in Fig. 7A (right column, i.e., with large noise intensity σ) a diffusive mode is dominant for small mean input μ while for increased mean the dominant eigenvalue (pair) is from the regular type. The numerical procedure described above starts with the dominant N_λ (real) eigenvalues at the smallest mean input and then continuously tracks each of these eigenvalues for increasing mean input μ. Therefore, when computing only, e.g., $N_\lambda = 2$ eigenvalues for the previous example both would be of the diffusive kind and the dominant regular modes for larger μ cannot be found.

The nonstationary quantities are based on the (already calculated) dominant eigenvalues λ_1 and λ_2. First the corresponding (unnormalized) eigenfunctions $\tilde{\phi}_1$ and $\tilde{\phi}_2$ of $\mathcal{L}$ are obtained using the exponential integration scheme, Eqs. (C-5),(C-6) with $\lambda = \lambda_1, \lambda_2$, as well as those of $\mathcal{L}^*$ (ψ_1 and ψ_2) that are computed via Eq. (C-8). The eigenfunctions of $\mathcal{L}$ are then scaled according to $\phi_n = \tilde{\phi}_n/\langle\psi_n, \tilde{\phi}_n\rangle$ which yields (bi)orthonormal eigenfunctions, i.e., $\langle\psi_n, \phi_m\rangle = \delta_{nm}$, and this fixes the remaining degree of freedom for products between quantities of $\mathcal{L}$ and $\mathcal{L}^*$, e.g., $c_n^{\sigma^2} f_n$. Note that in the spec$_2$ model, Eqs. (62)–(66), nonstationary quantities occur exclusively in such products, specifically, $\mathbf{f} \cdot \mathbf{c}_x = c_1^x f_1 + c_2^x f_2$ and $\mathbf{f} \cdot \mathbf{\Lambda}\, \mathbf{c}_x = c_1^x f_1 \lambda_1 + c_2^x f_2 \lambda_2$ (for $x = \mu, \sigma^2$), and they do not enter at all the spec$_1$ model, Eq. (59), except for the first dominant eigenvalue λ_1.

The nonstationary quantities of $\mathcal{L}$ are obtained by $f_1 = q_{\phi_1}(V_{\rm s})$ and $f_2 = q_{\phi_2}(V_{\rm s})$, and particularly (similar to r_∞ above) by "reading off" the respective (normalized) initialization values $f_n = q^{N_V}/\langle\psi_n, \tilde{\phi}_n\rangle$.

The other quantities (nonstationary of $\mathcal{L}^*$ and the remaining stationary ones) involve partial derivatives w.r.t. μ and σ^2. They are calculated using a central finite difference approximation that is second order accurate (in the respective step size $\delta\mu$ or $\delta\sigma$), $\partial_\mu\theta \approx [\theta_{\mu+\delta\mu,\sigma} - \theta_{\mu-\delta\mu,\sigma}]/(2\delta\mu)$ and $\partial_\sigma\theta \approx (\theta_{\mu,\sigma+\delta\sigma} - \theta_{\mu,\sigma-\delta\sigma})/(2\delta\sigma)$ for $\theta = r_\infty$, $\langle V\rangle$, $\psi_n(V)$. For implementation convenience we calculate σ-derivatives of the quantities and transform them, using the chain rule, to the originally required ones, $\partial_{\sigma^2}\theta = \partial_\sigma\theta/(2\sigma)$. For the (final) quantities $c_n^x = \langle\partial_x\psi_n,\, \phi_0\rangle$ the stationary eigenfunction ϕ_0 is multiplied with the finite difference version of $\partial_x\psi_n$. The latter requires for each $x = \mu, \sigma^2$ two (forward) integrations via Eq. (C-8) (e.g., $\psi_n(V; \mu + \delta\mu, \sigma)$ and $\psi_n(V; \mu - \delta\mu, \sigma)$).

C6) Modifications

The solutions of Eq. (C-2) or (C-7) can be multiplied with an arbitrary complex scalar value to yield another solution because the operators $\mathcal{L}$ and $\mathcal{L}^*$ and the corresponding boundary conds. are linear in ϕ, q or ψ, $\partial_V\psi$ respectively. Therefore the initializations

q^{N_V} or ψ^0 of the exponential integration schemes, Eqs. (C-5) or (C-8), can be chosen arbitrarily and the (bi)normalization is applied a posteriori. In our numerical implementation we specifically initialize with $q^{N_V} = 1$ only at $\mu_{\min}$ and for all other $\mu_k > \mu_{\min}$ we start the integration with $q^{N_V}(\mu_k) = q^{N_V}(\mu_{k-1})$ where $q^{N_V}(\mu_{k-1})$ is taken after normalization. This modification allows to specify tolerance and finite difference parameters, ε and $\Delta\lambda$, respectively, that are appropriate for the whole input rectangle despite the fact that the magnitude of the function which is evaluated in each step of the root finding algorithm, i.e., $q(V_{\mathrm{lb}}; \lambda)$, depends strongly on (μ, σ, λ) (e.g., see the scales of the attached axes in Fig. 7A).

The numerical solver described above does not take into account the fact that eigenvalues λ_n at the transition from real to complex values have multiplicity two. Therefore, at these input parameter points (μ^*, σ^*) we calculate all nonstationary quantities by nearest-neighbor interpolation to resolve corresponding artefacts. Note that an even more pronounced smoothing of the quantities around these points would likely be beneficial to the model performance of the model spec_2.
