# Supplementary material for: Low-dimensional spike rate models derived from networks of adaptive integrate-and-fire neurons: Comparison and implementation
Source: PLoS Comput Biol. 2017 Jun 23;13(6):e1005545. doi: 10.1371/journal.pcbi.1005545 (PMC5507472; doi:10.1371/journal.pcbi.1005545)
Supplement: S1 Fig — (PDF) [file pcbi.1005545.s002.pdf]

Low-dimensional spike rate models derived from networks of adaptive integrate-and-fire neurons: comparison and implementation

Moritz Augustin[1,2,*], Josef Ladenbauer[1,2,3,*], Fabian Baumann[1,2], Klaus Obermayer[1,2]

1 Department of Software Engineering and Theoretical Computer Science, Technische Universität Berlin, Germany
2 Bernstein Center for Computational Neuroscience Berlin, Germany
3 Group for Neural Theory, Laboratoire de Neurosciences Cognitives, École Normale Supérieure, Paris, France

*** augustin@ni.tu-berlin.de and josef.ladenbauer@tu-berlin.de**

S1 Figure: Fast changes of the input variance

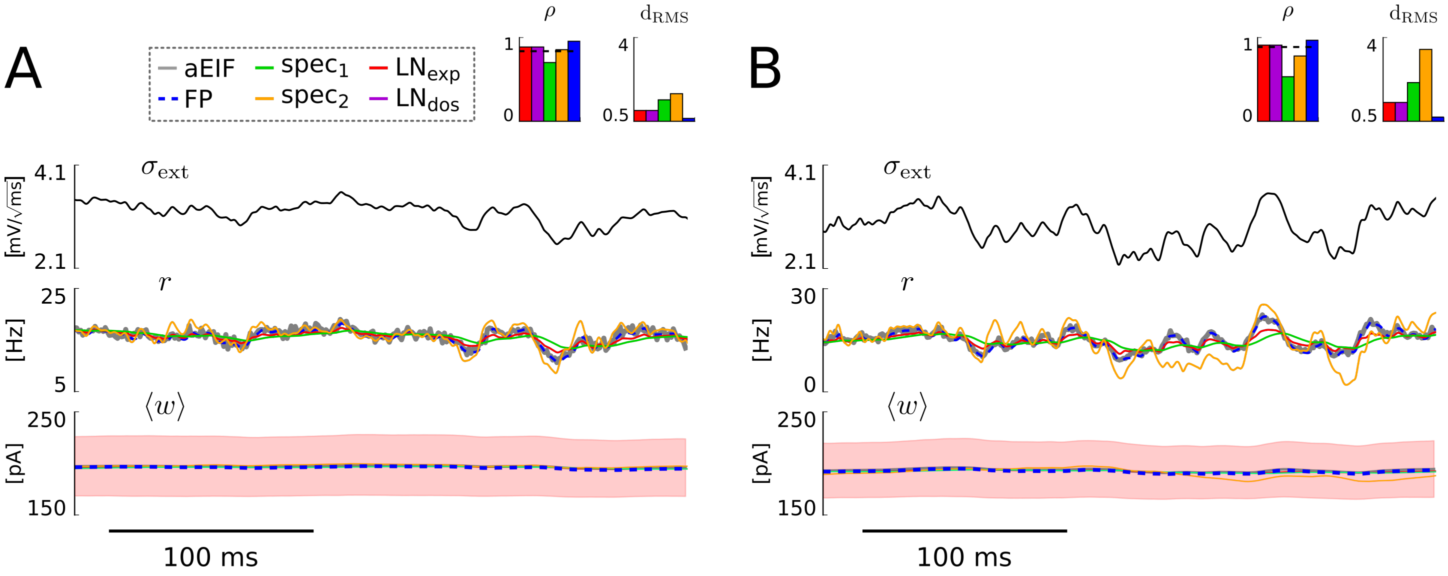

Fast changes of the input variance. Time series of population spike rate and mean adaptation current from the different models in response to weak mean $\mu_{\text{ext}} = 1.5$ mV/ms and time-varying variance σ^2_{ext} of the input for moderately fast variations $\tau_{\text{ou}}^{\sigma^2} = 50$ ms (**A**) and rapid variations $\tau_{\text{ou}}^{\sigma^2} = 10$ ms (**B**). The values for the remaining parameters and the visualization style were as in Fig. 4B of the main text which corresponds to **A** here, except that a different realization of the OU process was used.
